# Extensive survey of the *ycf*4 plastid gene throughout the IRLC legumes: Robust evidence of its locus and lineage specific accelerated rate of evolution, pseudogenization and gene loss in the tribe Fabeae

**DOI:** 10.1371/journal.pone.0229846

**Published:** 2020-03-05

**Authors:** Mahtab Moghaddam, Shahrokh Kazempour-Osaloo

**Affiliations:** Department of Plant Biology, Faculty of Biological Sciences, Tarbiat Modares University, Tehran, Iran; Chinese Academy of Medical Sciences and Peking Union Medical College, CHINA

## Abstract

The genome organization and gene content of plastome (plastid genome) are highly conserved among most flowering plant species. Plastome variation (in size and gene order) is rare in photosynthetic species but size variation, rearrangements and gene/intron losses is attributed to groups of seed plants. Fabaceae (legume family), in particular the subfamily Papilionoideae and the inverted repeat lacking clade (IRLC), a largest legume lineage, display the most dramatic and structural change which providing an excellent model for understanding of mechanisms of genomic evolution. The IRLC comprises 52 genera and ca 4000 species divided into seven tribes. In present study, we have sampled several representatives from each tribe across the IRLC from various herbaria and field. The *ycf*4 gene, which plays a role in regulating and assembly of photosystem I, is more variable in the tribe Fabeae than in other tribes. In certain species of *Lathyrus*, *Pisum* and *Vavilovia*, all belonging to Fabeae, the gene is either absent or a pseudogene. Our study suggests that *ycf*4 gene has undergone positive selection. Furthermore, the rapid evolution of the gene is locus and lineage specific and is not a shared character of the IRLC in legumes.

## Introduction

Previous gene mapping and genomic sequencing has demonstrated that variation in plastid genome (plastome) size, gene order and gene/intron content is relatively rare. Plastome organization in most of angiosperms is identical which comprises two large (~ 25 kb) copies of inverted repeat (IR) regions separated by two single copy regions, the large (~ 85 kb) single copy region (LSC) and the small (~ 15 kb) single copy region (SSC) [1–3]. Due to the conserved nature of plastid genome, variation at the nucleotide and structural level can provide powerful phylogenetic markers and allow a comparative evaluation of genomic evolutionary history [3, 22]. There are several unrelated angiosperm lineages that have been identified with different structural rearrangements such as inversion, transposition, gene duplication, gene/intron loss, insertion/deletion and IR expansion/contraction [3–4]. Variation in plastome size is often related to IR expansion/contraction [4–6]. Expansion in IR size have been documented in *Pelargonium* x *hortorum* (217 942 bp) from the Geraniaceae which is Known as the largest seed plant plastome size [5].Cases of IR contraction can be found in Geraniaceae [7] and Pinaceae [8]. Some angiosperm families like Orobanchaceae and Fabaceae show IR loss in their plastomes [9–11].

Fabaceae (legumes) are the third largest family of angiosperms which have experienced a remarkable number of plastome rearrangements [12]. Currently accepted classification of the legumes based on plastid gene *mat*K includes six subfamilies: Caesalpinioideae, Cercidoideae, Detarioideae, Dialioideae, Duparquetioideae, and Papilionoideae [13]. Gene order and gene/intron content in plastomes of all subfamilies except Papilionoideae are highly conserved and similar to the ancestral angiosperm genome organization [6]. Papilionoideae, the largest group of legumes due to its ecological and economical importance, exhibit numerous rearrangements and gene/intron losses and have smaller genome [6, 14]. The remarkable loss of the one of the plastid IR in the inverted repeat lacking clade (IRLC), a largest legume lineage, is an example of genome variation in papilionoids [14–15]. This clade comprises 52 genera (e.g., *Wisteria*, *Glycyrrhiza*, *Astragalus*, *Colutea*, *Trifolium*, *Lathyrus*, …) and ca 4000 species divided into seven tribes [14, 16–18]. The existence of a diversity of changes in the organization and gene content of IRLC plastome have made it as an excellent model for genome evolution studies. To date, different rearrangements have been reported in various tribes of the IRLC. For example the introns of *rps*12 and *clp*P have been lost in most members of the IRLC [19–22] and in tribe Trifolieae, *acc*D gene has been transferred to the nucleus in some *Trifolium* species [22–24].

Within the IRLC, the plastid genome of tribe Fabeae (including five main genera: *Lathyrus* L., *Lens* Mill., *Vicia* L., *Pisum* L. and *Vavilovia* Fed.) exhibits variation in size and expansion/contraction which have occurred during its evolution, due to the presence of repetitive DNA [25–27]. Also, *ycf*4 gene in *Pisum sativum* and *Lathyrus odoratus*, is either absent or pseudogene [21].

The *ycf*4 gene (orf184) is located in LSC region and its product is a thylakoid protein which is involved in regulating the assembly of the photosystem I complex [4, 28–30]. It is a part of a gene cluster, including *psa*I and *acc*D genes at the upstream and *cem*A gene at the downstream of it, which is considered as local mutation hotspot in plastid genome [21]. This genomic region has undergone numerous rearrangements not only in the IRLC legumes (as mentioned above) but also in different lineages. For example; within *Jasminum* and *Menodora*, both of them are belonging Oleaceae, *acc*D have been lost and the *ycf*4-*psa*I region in *Jasminum* section *Primulina* was relocated [31], the *acc*D was found as a pseudogene in some species of *Primula* (Primulaceae) [32] and chloroplast genome of *Tylosema esculentum* as one of the basal legumes in the Caesalpinioideae, has an unique inverted region which is including six genes *rbc*L, *acc*D, *psa*I, *ycf*4, *cem*A and *pet*A [33].

Unusual evolution of *ycf*4 and genomic region around of it in legumes, especially in the IRLC, as well as relatively small number of IRLC samples in previous studies [6, 21–22], prompted us to evaluate the evolutionary history of *ycf*4 gene across the IRLC with special reference to the tribe Fabeae. Furthermore, in this study two other widely sequenced plastid genes, *mat*K and *rpl*32, were used for the comparison of nonsynonymous and synonymous nucleotide substitution rates. In this paper, we examined the following points: 1) to determine the presence or absence of *ycf*4 gene across the IRLC, 2) to assess phylogenetic utility of this gene at the tribal to generic and species levels across the IRLC, and 3) to investigate the evolutionary rate of *ycf*4 in the IRLC and Fabeae with comparing nonsynonymous and synonymous nucleotide substitution rates.

## Materials and methods

### Taxon sampling

Sampling was designed to include different species from across the IRLC [14]. The plastid *ycf*4 gene was obtained from representatives of IRLC genera not sampled previously and combined with a large number of sequences already in Genbank plus appropriate outgroups (*Lotus japonicus* and *Robinia pseudoacacia*) in Loteae and Robineae. In addition to the newly generated *ycf*4 (60), *mat*K (27) and *rpl*32 (24) gene sequences, 62 *ycf*4, 80 *mat*K and 72 *rpl*32 sequences were retrieved from GenBank. All taxa together with their origin, voucher information and GenBank accession numbers are listed in S1 Table.

### DNA extraction, amplification and sequencing

Total genomic DNA was isolated from leaf materials using the modified CTAB method of Doyle and Doyle [34]. The *ycf*4 gene was amplified using the primer of *acc*D and *cem*A [21] and also *Psa*I and *cem*A (designed in this study). The information (location and base composition) of each of the primers used in this study and a schema of the *acc*D-*cem*A regions with positions of forward and reverse primers are shown in S2 Table and S1 Fig, respectively.

The PCR amplifcation was carried out in the volume of 20 μl, containing 8 μl deionized water, 10 μl of the 2 × Taq DNA polymerase master mix Red (Amplicon) 0.5 μl of each primer (10 pmol/μl), and 1 μl of template DNA.PCR procedures for *ycf*4 region were 2 min at 94°C for predenaturation followed by 38 cycles of 1 min at 94°C for denaturation, 3 min 20 s at 58°C (when using *acc*D and *cem*A primers) and 50 s at 55°C (when using *Psa*I and *cem*A primers) for primer annealing and 50 s at 72°C for primer extension, followed by a final primer extension of 5 min at 72°C. The ensuring PCR fragments were separated by electrophoresis in 1% agarose gels in 1 × TAE (pH = 8) buffer, stained with ethidium bromide and were photographed with a UV gel documentation system (UVItec, Cambridge, UK). PCR products along with the primers used for amplifcation were sent for Sanger sequencing at Macrogen (Seoul, South Korea).

### Sequence alignment

*ycf*4 gene data set was aligned using the web-based version of MUSCLE [35] under default parameters followed by manual adjustment. Indels were treated as missing data in all phylogenetic analyses. Highly variable sites were excluded to construct datasets. Removing regions of the alignments that have a gap (some species in which the *ycf*4 is pseudogene and lacks start and stop codons) before performing PAML analysis is common. It is often done with the hope that the remaining sequence has a better quality alignment and, thus, the results are more reliable. In order to determine the coding range of *ycf*4 gene in some species, open reading frame (ORF) Finder (https://www.ncbi.nlm.nih.gov/orffinder/) was used. To calculate dN/dS, we will require codon-based alignments of the DNA sequences of all genes in each ortholog group; therefore gaps should be positioned so as not to change the reading frame. Among the aligners, we have used PRANK [36, 37], which is unique in that it takes evolutionary information into consideration during alignment to infer positive selection acting on genes and codons.

### Phylogenetic analysis

Bayesian phylogenetic analysis was conducted at the CIPRES Science Gateway V. 3.3 [38] using MrBayes version 3.2.6 [39] with default priors (uniform priors) and the best-fit model of sequence evolution for dataset. The Markov chain Monte Carlo algorithm was run in two separated analyses, each run with four simultaneous Markov chains (one cold and three heated with a heating parameter of 0.2) for 10 million generations, trees sampled at every 100 generations. The first 25,000 trees (25%) were discarded as a conservation burn-in, and the remaining trees were used to construct the 50% majority rule consensus tree with posterior probability values (PP). Stationary of the chains was ascertained using Tracer v.1.6 [40]. Tree visualization was carried out using Dendroscope v.2.7.4 [41].

In Bayesian analysis, the best model of molecular evolution of nucleotide substitutions was evaluated using MrModeltest 2.2 [42], based on the Akaike Information Criteria (AIC) [43]. On the basis of model test results, the GTR+G were identified as the best model for *ycf*4 gene.

### Selective pressure analysis

Nonsynonymous (dN) and synonymous (dS) substitution rates and the ratio (*ω* = dN/dS) between them which provide information about the evolutionary forces operating on a gene, in the codon-based nucleotide alignments of 3 chloroplast genes (*ycf*4, *mat*K, *rpl*32) in the IRLC and tribe Fabeae were estimated using yn00 from the PAML package [44] and JCoDA package [45] across branches. Furthermore, the inference of selection was performed using the branch-site models of CODEML algorithm [44] implemented in EasyCodeML [46]. In general, when positive selection dominates, *ω* is greater than 1, natural selection is acting to promote nonsynonymous substitutions and fixation of advantageous mutations and adaptive evolution would be inferred when dN>dS and when negative selection (also called purifying selection) dominates, *ω* is less than 1, natural selection suppresses protein changes and acting against deleterious nonsynonymous substitutions. In genetic regions under strong negative selection, mutations are quickly removed from the gene pool, resulting in highly conserved stretches of the genome [47–49]. Since dN and dS are usually estimated using complete coding sequences and we do not expect an entire gene to evolve under positive selection, it is very rare to see dN>dS and large structural rearrangement has seldom been observed in chloroplast genes. Under selective neutrality, *ω* is close to 1, the positive and negative selection forces balance each other and synonymous and nonsynonymous substitution rates should be equal. In other words, coding sequences of gene evolving under no influence of selection.

## Results

### Sequence considerations

**Fig 1:**
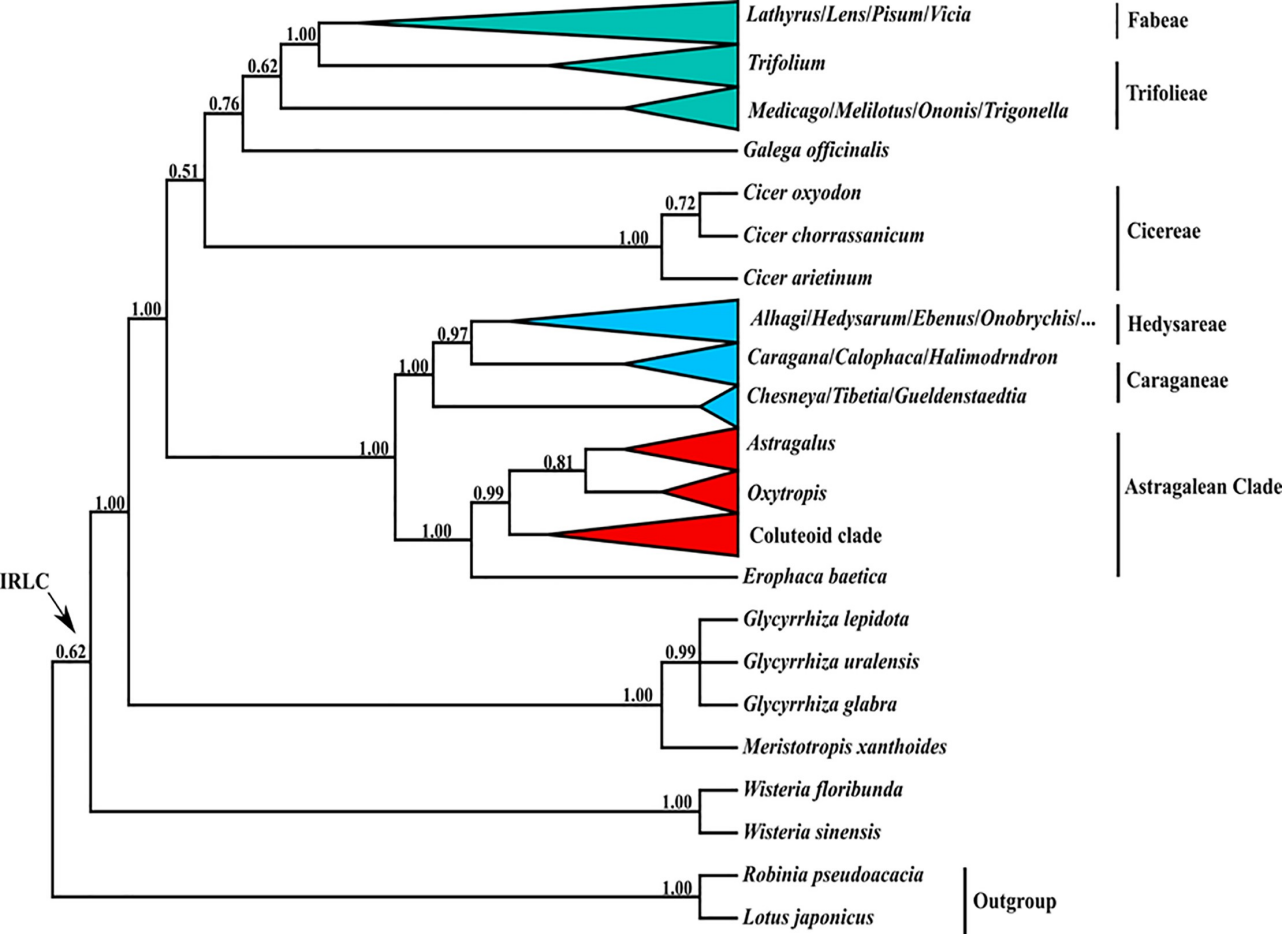
Fifty percent majority rule consensus tree resulting from Bayesian analysis of the *ycf*4 gene dataset. Numbers above branches are posterior probability values. Values <50% were not shown.

The aligned *ycf*4 dataset was 1128 nucleotide sites long in the IRLC (S1 File), of which 846 sites were potentially parsimony informative. The 50% majority rule consensus tree resulting from the Bayesian analysis of the *ycf*4 dataset for IR loss clade with posterior probabilities is shown in Fig 1. The monophyly of the IRLC and all its tribes is in accordance with all previous studies [14, 50]. The IRLC comprises all members of several well supported tribes/lineages including Cicereae, Hedysareae, Caraganeae, Trifolieae, Fabeae, Astragalean clade (including tribe Coluteae and genera *Astragalus*, *Oxytropis* and *Erophaca*) and *Galega* (Galegeae) as well as Wisterieae together with *Adinobotrys* and *Glycyrrhiza* [14, 16–18].

**Fig 2:**
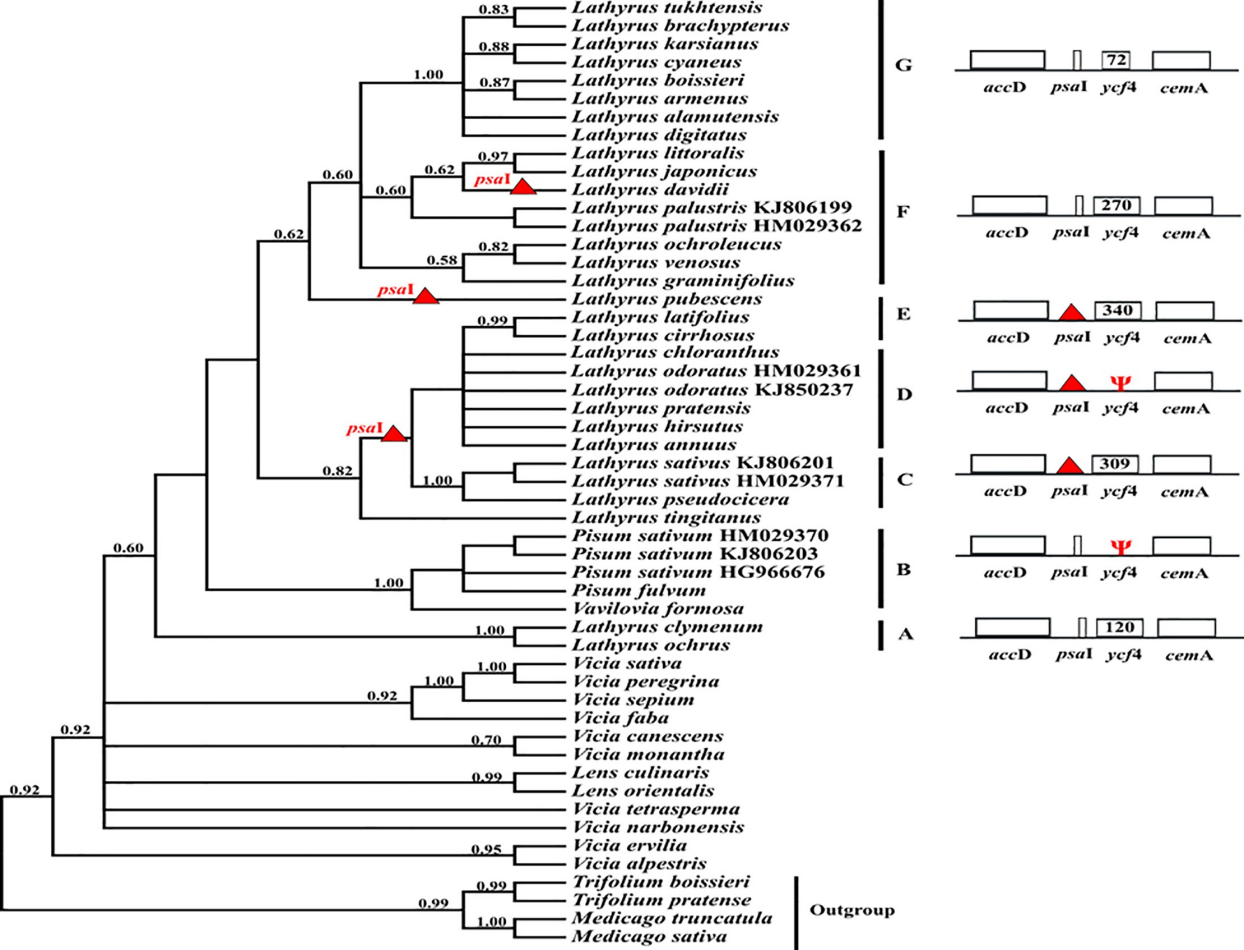
Presence and absence of the *ycf*4 and *psa*I genes in *Lathyrus*, *Pisum* and *Vavilovia*. Red triangles on branches indicate evolutionary losses of the *psa*I gene. Psi symbols denote pseudogenes. Numbers indicate the numbers of codons in *ycf*4 gene. The topology of the tree is the result of Bayesian analysis of tribe Fabeae based on *ycf*4 gene. Numbers above branches are posterior probability values. Values <50% were not shown.

We sequenced the region flanking the *ycf*4 locus for different samples of different tribes in the IRLC and compared it to the available data for other legumes. The length of *ycf*4 varies from 564–567 bp in *Astragalus*, *Oxytropis* and tribe Hedysareae (as the smaller length of *ycf*4 in the IRLC) to 630 bp in tribe Trifolieae. Gene length in various genera from different tribes of the IRLC does not show much difference except tribe Fabeae. In other words, in all tribes of the IRLC, except tribe Fabeae, *ycf*4 and its neighbors are conserved either in length or point mutations. *ycf*4 region shows extensive length variation among the Fabeae species that retain it. The aligned *ycf*4 dataset was 1085 nucleotide sites in Fabeae. As a result, the length of *ycf*4 in *Vicia* and *Lens* is 615 and 606 bp, respectively and in different species of *Lathyrus*, *Pisum* and *Vavilovia* is highly variable and in some cases is lost or pseudogene. Accordingly, they are divided into seven groups (A-G, Fig 2). In groups B, C, D, and E at least one of the two *psa*I and *ycf*4 genes was lost. In the present study, *ycf*4 gene in some species of *Lathyrus* (*L*. *chloranthus*, *L*. *hirsutus*, *L*. *pratensis*, *L*. *odoratus* and *L*. *annuus*), *Pisum* (*P*. *sativum* and *P*. *fulvum*) and *V*. *formosa* show signs of pseudogenization (groups B and D, Fig 2). Furthermore, *psa*I loss was detected in certain *Lathyrus* species (groups C, D and E, Fig 2).

### dN/dS analysis

**Fig 3:**
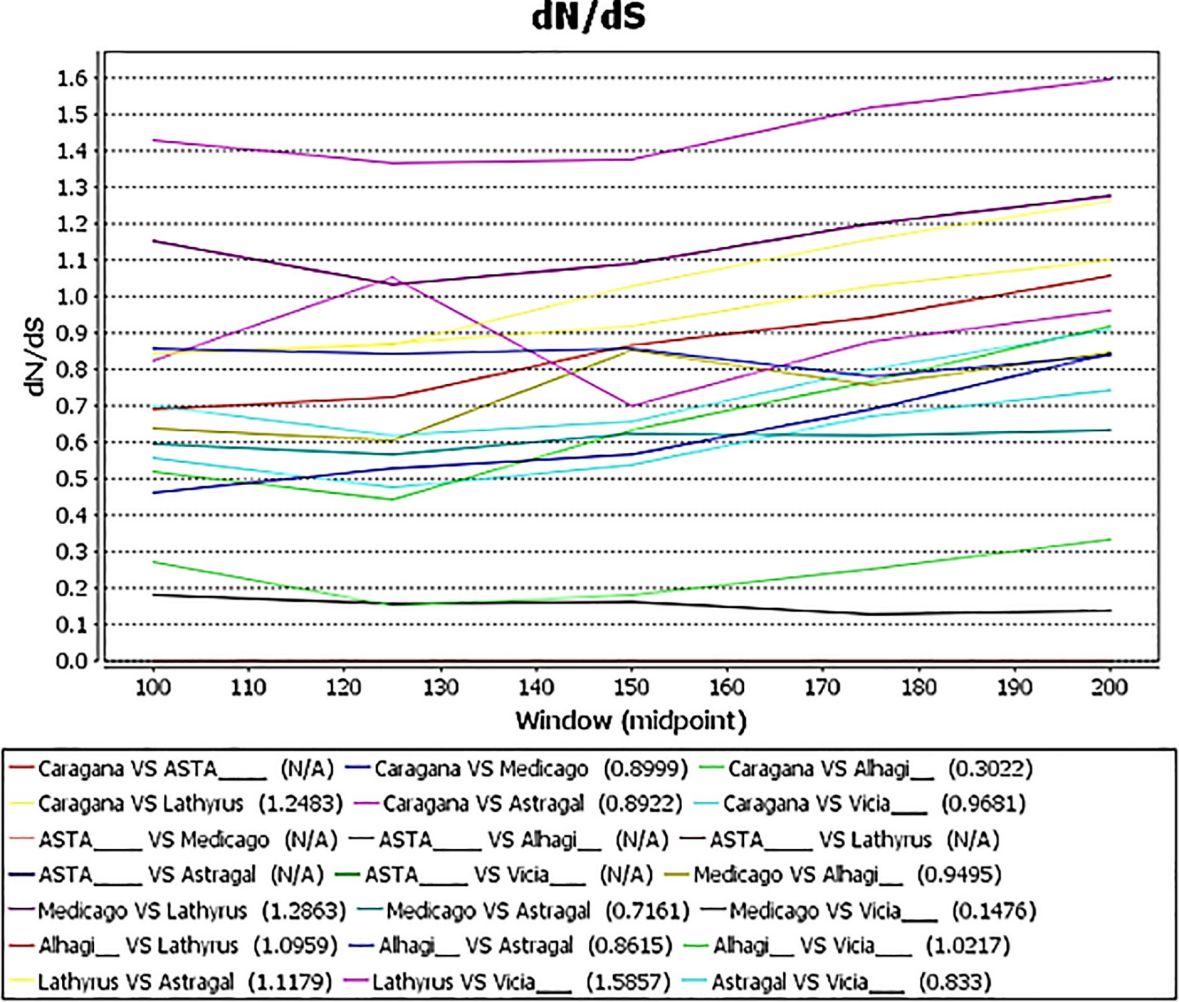
JCoDA output using sliding window analysis of pairwise taxa dN/dS. All parwise comparisons were performed using a window 100. All pairwise comparisons suggested positive selection of *ycf*4 in *Lathyrus*.

**Fig 4:**
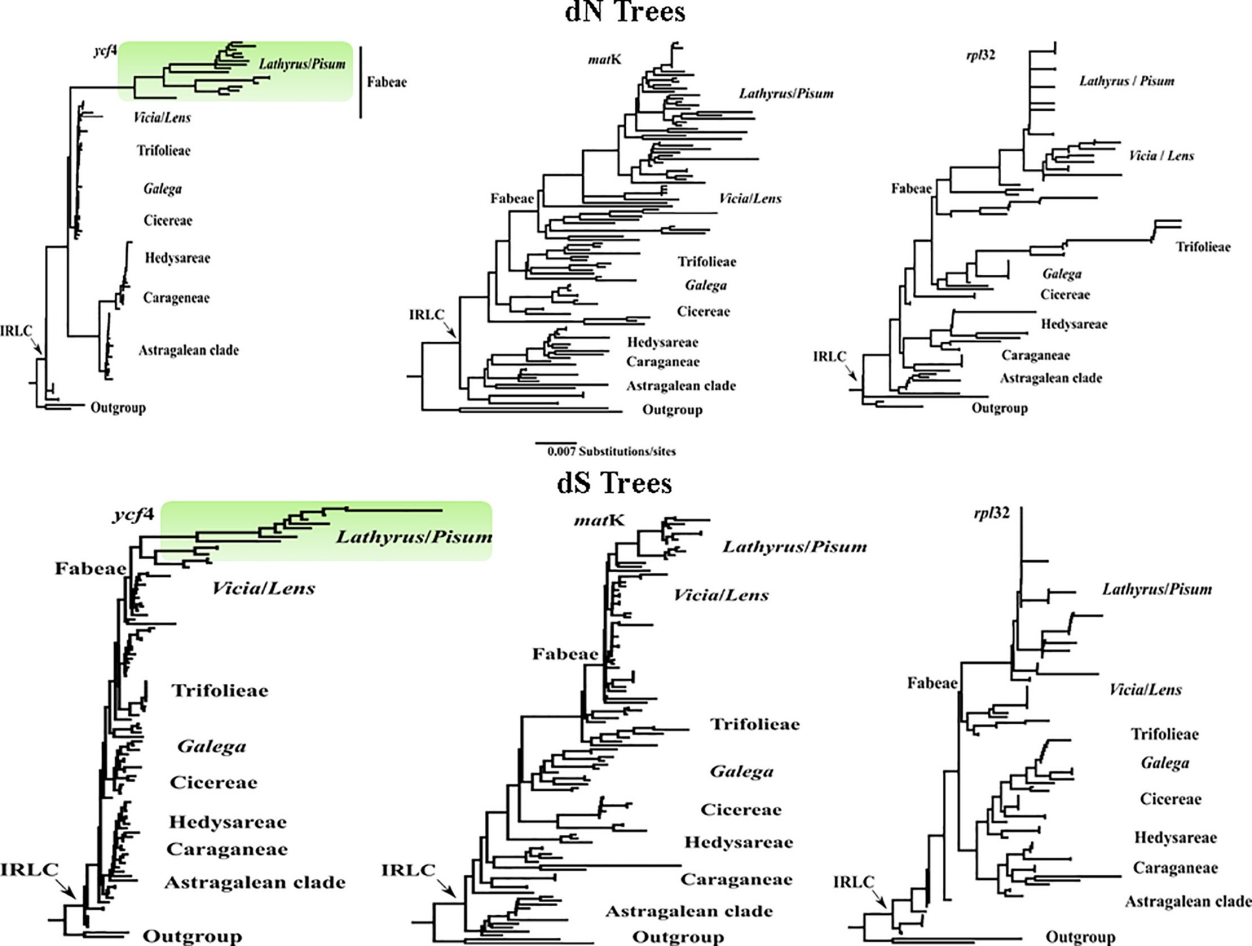
Synonymous and nonsynonymous divergence in IRLC *ycf*4 sequences. All trees are drawn to the same scale. Green branches in the dN and dS trees indicate taxa in which *ycf*4 gene represents positive selection and acceleration in evolutionary rate. Trees for *mat*K and *rpl*32 genes do not show comparable rate at either synonymous or nonsynonymous divergence.

For each gene, we used the method of Yang [44] to investigate the number of non-synonymous (dN) and synonymous (dS) nucleotide substitutions and the ratio of them (dN/dS, *ω*) across branches and found that this ratio varies significantly among lineages, under the tree topology resulting from Bayesian analysis. In the dN/dS analyses (Figs 3 and 4), acceleration of the evolutionary rate is seen in *ycf*4 in Fabeae, particularly *Lathyrus*, relative to other IRLC genera (Fig 3). The same high acceleration rate is, however, not seen in two other plastid genes (*mat*K and *rpl*32) across the IRLC (Fig 4). The level of constraint on *ycf*4 gene in *Lathyrus* is lower than in the other genera of IRLC. dN/dS ratio among the genera belonging to different tribes is less than that ratio among the related species of *Lathyrus*, for instance, dN/dS = 0.716 between *Medicago sativa* and *Astragalus membranaceous* compared with dN/dS = 1.527 within *L*. *davidii* and *L*. *littoralis*. In other words, *ycf*4 is highly conserved in all genera of the IRLC except *Lathyrus*.

We then applied the branch-site model, which accommodates heterogeneity among sites and can reflect divergent selective pressures. Parameter estimates under this model suggested that, when the *Lathyrus* branch was the foreground branch, some set of sites in *ycf*4 gene evolved under positive selective pressures (S3 Table). Using the Bayes empirical Bayes method, we identified seven codon sites in *ycf*4 gene with posterior probabilities ≥ 95% that evolved under positive selective pressure on the *Lathyrus* branch (1 L, 2 S, 3 V, 4 V, 5 L, 6 L, 7 T). When other branches from other genera were the foreground branches, no codon sites with posterior probabilities ≥ 95% were identified under positive selective pressures (S3 Table).

The *ycf*4 gene is highly variable in terms of length and point mutations compared to the other two genes among *Lathyrus* species (Table 1). The length of *ycf*4, among *Lathyrus* species which have the intact gene, varies from 219 bp in group G (including *L*. *tukhtensis*, *L*. *brachypterus*, *L*. *karsianus*, *L*. *cyaneus*, *L*. *boissieri*, *L*. *armenus*, *L*. *alamutensis* and *L*. *digitatus*) to 1023 bp in group E (including *L*. *latifolius* and *L*. *cirrhosus*). While the gene length in the other two genes (1512–1527 bp in *mat*K and 153–183 bp in *rpl*32) does not show much variation. Furthermore, the nucleotide substitution rates in *Lathyrus* species are clearly elevated in *ycf*4 when compared with other two cpDNA genes, for example, there are four nucleotide substitutions between *L*. *littoralis* and *L*. *japonicus* in *mat*K sequences and there are no differences between their *rpl*32 gene, however, there are 67 nucleotide substitutions between these two species in *ycf*4 gene. All of the investigated species of *Lathyrus* showed signs of elevated branch lengths in the *ycf*4 gene (Fig 4). Our analysis for estimation of positive selection revealed that *Lathyrus* branch within *ycf*4 data has undergone adaptive evolution and showed *ω* value greater than 1 (Table 1, Fig 4).

**Table 1:** Sequence divergence in cpDNA regions compared among *Lathyrus* species.

Genes	*L*.*sativus* vs. *L*.*ochroleucus*			*L*.*davidii* vs. *L*.*littoralis*			*L*.*odoratus* vs. *L*. *palustris*		
	d_N_/d_S_	d_N_ ± SE	d_S_ ± SE	d_N_/d_S_	d_N_ ± SE	d_S_ ± SE	d_N_/d_S_	d_N_ ± SE	d_S_ ± SE
*ycf*4	3.589	2.006±1.74	0.559±0.43	1.527	0.218±0.136	0.143±0.150	NA	NA	NA
*mat*K	0.390	0.020±0.00	0.052±0.01	0.218	0.008±0.00	0.003±0.00	0.349	0.021±0.00	0.060±0.01
*rpl*32	0.223	0.009±0.00	0.044±0.04	0.0	0.0	0.0	0.193	0.019±0.01	0.097±0.07

For protein-coding genes the synonymous divergence (dS), the nonsynonymous divergence (dN), its standard error (SE), and the nonsynonymous-to-synonymous ratio (dN/dS, also called *ω*) are shown. NA, Not applicable (gene not present).

## Discussion

### Phylogenetic estimations

Within the papilionoid legumes, many phylogenetic studies have been performed on the IRLC that confirm its monophyletic origin [14, 51]. The IRLC consists of several tribes (as mentioned above) [14, 18]. Tribe Fabeae comprises five genera (*Lathyrus*, *Pisum*, *Vicia*, *Lens*, *Vavilovia*) and c. 380 species and contains some important crop species. In present work in agreement with previous studies [52–54], Fabeae has monophyletic origin and is sister group to *Trifolium*. Like previous studies [52–54], our results confirm the monophyly of *Lens* and *Pisum* and the paraphyly of *Vicia* and *Lathyrus*. *Pisum* and *Vavilovia* are sister groups and nested in *Lathyrus*, which together with *Lens* is nested in *Vicia*.

Extensive phylogenetic analyses have been undertaken in the IRLC and tribe Fabeae, using mainly organellar or rDNA markers [6, 14, 53] which have resolved relationships between genera and even species at lower levels. In the present work, phylogenetic relationships have been investigated only based on *ycf*4 gene. This gene lacked adequate phylogenetic signal at the lower taxonomic level but showed better phylogenetic resolution at higher taxonomic level (generic to tribal rank). For example, in some systematic and phylogenetic studies [52–54] based on *mat*K and *rbc*L, the relationships between sections and species of *Lathyrus* have been carefully resolved, for instance, *L*. *odoratus* and *L*. *hirsutus* are closest relatives but in the present study the relationship between them is unclear due to low support value. In our study, the *ycf*4 gene did not resolve the relationships at low levels and it is suggested that *ycf*4 along with other plastid genes such as *mat*K, *rbc*L and *ndh*F be used for molecular phylogenetic studies of genera and species.

Sequence divergence value across the IRLC is less than 0.751 (0.000–0.750) and among *Lathyrus* species is less than 0.534 (0.000–0.533). Therefore, the low phylogenetic resolution at low taxonomic levels is due to the low number of synapomorphic characters and showed strong evidence of homoplasy. Homoplasy can be caused by different factors such as high selection pressures and mutation rates [55]. In *ycf*4 sequences, length and point mutations may cause homoplasy and lower phylogenetic resolution. The gene is not useful for resolving lower level relationships (e.g. at the level of species and lower).

The lack of *ycf*4 has evolved twice within the IRLC; once in the clade containing *Pisum* and *Vavilovia* specie (group B) and again in some *Lathyrus* species including *L*. *chloranthus*, *L*. *odoratus*, *L*. *pratensis*, *L*. *hirsutus* and *L*. *annuus* (group D). *psa*I gene also shows independent evolution three times in different species of *Lathyrus* (including *L*. *davidii*, *L*. *pubescens* and groups C/D/E).

Magee et al. [21] detected some previous false reports about *ycf*4 gene losses in four legume species (*Glycine max*, *Trifolium subterraneum*, *Cicer arietinum*, *Medicago truncatula*). We also noticed that the *ycf*4 gene which was not identified in cpDNA of *Medicago sativa* [56] and 13 *Lathyrus* species [57] is, in fact, present in the plastid genome of these species. These *Lathyrus* species are still referred without *ycf*4 gene [58]. We identified the gene in the plastome of the species using ORF finder and comparison with other related species. Due to the high evolutionary rate of the *ycf*4 gene and consequently the high divergence of gene, different softwares which are used to annotate like DOGMA [59] cannot recognize it.

### Positive selection and rapid evolution of *ycf*4

In the present work, we have investigated the evolutionary rate of the open reading frame *ycf*4 and its genomic region in the IRLC, and particularly in Fabeae. Our results showed that *ycf*4 gene and its upstream gene (*psa*I) are more variable in the tribe Fabeae than in other tribes of the IRLC. The *ycf*4 gene was found in all tribes of the IRLC except Fabeae completely intact and well conserved. In certain species of *Lathyrus*, *Pisum* and *Vavilovia*, *ycf*4 is either absent or pseudogenized. In legumes, in addition to *Lathyrus*, *ycf*4 shows signs of pseudogenization in *Desmodium heterocarpon* [60]. Moreover, the genomic region around *ycf*4 including *psa*I gene at the upstream of it, in some species of *Lathyrus* has been lost (Fig 2).

*ycf*4 gene encoded a thylakoid protein which is involved in the assembly of the photosystem I complex (PSI) as a part of an energy harvesting process. PSI embedded in the thylakoid membranes of phototrophs (cyanobacteria, algae and plants) and mediates the light-induced electron transfer from plastocyanin or cytochrome *c* to ferredoxin. PSI contains at least 11 subunits, 5 of which are encoded by the plastid genome (PsaA, PsaB, PsaC, PsaI and PsaJ) and other subunits (PsaD, PsaE, PsaF, PsaG, PsaH and PsaK) are nuclear-encoded subunits [4, 28, 61]. All of the plastid encoded subunits are transmembrane proteins with specific and important functional roles except subunits I and J. The function of these two subunits has not yet been determined and it seems that the presence of subunits I and J are not necessary for the PSI function [4, 28, 62]. *psa*I gene encodes a small protein which is conserved among land plants and *psa*I mutants in tobacco plants have standard growth conditions [63]. Our data demonstrated that *psa*I gene has been lost in some *Lathyrus* species (some of them also lack *ycf*4 gene such as group D in Fig 2). Therefore, according to the result of tobacco mutants, it is conceivable that *Lathyrus* species without *psa*I gene do not show abnormal phenotype and have standard growth. Some parasitic species like *Cuscuta gronovii*, *C*. *obtusiflora* (photosynthetic) and *Epifagus virginiana* (non-photosynthetic) and also some genera from green algae (*Chromera*, *Vitrella*, *Aureococcus*, *Bigelowiella natans* and *Euglena gracilis*) are other known examples in which *psa*I gene is absent [64–67].

In order to evaluate the function of *ycf*4 gene, different studies have generated stable knockout mutants for *ycf*4 [28, 63]. It is expected that the lack of *ycf*4 gene will be resulted in loss of PSI activity and reduce autotrophic growth. But the analysis of *ycf*4-deficient mutant appears that *ycf*4 is required for the assembly and stability of the PSI not for the synthesis of that. Ycf4 is one of the most important chaperons in PSI assembly process but the functional role of that has not yet been clearly identified [4, 68]. Accordingly, some studies [4, 30] have suggested renaming this factor to *paf*II (PSI assembly factor II). Krech et al. [63] have shown that Ycf4 protein is an important but non-essential factor for PSI assembly process. Given that all species which have lost these two genes are photosynthetic and certainly have a functional PSI, it seems that other alternative factors appear to perform *ycf*4 and *psa*I functions [63]. Our study suggests that the photosystem I complex of *Lathyrus*, *Pisum* and *Vavilovia* underwent unique structural changes. In the present study, by comparing the evolutionary rates of the three chloroplast genes, found that only *ycf*4 gene had dN/dS value > 1, indicating that this gene had undergone positive selection. This positive selection across the IRLC and Fabeae is only seen in *Lathyrus*, *Pisum* and *Vavilovia*. Thus, rapid evolution of *ycf*4 is locus and lineage specific and is not a shared character of the IRLC in legumes. The presence of fast-evolving protein gene in *Lathyrus* is probably due to the high point and length mutations rates that may result from repeated DNA breakage and repair [21, 69].

When a gene is lost from organellar genome, the probability given is that the organelle gene has been transferred from organelle to nuclear. In such cases, based on the ratio between the point mutation rates in the organelle and nuclear, it can be determined whether the gene has been transmitted [21, 70]. In this context, evolutionary transfer of *acc*D gene from the plastome of *Trifolium subterraneum* to the nucleus can be mentioned [24]. Magee et al. [21] showed that there are no nuclear copies for *ycf*4 and *psa*I in the *L*. *odoratus* and *P*. *sativum*, therefore, it can be concluded that these genes have not been transferred to the nucleus. Our study demonstrated that each three tandem genes *psa*I-*ycf*4-*cem*A is situated in a local mutation hotspot in particular within *Lathyrus*, resulting in dramatic acceleration of sequence evolution in some species and evolutionary gene losses in others [6, 21, 71]. Given that in addition to *ycf*4 and *psa*I genes, other genes such as *acc*D and *rps*16 in legumes have been lost or pseudogenized (a phenomenon that is very rare in other angiosperms), it is supposed that a hotspot might have been existed across the legume evolution and caused the acceleration of the *ycf*4 but the exact location of hotspot has varied.

## Conclusion

The present study investigated the presence/absence and evolutionary process of *ycf*4/*psa*I genes in the IRLC and particularly Fabeae. The *ycf*4 gene was found in all tribes of the IRLC except Fabeae completely intact and conserved. In certain species of *Lathyrus*, *Pisum* and *Vavilovia*, *ycf*4/*psa*I is either absent or pseudogenized. Tribe Fabeae comprises five genera (*Lathyrus*, *Pisum*, *Vicia*, *Lens*, *Vavilovia*) and c. 380 species and contains some important crop species. The *ycf*4 gene is highly variable in terms of length and point mutations compared to the other two genes (*mat*K and *rpl*32) among *Lathyrus* species. In the present study, by comparing the evolutionary rates of the three chloroplast genes, we found that only *ycf*4 gene had dN/dS value > 1, indicating that this gene had undergone positive selection. This positive selection across the IRLC and Fabeae is only seen in *Lathyrus*, *Pisum* and *Vavilovia*. Thus, rapid evolution of *ycf*4 is locus and lineage specific and is not a shared character of the IRLC in legumes.

## Supporting information

S1 Fig Relative position of the PCR amplification and sequencing primers used in this study. Arrows indicate the direction of strand synthesis. Boxed areas represent coding region.
(TIF)

S1 File Alignment of ycf4 gene sequences in IRLC legumes. (PDF)

S1 Table Taxa included in the *ycf*4, *mat*K and *rpl*32 analyses. (-) not available in GenBank. Abbreviations used in plant accession information: FMUH, Ferdowsi University of Mashhad Herbarium, Mashhad, Iran; GAZI, Gazi Universitesi Herbarium, Ankara, Turkey; IRAN, Iranian Research Institute of Plant Protection,Tehran, Iran; MO, Missouri Botanical Garden Herbarium, St Louis, USA; MSB Herbarium of Ludwig-Maximilians-Universitat, Munchen, Germany; TARI Herbarium of the Research Institute of Forests and Rangelands, Tehran, Iran; TMUH, Tarbiat Modares University Herbarium, Tehran, Iran; TUH, Tehran University Herbarium, Tehran, Iran; HWANRC Herbarium of West Azarbayjan Natural Resources Research Center, Urmia, Iran. ^a^Sequences from GenBank. ^b^Whole plastid genome.
(PDF)

S2 Table Location and base composition of amplification and sequencing primers used in this study. * Location indicates the Start and end nucleotide positions.
(PDF)

S3 Table Parameter estimation and likelihood ratio tests for the branch-site model. (PDF)
