# Supplementary figures and images for: Extensive survey of the ycf4 plastid gene throughout the IRLC legumes: Robust evidence of its locus and lineage specific accelerated rate of evolution, pseudogenization and gene loss in the tribe Fabeae
Source: PLoS One. 2020 Mar 5;15(3):e0229846. doi: 10.1371/journal.pone.0229846 (PMC7058334; doi:10.1371/journal.pone.0229846)

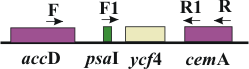

Supplement: S1 Fig — Arrows indicate the direction of strand synthesis. Boxed areas represent coding region. (TIF) [file pone.0229846.s001.tif]
