# Supplementary material for: Extensive survey of the ycf4 plastid gene throughout the IRLC legumes: Robust evidence of its locus and lineage specific accelerated rate of evolution, pseudogenization and gene loss in the tribe Fabeae
Source: PLoS One. 2020 Mar 5;15(3):e0229846. doi: 10.1371/journal.pone.0229846 (PMC7058334; doi:10.1371/journal.pone.0229846)
Supplement: S1 File — (PDF) [file pone.0229846.s002.pdf]

```
Alignment: C:\Users\Mahtab\Desktop\IRLC_YCF4.nxs
Seaview [blocks=10 fontsize=10 A4] on Tue Dec 24 17:09:44 2019

                           1
Trifolium strictum         ---------- ---------- ---------- ---------- ----------
T. glanduliferum           ---------- ---------- ---------- ---------- ----------
T. boissieri               ---------- ---------- ---------- ---------- ----------
T. aureum                  ---------- ---------- ---------- ---------- ----------
T.meduseum                 ---------- ---------- ---------- ---------- ----------
T.subterraneum             ---------- ---------- ---------- ---------- ----------
T.pratense                 ---------- ---------- ---------- ---------- ----------
T.hybridum                 ---------- ---------- ---------- ---------- ----------
T.semipilosum              ---------- ---------- ---------- ---------- ----------
T.occidentale              ---------- ---------- ---------- ---------- ----------
T.repens                   ---------- ---------- ---------- ---------- ----------
T.lupinaster               ---------- ---------- ---------- ---------- ----------
Cicer oxyodon              ---------- ---------- ---------- ---------- ----------
C. chorrassanicum          ---------- ---------- ---------- ---------- ----------
C. arietinum               ---------- ---------- ---------- ---------- ----------
Medicago truncatula_KF     ---------- ---------- ---------- ---------- ----------
M.truncatula_AC            ---------- ---------- ---------- ---------- ----------
M.sativa_K                 ---------- ---------- ---------- ---------- ----------
M.sativa                   ---------- ---------- ---------- ---------- ----------
M.papillosa                ---------- ---------- ---------- ---------- ----------
M.hybrida                  ---------- ---------- ---------- ---------- ----------
M.falcata                  ---------- ---------- ---------- ---------- ----------
M.sativa_KU                ---------- ---------- ---------- ---------- ----------
Melilotus albus            ---------- ---------- ---------- ---------- ----------
Hedysarum formosum         ---------- ---------- ---------- ---------- ----------
H.varium                   ---------- ---------- ---------- ---------- ----------
H.singarense               ---------- ---------- ---------- ---------- ----------
H.minjanense               ---------- ---------- ---------- ---------- ----------
Tavrniera glabra           ---------- ---------- ---------- ---------- ----------
T.diffusa                  ---------- ---------- ---------- ---------- ----------
Greuteria membranacea      ---------- ---------- ---------- ---------- ----------
Corethrodendron scoparium  ---------- ---------- ---------- ---------- ----------
Eversmannia subspinosa     ---------- ---------- ---------- ---------- ----------
Onobrychis bungei          ---------- ---------- ---------- ---------- ----------
O.cornuta                  ---------- ---------- ---------- ---------- ----------
O.michauxii                ---------- ---------- ---------- ---------- ----------
O.subacaulis               ---------- ---------- ---------- ---------- ----------
O.teheranica               ---------- ---------- ---------- ---------- ----------
Alhagi maurorum            ---------- ---------- ---------- ---------- ----------
Caragana microphylla       ---------- ---------- ---------- ---------- ----------
C.korshinski               ---------- ---------- ---------- ---------- ----------
C.kozlowii                 ---------- ---------- ---------- ---------- ----------
C.rosea                    ---------- ---------- ---------- ---------- ----------
Tibetia liangshanensis     ---------- ---------- ---------- ---------- ----------
Gueldenstaedtia verna      ---------- ---------- ---------- ---------- ----------
Halimodendron halodendron  ---------- ---------- ---------- ---------- ----------
Smirnowia turkestana       ---------- ---------- ---------- ---------- ----------
Eremosparton flaccidum     ---------- ---------- ---------- ---------- ----------
Colutea persica            ---------- ---------- ---------- ---------- ----------
C. triphylla               ---------- ---------- ---------- ---------- ----------
Sphaerophysa salsula       ---------- ---------- ---------- ---------- ----------
Podlechiella vogelii       ---------- ---------- ---------- ---------- ----------
Carmichaelia australis     ---------- ---------- ---------- ---------- ----------
Sutherlandia frutescens    ---------- ---------- ---------- ---------- ----------
Astragalus membranaceous   ---------- ---------- ---------- ---------- ----------
A.mongholicus              ---------- ---------- ---------- ---------- ----------
A.nakaianus                ---------- ---------- ---------- ---------- ----------
A.macropelmatus            ---------- ---------- ---------- ---------- ----------
A.iranicus                 ---------- ---------- ---------- ---------- ----------
A.denudatus                ---------- ---------- ---------- ---------- ----------
A.odoratus                 ---------- ---------- ---------- ---------- ----------
Oxytropis szovitsii        ---------- ---------- ---------- ---------- ----------
O.iranica                  ---------- ---------- ---------- ---------- ----------
O.kotschyana               ---------- ---------- ---------- ---------- ----------
Erophaca baetica           ---------- ---------- ---------- ---------- ----------
Wisteria floribunda        ---------- ---------- ---------- ---------- ----------
W. sinensis                ---------- ---------- ---------- ---------- ----------
Glycyrrhiza lepidota       ---------- ---------- ---------- ---------- ----------
G.uralensis                ---------- ---------- ---------- ---------- ----------
G.glabra                   ---------- ---------- ---------- ---------- ----------
Meristotropis xathioides   ---------- ---------- ---------- ---------- ----------
Robinia pseudoacacia       ---------- ---------- ---------- ---------- ----------
Lotus japonicus            ---------- ---------- ---------- ---------- ----------
Vicia alpestris            ---------- ---------- ---------- ---------- ----------
V.sativa                   ---------- ---------- ---------- ---------- ----------
```

```
V.peregrina               ---------- ---------- ---------- ---------- ----------
V.sepium                  ---------- ---------- ---------- ---------- ----------
V. faba                   ---------- ---------- ---------- ---------- ----------
V.canescens               ---------- ---------- ---------- ---------- ----------
V.monantha                ---------- ---------- ---------- ---------- ----------
V.tetrasperma             ---------- ---------- ---------- ---------- ----------
V.narbonensis             ---------- ---------- ---------- ---------- ----------
Lens culinaris            ---------- ---------- ---------- ---------- ----------
L.orientalis              ---------- ---------- ---------- ---------- ----------
V.ervilia                 ---------- ---------- ---------- ---------- ----------
Galega officinalis        ---------- ---------- ---------- ---------- ----------
Lathyrus palustris_K      ---------- ---------- ---------- ---------- ----------
L.palustris_H             ---------- ---------- ---------- ---------- ----------
L.davidii                 ---------- ---------- ---------- ---------- ----------
L.japonicus               ---------- ---------- ---------- ---------- ----------
L.ochroleucus             ---------- ---------- ---------- ---------- ----------
L.littoralis              ---------- ---------- ---------- ---------- ----------
L.venosus                 ---------- ---------- ---------- ---------- ----------
L.graminifolius           ---------- ---------- ---------- ---------- ----------
L.pubescens               ---------- ---------- ---------- ---------- ----------
L.sativus_KJ              ---------- ---------- ---------- ---------- ----------
L.sativus_HM              ---------- ---------- ---------- ---------- ----------
L.pseudocicera            ---------- ---------- ---------- ---------- ----------
L.chloranthus             ---------- ---------- ---------- ---------- ----------
L.pratensis               ---------- ---------- ---------- ---------- ----------
L.odoratus_HM             ---------- ---------- ---------- ---------- ----------
L.odoratus_kJ             ---------- ---------- ---------- ---------- ----------
L.hirsutus                ---------- ---------- ---------- ---------- ----------
L.annuus                  ---------- ---------- ---------- ---------- ----------
L.cirrhosus               ATGAACATAA AGCAGTGGTT TCACAGGATA AAGCAAGGGT GCCAAAGGAT
L.latifolius              ATGGACATAA AACAGTGGTT CCACAGGATA AAGCAAGGGT TCCAAAGGAT
L.tingitanus              ---------- ---------- ---------- ---------- ----------
Pisum sativum_H           ---------- ---------- ---------- ---------- ----------
Pisum sativum_K           ---------- ---------- ---------- ---------- ----------
Pisum sativum_HG          ---------- ---------- ---------- ---------- ----------
Pisum fulvum              ---------- ---------- ---------- ---------- ----------
Vavilovia formosa         ---------- ---------- ---------- ---------- ----------
L.clymenum                ---------- ---------- ---------- ---------- ----------
L.ochrus                  ---------- ---------- ---------- ---------- ----------
```

```
Trifolium strictum          ---------- ---------- ---------- ---------- ----------
T. glanduliferum            ---------- ---------- ---------- ---------- ----------
T. boissieri                ---------- ---------- ---------- ---------- ----------
T. aureum                   ---------- ---------- ---------- ---------- ----------
T.meduseum                  ---------- ---------- ---------- ---------- ----------
T.subterraneum              ---------- ---------- ---------- ---------- ----------
T.pratense                  ---------- ---------- ---------- ---------- ----------
T.hybridum                  ---------- ---------- ---------- ---------- ----------
T.semipilosum               ---------- ---------- ---------- ---------- ----------
T.occidentale               ---------- ---------- ---------- ---------- ----------
T.repens                    ---------- ---------- ---------- ---------- ----------
T.lupinaster                ---------- ---------- ---------- ---------- ----------
Cicer oxyodon               ---------- ---------- ---------- ---------- ----------
C. chorrassanicum           ---------- ---------- ---------- ---------- ----------
C. arietinum                ---------- ---------- ---------- ---------- ----------
Medicago truncatula_KF      ---------- ---------- ---------- ---------- ----------
M.truncatula_AC             ---------- ---------- ---------- ---------- ----------
M.sativa_K                  ---------- ---------- ---------- ---------- ----------
M.sativa                    ---------- ---------- ---------- ---------- ----------
M.papillosa                 ---------- ---------- ---------- ---------- ----------
M.hybrida                   ---------- ---------- ---------- ---------- ----------
M.falcata                   ---------- ---------- ---------- ---------- ----------
M.sativa_KU                 ---------- ---------- ---------- ---------- ----------
Melilotus albus             ---------- ---------- ---------- ---------- ----------
Hedysarum formosum          ---------- ---------- ---------- ---------- ----------
H.varium                    ---------- ---------- ---------- ---------- ----------
H.singarense                ---------- ---------- ---------- ---------- ----------
H.minjanense                ---------- ---------- ---------- ---------- ----------
Tavrniera glabra            ---------- ---------- ---------- ---------- ----------
T.diffusa                   ---------- ---------- ---------- ---------- ----------
Greuteria membranacea       ---------- ---------- ---------- ---------- ----------
Corethrodendron scoparium   ---------- ---------- ---------- ---------- ----------
Eversmannia subspinosa      ---------- ---------- ---------- ---------- ----------
Onobrychis bungei           ---------- ---------- ---------- ---------- ----------
O.cornuta                   ---------- ---------- ---------- ---------- ----------
O.michauxii                 ---------- ---------- ---------- ---------- ----------
O.subacaulis                ---------- ---------- ---------- ---------- ----------
O.teheranica                ---------- ---------- ---------- ---------- ----------
Alhagi maurorum             ---------- ---------- ---------- ---------- ----------
Caragana microphylla        ---------- ---------- ---------- ---------- ----------
C.korshinski                ---------- ---------- ---------- ---------- ----------
C.kozlowii                  ---------- ---------- ---------- ---------- ----------
C.rosea                     ---------- ---------- ---------- ---------- ----------
Tibetia liangshanensis      ---------- ---------- ---------- ---------- ----------
Gueldenstaedtia verna       ---------- ---------- ---------- ---------- ----------
Halimodendron halodendron   ---------- ---------- ---------- ---------- ----------
Smirnowia turkestana        ---------- ---------- ---------- ---------- ----------
Eremosparton flaccidum      ---------- ---------- ---------- ---------- ----------
Colutea persica             ---------- ---------- ---------- ---------- ----------
C. triphylla                ---------- ---------- ---------- ---------- ----------
Sphaerophysa salsula        ---------- ---------- ---------- ---------- ----------
Podlechiella vogelii        ---------- ---------- ---------- ---------- ----------
Carmichaelia australis      ---------- ---------- ---------- ---------- ----------
Sutherlandia frutescens     ---------- ---------- ---------- ---------- ----------
Astragalus membranaceous    ---------- ---------- ---------- ---------- ----------
A.mongholicus               ---------- ---------- ---------- ---------- ----------
A.nakaianus                 ---------- ---------- ---------- ---------- ----------
A.macropelmatus             ---------- ---------- ---------- ---------- ----------
A.iranicus                  ---------- ---------- ---------- ---------- ----------
A.denudatus                 ---------- ---------- ---------- ---------- ----------
A.odoratus                  ---------- ---------- ---------- ---------- ----------
Oxytropis szovitsii         ---------- ---------- ---------- ---------- ----------
O.iranica                   ---------- ---------- ---------- ---------- ----------
O.kotschyana                ---------- ---------- ---------- ---------- ----------
Erophaca baetica            ---------- ---------- ---------- ---------- ----------
Wisteria floribunda         ---------- ---------- ---------- ---------- ----------
W. sinensis                 ---------- ---------- ---------- ---------- ----------
Glycyrrhiza lepidota        ---------- ---------- ---------- ---------- ----------
G.uralensis                 ---------- ---------- ---------- ---------- ----------
G.glabra                    ---------- ---------- ---------- ---------- ----------
Meristotropis xathioides    ---------- ---------- ---------- ---------- ----------
Robinia pseudoacacia        ---------- ---------- ---------- ---------- ----------
Lotus japonicus             ---------- ---------- ---------- ---------- ----------
Vicia alpestris             ---------- ---------- ---------- ---------- ----------
V.sativa                    ---------- ---------- ---------- ---------- ----------
V.peregrina                 ---------- ---------- ---------- ---------- ----------
V.sepium                    ---------- ---------- ---------- ---------- ----------
V. faba                     ---------- ---------- ---------- ---------- ----------
```

```
V.canescens               ---------- ---------- ---------- ---------- ----------
V.monantha                ---------- ---------- ---------- ---------- ----------
V.tetrasperma             ---------- ---------- ---------- ---------- ----------
V.narbonensis             ---------- ---------- ---------- ---------- ----------
Lens culinaris            ---------- ---------- ---------- ---------- ----------
L.orientalis              ---------- ---------- ---------- ---------- ----------
V.ervilia                 ---------- ---------- ---------- ---------- ----------
Galega officinalis        ---------- ---------- ---------- ---------- ----------
Lathyrus palustris_K      ---------- ---------- ---------- ---------- ----------
L.palustris_H             ---------- ---------- ---------- ---------- ----------
L.davidii                 ---------- ---------- ---------- ---------- ----------
L.japonicus               ---------- ---------- ---------- ---------- ----------
L.ochroleucus             ---------- ---------- ---------- ---------- ----------
L.littoralis              ---------- ---------- ---------- ---------- ----------
L.venosus                 ---------- ---------- ---------- ---------- ----------
L.graminifolius           ---------- ---------- ---------- ---------- ----------
L.pubescens               ---------- ---------- ---------- ---------- ----------
L.sativus_KJ              ---------- --------AT GGATCTAAAA GACTTGAAAA GATTTTTGAA
L.sativus_HM              ---------- --------AT GGATCTAAAA GACTTGAAAA GATTTTTGAA
L.pseudocicera            ---------- ---------- ---------- ---------- ----------
L.chloranthus             ---------- ---------- ---------- ---------- ----------
L.pratensis               ---------- ---------- ---------- ---------- ----------
L.odoratus_HM             ---------- ---------- ---------- ---------- ----------
L.odoratus_kJ             ---------- ---------- ---------- ---------- ----------
L.hirsutus                ---------- ---------- ---------- ---------- ----------
L.annuus                  ---------- ---------- ---------- ---------- ----------
L.cirrhosus               TTGGCAGAAT ATCAGATCCT TCTTCTCAAA AACTGAAACA AAAAACGGAG
L.latifolius              TTGGCAGAAT ATCAGATCCT TCTTCTCAAA AACTGAAAAA ACAAATGGAG
L.tingitanus              ---------- ---------- ---------- ---------- ----------
Pisum sativum_H           ---------- ---------- ---------- ---------- ----------
Pisum sativum_K           ---------- ---------- ---------- ---------- ----------
Pisum sativum_HG          ---------- ---------- ---------- ---------- ----------
Pisum fulvum              ---------- ---------- ---------- ---------- ----------
Vavilovia formosa         ---------- ---------- ---------- ---------- ----------
L.clymenum                ---------- ---------- ---------- ---------- ----------
L.ochrus                  ---------- ---------- ---------- ---------- ----------
```

```
Trifolium strictum        ---------- ---------- ---------- ---------- ----------
T. glanduliferum          ---------- ---------- ---------- ---------- ----------
T. boissieri              ---------- ---------- ---------- ---------- ----------
T. aureum                 ---------- ---------- ---------- ---------- ----------
T.meduseum                ---------- ---------- ---------- ---------- ----------
T.subterraneum            ---------- ---------- ---------- ---------- ----------
T.pratense                ---------- ---------- ---------- ---------- ----------
T.hybridum                ---------- ---------- ---------- ---------- ----------
T.semipilosum             ---------- ---------- ---------- ---------- ----------
T.occidentale             ---------- ---------- ---------- ---------- ----------
T.repens                  ---------- ---------- ---------- ---------- ----------
T.lupinaster              ---------- ---------- ---------- ---------- ----------
Cicer oxyodon             ---------- ---------- ---------- ---------- ----------
C. chorrassanicum         ---------- ---------- ---------- ---------- ----------
C. arietinum              ---------- ---------- ---------- ---------- ----------
Medicago truncatula_KF    ---------- ---------- ---------- ---------- ----------
M.truncatula_AC           ---------- ---------- ---------- ---------- ----------
M.sativa_K                ---------- ---------- ---------- ---------- ----------
M.sativa                  ---------- ---------- ---------- ---------- ----------
M.papillosa               ---------- ---------- ---------- ---------- ----------
M.hybrida                 ---------- ---------- ---------- ---------- ----------
M.falcata                 ---------- ---------- ---------- ---------- ----------
M.sativa_KU               ---------- ---------- ---------- ---------- ----------
Melilotus albus           ---------- ---------- ---------- ---------- ----------
Hedysarum formosum        ---------- ---------- ---------- ---------- ----------
H.varium                  ---------- ---------- ---------- ---------- ----------
H.singarense              ---------- ---------- ---------- ---------- ----------
H.minjanense              ---------- ---------- ---------- ---------- ----------
Tavrniera glabra          ---------- ---------- ---------- ---------- ----------
T.diffusa                 ---------- ---------- ---------- ---------- ----------
Greuteria membranacea     ---------- ---------- ---------- ---------- ----------
Corethrodendron scoparium ---------- ---------- ---------- ---------- ----------
Eversmannia subspinosa    ---------- ---------- ---------- ---------- ----------
Onobrychis bungei         ---------- ---------- ---------- ---------- ----------
O.cornuta                 ---------- ---------- ---------- ---------- ----------
O.michauxii               ---------- ---------- ---------- ---------- ----------
O.subacaulis              ---------- ---------- ---------- ---------- ----------
O.teheranica              ---------- ---------- ---------- ---------- ----------
Alhagi maurorum           ---------- ---------- ---------- ---------- ----------
Caragana microphylla      ---------- ---------- ---------- ---------- ----------
C.korshinski              ---------- ---------- ---------- ---------- ----------
C.kozlowii                ---------- ---------- ---------- ---------- ----------
C.rosea                   ---------- ---------- ---------- ---------- ----------
Tibetia liangshanensis    ---------- ---------- ---------- ---------- ----------
Gueldenstaedtia verna     ---------- ---------- ---------- ---------- ----------
Halimodendron halodendron ---------- ---------- ---------- ---------- ----------
Smirnowia turkestana      ---------- ---------- ---------- ---------- ----------
Eremosparton flaccidum    ---------- ---------- ---------- ---------- ----------
Colutea persica           ---------- ---------- ---------- ---------- ----------
C. triphylla              ---------- ---------- ---------- ---------- ----------
Sphaerophysa salsula      ---------- ---------- ---------- ---------- ----------
Podlechiella vogelii      ---------- ---------- ---------- ---------- ----------
Carmichaelia australis    ---------- ---------- ---------- ---------- ----------
Sutherlandia frutescens   ---------- ---------- ---------- ---------- ----------
Astragalus membranaceous  ---------- ---------- ---------- ---------- ----------
A.mongholicus             ---------- ---------- ---------- ---------- ----------
A.nakaianus               ---------- ---------- ---------- ---------- ----------
A.macropelmatus           ---------- ---------- ---------- ---------- ----------
A.iranicus                ---------- ---------- ---------- ---------- ----------
A.denudatus               ---------- ---------- ---------- ---------- ----------
A.odoratus                ---------- ---------- ---------- ---------- ----------
Oxytropis szovitsii       ---------- ---------- ---------- ---------- ----------
O.iranica                 ---------- ---------- ---------- ---------- ----------
O.kotschyana              ---------- ---------- ---------- ---------- ----------
Erophaca baetica          ---------- ---------- ---------- ---------- ----------
Wisteria floribunda       ---------- ---------- ---------- ---------- ----------
W. sinensis               ---------- ---------- ---------- ---------- ----------
Glycyrrhiza lepidota      ---------- ---------- ---------- ---------- ----------
G.uralensis               ---------- ---------- ---------- ---------- ----------
G.glabra                  ---------- ---------- ---------- ---------- ----------
Meristotropis xathioides  ---------- ---------- ---------- ---------- ----------
Robinia pseudoacacia      ---------- ---------- ---------- ---------- ----------
Lotus japonicus           ---------- ---------- ---------- ---------- ----------
Vicia alpestris           ---------- ---------- ---------- ---------- ----------
V.sativa                  ---------- ---------- ---------- ---------- ----------
V.peregrina               ---------- ---------- ---------- ---------- ----------
V.sepium                  ---------- ---------- ---------- ---------- ----------
V. faba                   ---------- ---------- ---------- ---------- ----------
```

```
V.canescens                ---------- ---------- ---------- ---------- ----------
V.monantha                 ---------- ---------- ---------- ---------- ----------
V.tetrasperma              ---------- ---------- ---------- ---------- ----------
V.narbonensis              ---------- ---------- ---------- ---------- ----------
Lens culinaris             ---------- ---------- ---------- ---------- ----------
L.orientalis               ---------- ---------- ---------- ---------- ----------
V.ervilia                  ---------- ---------- ---------- ---------- ----------
Galega officinalis         ---------- ---------- ---------- ---------- ----------
Lathyrus palustris_K       ---------- ---------- ---------- ---------- ----------
L.palustris_H              ---------- ---------- ---------- ---------- ----------
L.davidii                  ---------- ---------- ---------- ---------- ----------
L.japonicus                ---------- ---------- ---------- ---------- ----------
L.ochroleucus              ---------- ---------- ---------- ---------- ----------
L.littoralis               ---------- ---------- ---------- ---------- ----------
L.venosus                  ---------- ---------- ---------- ---------- ----------
L.graminifolius            ---------- ---------- ---------- ---------- ----------
L.pubescens                ---------- ---------- ---------- ---------- ----------
L.sativus_KJ               AAACCTGTGG ATCTGGAAAA AAAGACACTG TAAAAGATTT TTGAAAAACC
L.sativus_HM               AAACCTGTGG ATCTGGAAAA AAAGACACTG TAAAAGATTT TTGAAAAACC
L.pseudocicera             ---------- ---------- ---------- ---------- ----------
L.chloranthus              ---------- ---------- ---------- ---------- ----------
L.pratensis                ---------- ---------- ---------- ---------- ----------
L.odoratus_HM              ---------- ---------- ---------- ---------- ----------
L.odoratus_kJ              ---------- ---------- ---------- ---------- ----------
L.hirsutus                 ---------- ---------- ---------- ---------- ----------
L.annuus                   ---------- ---------- ---------- ---------- ----------
L.cirrhosus                GGCATGTTAT TTCTTTTATG AACAAAGAGT TCAAAAAGAT CTTGGAAAAG
L.latifolius               GACATGTTAT TTCTTTTATG AACAAAGAGT TCCAAAAGAT CTTTTCCAAG
L.tingitanus               ---------- ---------- ---------- ---------- ----------
Pisum sativum_H            ---------- ---------- ---------- ---------- ----------
Pisum sativum_K            ---------- ---------- ---------- ---------- ----------
Pisum sativum_HG           ---------- ---------- ---------- ---------- ----------
Pisum fulvum               ---------- ---------- ---------- ---------- ----------
Vavilovia formosa          ---------- ---------- ---------- ---------- ----------
L.clymenum                 ---------- ---------- ---------- ---------- ----------
L.ochrus                   ---------- ---------- ---------- ---------- ----------
```

```
Trifolium strictum         ---------- ---------- ---------- ---------- ----------
T. glanduliferum           ---------- ---------- ---------- ---------- ----------
T. boissieri               ---------- ---------- ---------- ---------- ----------
T. aureum                  ---------- ---------- ---------- ---------- ----------
T.meduseum                 ---------- ---------- ---------- ---------- ----------
T.subterraneum             ---------- ---------- ---------- ---------- ----------
T.pratense                 ---------- ---------- ---------- ---------- ----------
T.hybridum                 ---------- ---------- ---------- ---------- ----------
T.semipilosum              ---------- ---------- ---------- ---------- ----------
T.occidentale              ---------- ---------- ---------- ---------- ----------
T.repens                   ---------- ---------- ---------- ---------- ----------
T.lupinaster               ---------- ---------- ---------- ---------- ----------
Cicer oxyodon              ---------- ---------- ---------- ---------- ----------
C. chorrassanicum          ---------- ---------- ---------- ---------- ----------
C. arietinum               ---------- ---------- ---------- ---------- ----------
Medicago truncatula_KF     ---------- ---------- ---------- ---------- ----------
M.truncatula_AC            ---------- ---------- ---------- ---------- ----------
M.sativa_K                 ---------- ---------- ---------- ---------- ----------
M.sativa                   ---------- ---------- ---------- ---------- ----------
M.papillosa                ---------- ---------- ---------- ---------- ----------
M.hybrida                  ---------- ---------- ---------- ---------- ----------
M.falcata                  ---------- ---------- ---------- ---------- ----------
M.sativa_KU                ---------- ---------- ---------- ---------- ----------
Melilotus albus            ---------- ---------- ---------- ---------- ----------
Hedysarum formosum         ---------- ---------- ---------- ---------- ----------
H.varium                   ---------- ---------- ---------- ---------- ----------
H.singarense               ---------- ---------- ---------- ---------- ----------
H.minjanense               ---------- ---------- ---------- ---------- ----------
Tavrniera glabra           ---------- ---------- ---------- ---------- ----------
T.diffusa                  ---------- ---------- ---------- ---------- ----------
Greuteria membranacea      ---------- ---------- ---------- ---------- ----------
Corethrodendron scoparium  ---------- ---------- ---------- ---------- ----------
Eversmannia subspinosa     ---------- ---------- ---------- ---------- ----------
Onobrychis bungei          ---------- ---------- ---------- ---------- ----------
O.cornuta                  ---------- ---------- ---------- ---------- ----------
O.michauxii                ---------- ---------- ---------- ---------- ----------
O.subacaulis               ---------- ---------- ---------- ---------- ----------
O.teheranica               ---------- ---------- ---------- ---------- ----------
Alhagi maurorum            ---------- ---------- ---------- ---------- ----------
Caragana microphylla       ---------- ---------- ---------- ---------- ----------
C.korshinski               ---------- ---------- ---------- ---------- ----------
C.kozlowii                 ---------- ---------- ---------- ---------- ----------
C.rosea                    ---------- ---------- ---------- ---------- ----------
Tibetia liangshanensis     ---------- ---------- ---------- ---------- ----------
Gueldenstaedtia verna      ---------- ---------- ---------- ---------- ----------
Halimodendron halodendron  ---------- ---------- ---------- ---------- ----------
Smirnowia turkestana       ---------- ---------- ---------- ---------- ----------
Eremosparton flaccidum     ---------- ---------- ---------- ---------- ----------
Colutea persica            ---------- ---------- ---------- ---------- ----------
C. triphylla               ---------- ---------- ---------- ---------- ----------
Sphaerophysa salsula       ---------- ---------- ---------- ---------- ----------
Podlechiella vogelii       ---------- ---------- ---------- ---------- ----------
Carmichaelia australis     ---------- ---------- ---------- ---------- ----------
Sutherlandia frutescens    ---------- ---------- ---------- ---------- ----------
Astragalus membranaceous   ---------- ---------- ---------- ---------- ----------
A.mongholicus              ---------- ---------- ---------- ---------- ----------
A.nakaianus                ---------- ---------- ---------- ---------- ----------
A.macropelmatus            ---------- ---------- ---------- ---------- ----------
A.iranicus                 ---------- ---------- ---------- ---------- ----------
A.denudatus                ---------- ---------- ---------- ---------- ----------
A.odoratus                 ---------- ---------- ---------- ---------- ----------
Oxytropis szovitsii        ---------- ---------- ---------- ---------- ----------
O.iranica                  ---------- ---------- ---------- ---------- ----------
O.kotschyana               ---------- ---------- ---------- ---------- ----------
Erophaca baetica           ---------- ---------- ---------- ---------- ----------
Wisteria floribunda        ---------- ---------- ---------- ---------- ----------
W. sinensis                ---------- ---------- ---------- ---------- ----------
Glycyrrhiza lepidota       ---------- ---------- ---------- ---------- ----------
G.uralensis                ---------- ---------- ---------- ---------- ----------
G.glabra                   ---------- ---------- ---------- ---------- ----------
Meristotropis xathioides   ---------- ---------- ---------- ---------- ----------
Robinia pseudoacacia       ---------- ---------- ---------- ---------- ----------
Lotus japonicus            ---------- ---------- ---------- ---------- ----------
Vicia alpestris            ---------- ---------- ---------- ---------- ----------
V.sativa                   ---------- ---------- ---------- ---------- ----------
V.peregrina                ---------- ---------- ---------- ---------- ----------
V.sepium                   ---------- ---------- ---------- ---------- ----------
V. faba                    ---------- ---------- ---------- ---------- ----------
```

```
V.canescens               ---------- ---------- ---------- ---------- ----------
V.monantha                ---------- ---------- ---------- ---------- ----------
V.tetrasperma             ---------- ---------- ---------- ---------- ----------
V.narbonensis             ---------- ---------- ---------- ---------- ----------
Lens culinaris            ---------- ---------- ---------- ---------- ----------
L.orientalis              ---------- ---------- ---------- ---------- ----------
V.ervilia                 ---------- ---------- ---------- ---------- ----------
Galega officinalis        ---------- ---------- ---------- ---------- ----------
Lathyrus palustris_K      -------ATG AATAAAAAA- --------AA AATTGTTCTA GTTATCGCCC
L.palustris_H             -------ATG AATAAAAAA- --------AA AATACTTCTA GTTATCATTC
L.davidii                 -------ATG AAAAAAAAAT TTAGTCTAAA AATTCTTAGA GTTTTGACTC
L.japonicus               -------ATG AAAAAAAAA- --------AA AAATTTTCTA GTTATCACAC
L.ochroleucus             -------ATG ACTACTAAA- -----AAGAA AATTTTTCTC GTTATTGCGC
L.littoralis              ---------- ---ATGAAA- --------AA AATATTTCTA GTTATTACAC
L.venosus                 -------ATG GAAAAAAAA- --------AA AATTTTGCTC GTTATCACCC
L.graminifolius           ---------- ATGAAAAAA- --------CA AGTTGCGCTA GTTATGGTGC
L.pubescens               -------ATG AATAAAAAGT ---ACAAAAA AGTGGTTTTG ATTATAGTTA
L.sativus_KJ              TGTGGATCTG GAAAAAAAGA CACTGTATTA TTATTACAGT GACACATATT
L.sativus_HM              TGTGGATCTG GAAAAAAAGA CACTGTATTA TTATTACAGT GACACATATT
L.pseudocicera            ---------- ---------- ---------- ---------- ----------
L.chloranthus             ---------- ---------- ---------- ---------- ----------
L.pratensis               ---------- ---------- ---------- ---------- ----------
L.odoratus_HM             ---------- ---------- ---------- ---------- ----------
L.odoratus_kJ             ---------- ---------- ---------- ---------- ----------
L.hirsutus                ---------- ---------- ---------- ---------- ----------
L.annuus                  ---------- ---------- ---------- ---------- ----------
L.cirrhosus               ATCTTTTTCA AGATAAAGAT CGTTTTCTTG ATCTTGAAAA AAAGGAAAAT
L.latifolius              ATCTTTTTCA AGATAAAGAT CGTTTCCAAG ATTTTGAAAA AAAGGAAAAT
L.tingitanus              ---------- ---------- ---------- ---------- ----------
Pisum sativum_H           ---------- ---------- ---------- ---------- ----------
Pisum sativum_K           ---------- ---------- ---------- ---------- ----------
Pisum sativum_HG          ---------- ---------- ---------- ---------- ----------
Pisum fulvum              ---------- ---------- ---------- ---------- ----------
Vavilovia formosa         ---------- ---------- ---------- ---------- ----------
L.clymenum                ---------- ---------- ---------- ---------- ----------
L.ochrus                  ---------- ---------- ---------- ---------- ----------
```

```
                              201
Trifolium strictum          ---------- ---------- ---------- ---------- ----------
T. glanduliferum            ---------- ---------- ---------- ---------- ----------
T. boissieri                ---------- ---------- ---------- ---------- ----------
T. aureum                   ---------- ---------- ---------- ---------- ----------
T.meduseum                  ---------- ---------- ---------- ---------- ----------
T.subterraneum              ---------- ---------- ---------- ---------- ----------
T.pratense                  ---------- ---------- ---------- ---------- ----------
T.hybridum                  ---------- ---------- ---------- ---------- ----------
T.semipilosum               ---------- ---------- ---------- ---------- ----------
T.occidentale               ---------- ---------- ---------- ---------- ----------
T.repens                    ---------- ---------- ---------- ---------- ----------
T.lupinaster                ---------- ---------- ---------- ---------- ----------
Cicer oxyodon               ---------- ---------- ---------- ---------- ----------
C. chorrassanicum           ---------- ---------- ---------- ---------- ----------
C. arietinum                ---------- ---------- ---------- ---------- ----------
Medicago truncatula_KF      ---------- ---------- ---------- ---------- ----------
M.truncatula_AC             ---------- ---------- ---------- ---------- ----------
M.sativa_K                  ---------- ---------- ---------- ---------- ----------
M.sativa                    ---------- ---------- ---------- ---------- ----------
M.papillosa                 ---------- ---------- ---------- ---------- ----------
M.hybrida                   ---------- ---------- ---------- ---------- ----------
M.falcata                   ---------- ---------- ---------- ---------- ----------
M.sativa_KU                 ---------- ---------- ---------- ---------- ----------
Melilotus albus             ---------- ---------- ---------- ---------- ----------
Hedysarum formosum          ---------- ---------- ---------- ---------- ----------
H.varium                    ---------- ---------- ---------- ---------- ----------
H.singarense                ---------- ---------- ---------- ---------- ----------
H.minjanense                ---------- ---------- ---------- ---------- ----------
Tavrniera glabra            ---------- ---------- ---------- ---------- ----------
T.diffusa                   ---------- ---------- ---------- ---------- ----------
Greuteria membranacea       ---------- ---------- ---------- ---------- ----------
Corethrodendron scoparium   ---------- ---------- ---------- ---------- ----------
Eversmannia subspinosa      ---------- ---------- ---------- ---------- ----------
Onobrychis bungei           ---------- ---------- ---------- ---------- ----------
O.cornuta                   ---------- ---------- ---------- ---------- ----------
O.michauxii                 ---------- ---------- ---------- ---------- ----------
O.subacaulis                ---------- ---------- ---------- ---------- ----------
O.teheranica                ---------- ---------- ---------- ---------- ----------
Alhagi maurorum             ---------- ---------- ---------- ---------- ----------
Caragana microphylla        ---------- ---------- ---------- ---------- ----------
C.korshinski                ---------- ---------- ---------- ---------- ----------
C.kozlowii                  ---------- ---------- ---------- ---------- ----------
C.rosea                     ---------- ---------- ---------- ---------- ----------
Tibetia liangshanensis      ---------- ---------- ---------- ---------- ----------
Gueldenstaedtia verna       ---------- ---------- ---------- ---------- ----------
Halimodendron halodendron   ---------- ---------- ---------- ---------- ----------
Smirnowia turkestana        ---------- ---------- ---------- ---------- ----------
Eremosparton flaccidum      ---------- ---------- ---------- ---------- ----------
Colutea persica             ---------- ---------- ---------- ---------- ----------
C. triphylla                ---------- ---------- ---------- ---------- ----------
Sphaerophysa salsula        ---------- ---------- ---------- ---------- ----------
Podlechiella vogelii        ---------- ---------- ---------- ---------- ----------
Carmichaelia australis      ---------- ---------- ---------- ---------- ----------
Sutherlandia frutescens     ---------- ---------- ---------- ---------- ----------
Astragalus membranaceous    ---------- ---------- ---------- ---------- ----------
A.mongholicus               ---------- ---------- ---------- ---------- ----------
A.nakaianus                 ---------- ---------- ---------- ---------- ----------
A.macropelmatus             ---------- ---------- ---------- ---------- ----------
A.iranicus                  ---------- ---------- ---------- ---------- ----------
A.denudatus                 ---------- ---------- ---------- ---------- ----------
A.odoratus                  ---------- ---------- ---------- ---------- ----------
Oxytropis szovitsii         ---------- ---------- ---------- ---------- ----------
O.iranica                   ---------- ---------- ---------- ---------- ----------
O.kotschyana                ---------- ---------- ---------- ---------- ----------
Erophaca baetica            ---------- ---------- ---------- ---------- ----------
Wisteria floribunda         ---------- ---------- ---------- ---------- ----------
W. sinensis                 ---------- ---------- ---------- ---------- ----------
Glycyrrhiza lepidota        ---------- ---------- ---------- ---------- ----------
G.uralensis                 ---------- ---------- ---------- ---------- ----------
G.glabra                    ---------- ---------- ---------- ---------- ----------
Meristotropis xathioides    ---------- ---------- ---------- ---------- ----------
Robinia pseudoacacia        ---------- ---------- ---------- ---------- ----------
Lotus japonicus             ---------- ---------- ---------- ---------- ----------
Vicia alpestris             ---------- ---------- ---------- ---------- ----------
V.sativa                    ---------- ---------- ---------- ---------- ----------
V.peregrina                 ---------- ---------- ---------- ---------- ----------
V.sepium                    ---------- ---------- ---------- ---------- ----------
V. faba                     ---------- ---------- ---------- ---------- ----------
```

```
V.canescens               ---------- ---------- ---------- ---------- ----------
V.monantha                ---------- ---------- ---------- ---------- ----------
V.tetrasperma             ---------- ---------- ---------- ---------- ----------
V.narbonensis             ---------- ---------- ---------- ---------- ----------
Lens culinaris            ---------- ---------- ---------- ---------- ----------
L.orientalis              ---------- ---------- ---------- ---------- ----------
V.ervilia                 ---------- ---------- ---------- ---------- ----------
Galega officinalis        ---------- ---------- ---------- ---------- ----------
Lathyrus palustris_K      ATGTTGCCTT CACCATCTAT ATCTGCCGGT ACACAGGAGA C----CTTTT
L.palustris_H             ATGTGGCCTT CAGCACCTAT ATGGGCTGGT ACACAGGAGA T----CTTTT
L.davidii                 AGATTGCTTT CAGCACGTAC ATGACCCGGT ACGTTTTGGA C----CTTTT
L.japonicus               ATGTTGCATT TAGCATTTAT ATGACTCAGT ACGCGGGGGA C----CTTTT
L.ochroleucus             AAGTGGCCTT CAGCGTCTAT TTAACCCGGT ACACAGGTAA C----CTTTT
L.littoralis              ACGTAGCCTT CAACATATAT ATTAGCCGGT ACACGTGGGA G----CTTTT
L.venosus                 AGGTTGCCTT CAGCATATAC ATAAGCCAGT ATACAGGGAG C----CTTTT
L.graminifolius           ACGTTGCCTT CAACATTTTT CTCACTCGCC ACATAGGGGT C----CTTTT
L.pubescens               GTGTTGGGTT GAATCTATAC GTGGCGTTCT ATCTCGGTCG TA-TACTTTC
L.sativus_KJ              TTCCTGCATG GAACTTACCT TTTAAGTATA TTAAAAGTAA TAGTTCTTCT
L.sativus_HM              TTCCTGCATG GAACTTACCT TTTAAGTATA TTAAAAGTAA TAGTTCTTCT
L.pseudocicera            ---ATGCATG GAACTTACCT GTTATGGATA ATAAAGACAA TCATTCTTCT
L.chloranthus             ---------- ---------- ---------- ---------- ----------
L.pratensis               ---------- ---------- ---------- ---------- ----------
L.odoratus_HM             ---------- ---------- ---------- ---------- ----------
L.odoratus_kJ             ---------- ---------- ---------- ---------- ----------
L.hirsutus                ---------- ---------- ---------- ---------- ----------
L.annuus                  ---------- ---------- ---------- ---------- ----------
L.cirrhosus               AATTTTTGTT ATTTTTTTGA ACCTTTTTTT GAACGGAGCT TTAATCTTGA
L.latifolius              AATTTTTGTT ATTTTTTTGA CCCTTTTTTG TAACGGAGCT TTAATCTTGA
L.tingitanus              ---------- ---------- ---------- ---------- ----------
Pisum sativum_H           ---------- ---------- ---------- ---------- ----------
Pisum sativum_K           ---------- ---------- ---------- ---------- ----------
Pisum sativum_HG          ---------- ---------- ---------- ---------- ----------
Pisum fulvum              ---------- ---------- ---------- ---------- ----------
Vavilovia formosa         ---------- ---------- ---------- ---------- ----------
L.clymenum                ---------- ---------- ---------- ---------- ----------
L.ochrus                  ---------- ---------- ---------- ---------- ----------
```

```
                            251
Trifolium strictum          ---------- ---------- ---------- ---------- ----------
T. glanduliferum            ---------- ---------- ---------- ---------- ----------
T. boissieri                ---------- ---------- ---------- ---------- ----------
T. aureum                   ---------- ---------- ---------- ---------- ----------
T.meduseum                  ---------- ---------- ---------- ---------- ----------
T.subterraneum              ---------- ---------- ---------- ---------- ----------
T.pratense                  ---------- ---------- ---------- ---------- ----------
T.hybridum                  ---------- ---------- ---------- ---------- ----------
T.semipilosum               ---------- ---------- ---------- ---------- ----------
T.occidentale               ---------- ---------- ---------- ---------- ----------
T.repens                    ---------- ---------- ---------- ---------- ----------
T.lupinaster                ---------- ---------- ---------- ---------- ----------
Cicer oxyodon               ---------- ---------- ---------- ---------- ----------
C. chorrassanicum           ---------- ---------- ---------- ---------- ----------
C. arietinum                ---------- ---------- ---------- ---------- ----------
Medicago truncatula_KF      ---------- ---------- ---------- ---------- ----------
M.truncatula_AC             ---------- ---------- ---------- ---------- ----------
M.sativa_K                  ---------- ---------- ---------- ---------- ----------
M.sativa                    ---------- ---------- ---------- ---------- ----------
M.papillosa                 ---------- ---------- ---------- ---------- ----------
M.hybrida                   ---------- ---------- ---------- ---------- ----------
M.falcata                   ---------- ---------- ---------- ---------- ----------
M.sativa_KU                 ---------- ---------- ---------- ---------- ----------
Melilotus albus             ---------- ---------- ---------- ---------- ----------
Hedysarum formosum          ---------- ---------- ---------- ---------- ----------
H.varium                    ---------- ---------- ---------- ---------- ----------
H.singarense                ---------- ---------- ---------- ---------- ----------
H.minjanense                ---------- ---------- ---------- ---------- ----------
Tavrniera glabra            ---------- ---------- ---------- ---------- ----------
T.diffusa                   ---------- ---------- ---------- ---------- ----------
Greuteria membranacea       ---------- ---------- ---------- ---------- ----------
Corethrodendron scoparium   ---------- ---------- ---------- ---------- ----------
Eversmannia subspinosa      ---------- ---------- ---------- ---------- ----------
Onobrychis bungei           ---------- ---------- ---------- ---------- ----------
O.cornuta                   ---------- ---------- ---------- ---------- ----------
O.michauxii                 ---------- ---------- ---------- ---------- ----------
O.subacaulis                ---------- ---------- ---------- ---------- ----------
O.teheranica                ---------- ---------- ---------- ---------- ----------
Alhagi maurorum             ---------- ---------- ---------- ---------- ----------
Caragana microphylla        ---------- ---------- ---------- ---------- ----------
C.korshinski                ---------- ---------- ---------- ---------- ----------
C.kozlowii                  ---------- ---------- ---------- ---------- ----------
C.rosea                     ---------- ---------- ---------- ---------- ----------
Tibetia liangshanensis      ---------- ---------- ---------- ---------- ----------
Gueldenstaedtia verna       ---------- ---------- ---------- ---------- ----------
Halimodendron halodendron   ---------- ---------- ---------- ---------- ----------
Smirnowia turkestana        ---------- ---------- ---------- ---------- ----------
Eremosparton flaccidum      ---------- ---------- ---------- ---------- ----------
Colutea persica             ---------- ---------- ---------- ---------- ----------
C. triphylla                ---------- ---------- ---------- ---------- ----------
Sphaerophysa salsula        ---------- ---------- ---------- ---------- ----------
Podlechiella vogelii        ---------- ---------- ---------- ---------- ----------
Carmichaelia australis      ---------- ---------- ---------- ---------- ----------
Sutherlandia frutescens     ---------- ---------- ---------- ---------- ----------
Astragalus membranaceous    ---------- ---------- ---------- ---------- ----------
A.mongholicus               ---------- ---------- ---------- ---------- ----------
A.nakaianus                 ---------- ---------- ---------- ---------- ----------
A.macropelmatus             ---------- ---------- ---------- ---------- ----------
A.iranicus                  ---------- ---------- ---------- ---------- ----------
A.denudatus                 ---------- ---------- ---------- ---------- ----------
A.odoratus                  ---------- ---------- ---------- ---------- ----------
Oxytropis szovitsii         ---------- ---------- ---------- ---------- ----------
O.iranica                   ---------- ---------- ---------- ---------- ----------
O.kotschyana                ---------- ---------- ---------- ---------- ----------
Erophaca baetica            ---------- ---------- ---------- ---------- ----------
Wisteria floribunda         ---------- ---------- ---------- ---------- ----------
W. sinensis                 ---------- ---------- ---------- ---------- ----------
Glycyrrhiza lepidota        ---------- ---------- ---------- ---------- ----------
G.uralensis                 ---------- ---------- ---------- ---------- ----------
G.glabra                    ---------- ---------- ---------- ---------- ----------
Meristotropis xathioides    ---------- ---------- ---------- ---------- ----------
Robinia pseudoacacia        ---------- ---------- ---------- ---------- ----------
Lotus japonicus             ---------- ---------- ---------- ---------- ----------
Vicia alpestris             ---------- ---------- ---------- ---------- ----------
V.sativa                    ---------- ---------- ---------- ---------- ----------
V.peregrina                 ---------- ---------- ---------- ---------- ----------
V.sepium                    ---------- ---------- ---------- ---------- ----------
V. faba                     ---------- ---------- ---------- ---------- ----------
```

V.canescens	----------	----------	----------	----------	----------
V.monantha	----------	----------	----------	----------	----------
V.tetrasperma	----------	----------	----------	----------	----------
V.narbonensis	----------	----------	----------	----------	----------
Lens culinaris	----------	----------	----------	----------	----------
L.orientalis	----------	----------	----------	----------	----------
V.ervilia	----------	----------	----------	----------	----------
Galega officinalis	----------	----------	----------	----------	----------
Lathyrus palustris_K	A-------AG	AAAGGTCAAT	CTTGATCTAA	C--AGATAAA	CTCGCCTTTA
L.palustris_H	A-------AG	AAGTGTAAAC	CTTGATCTAA	A--AGATAAA	CTAGCTGTTA
L.davidii	C-------AG	ACAATTAAAT	CTTGATGTAA	G--AAATAAG	CTAGCCCTTC
L.japonicus	A-------AG	AAAATTGAAC	CTTGATCTAA	G--AGATAAG	CTCGCCTTTA
L.ochroleucus	A-------AG	AAAATTAAAT	CTGGATCTAA	G--TGATAAG	CTGGCCTTTG
L.littoralis	A-------AG	AAAATTAAAC	CTAGATCTAA	C--AGATAAG	CTCGCCTTTA
L.venosus	A-------AG	AAAATTAAAT	ATGGACCTAA	G--TGATAAG	CTGGCCTTTG
L.graminifolius	A-------AA	AAAATTAAAC	CTGGATTTAA	G--CGATAAG	CTGGCCTTTG
L.pubescens	GCGGCGCTAT	GGAATCCAAC	CTGTTCTTAA	CCTTATCGAC	CCACCCTTTC
L.sativus_KJ	CCTAGAAAGA	AGCCGCCCGT	ATCCGCCACA	CATCCTGGTG	GATTTATTTA
L.sativus_HM	CCTAGAAAGA	AGCCGCCCGT	ATCCGCCACA	CATCCTGGTG	GATTTATTTA
L.pseudocicera	CGTGCACAAA	AGCC---CAT	CTAAGACAAA	CATTGTGTTG	GATTTCTTAA
L.chloranthus	----------	----------	----------	----------	----------
L.pratensis	----------	----------	----------	----------	----------
L.odoratus_HM	----------	----------	----------	----------	----------
L.odoratus_kJ	----------	----------	----------	----------	----------
L.hirsutus	----------	----------	----------	----------	----------
L.annuus	----------	----------	----------	----------	----------
L.cirrhosus	TGATCATAAA	AGTGATCAAT	ATGAGCCTTG	CAATAACCCA	AGTTACGCAA
L.latifolius	TGATCATAAA	AATGATCAAT	ATAGGCCTTG	CAATAACCCA	AGTTACGCAA
L.tingitanus	----------	----------	----------	----------	----------
Pisum sativum_H	----------	----------	----------	----------	----------
Pisum sativum_K	----------	----------	----------	----------	----------
Pisum sativum_HG	----------	----------	----------	----------	----------
Pisum fulvum	----------	----------	----------	----------	----------
Vavilovia formosa	----------	----------	----------	----------	----------
L.clymenum	----------	----------	----------	----------	----------
L.ochrus	----------	----------	----------	ATGAAGAATA	GCATGGAAAA

```
                             301
Trifolium strictum          ---------- ---------- ---------- ---------- ----------
T. glanduliferum            ---------- ---------- ---------- ---------- ----------
T. boissieri                ---------- ---------- ---------- ---------- ----------
T. aureum                   ---------- ---------- ---------- ---------- ----------
T.meduseum                  ---------- ---------- ---------- ---------- ----------
T.subterraneum              ---------- ---------- ---------- ---------- ----------
T.pratense                  ---------- ---------- ---------- ---------- ----------
T.hybridum                  ---------- ---------- ---------- ---------- ----------
T.semipilosum               ---------- ---------- ---------- ---------- ----------
T.occidentale               ---------- ---------- ---------- ---------- ----------
T.repens                    ---------- ---------- ---------- ---------- ----------
T.lupinaster                ---------- ---------- ---------- ---------- ----------
Cicer oxyodon               ---------- ---------- ---------- ---------- ----------
C. chorrassanicum           ---------- ---------- ---------- ---------- ----------
C. arietinum                ---------- ---------- ---------- ---------- ----------
Medicago truncatula_KF      ---------- ---------- ---------- ---------- ----------
M.truncatula_AC             ---------- ---------- ---------- ---------- ----------
M.sativa_K                  ---------- ---------- ---------- ---------- ----------
M.sativa                    ---------- ---------- ---------- ---------- ----------
M.papillosa                 ---------- ---------- ---------- ---------- ----------
M.hybrida                   ---------- ---------- ---------- ---------- ----------
M.falcata                   ---------- ---------- ---------- ---------- ----------
M.sativa_KU                 ---------- ---------- ---------- ---------- ----------
Melilotus albus             ---------- ---------- ---------- ---------- ----------
Hedysarum formosum          ---------- ---------- ---------- ---------- ----------
H.varium                    ---------- ---------- ---------- ---------- ----------
H.singarense                ---------- ---------- ---------- ---------- ----------
H.minjanense                ---------- ---------- ---------- ---------- ----------
Tavrniera glabra            ---------- ---------- ---------- ---------- ----------
T.diffusa                   ---------- ---------- ---------- ---------- ----------
Greuteria membranacea       ---------- ---------- ---------- ---------- ----------
Corethrodendron scoparium   ---------- ---------- ---------- ---------- ----------
Eversmannia subspinosa      ---------- ---------- ---------- ---------- ----------
Onobrychis bungei           ---------- ---------- ---------- ---------- ----------
O.cornuta                   ---------- ---------- ---------- ---------- ----------
O.michauxii                 ---------- ---------- ---------- ---------- ----------
O.subacaulis                ---------- ---------- ---------- ---------- ----------
O.teheranica                ---------- ---------- ---------- ---------- ----------
Alhagi maurorum             ---------- ---------- ---------- ---------- ----------
Caragana microphylla        ---------- ---------- ---------- ---------- ----------
C.korshinski                ---------- ---------- ---------- ---------- ----------
C.kozlowii                  ---------- ---------- ---------- ---------- ----------
C.rosea                     ---------- ---------- ---------- ---------- ----------
Tibetia liangshanensis      ---------- ---------- ---------- ---------- ----------
Gueldenstaedtia verna       ---------- ---------- ---------- ---------- ----------
Halimodendron halodendron   ---------- ---------- ---------- ---------- ----------
Smirnowia turkestana        ---------- ---------- ---------- ---------- ----------
Eremosparton flaccidum      ---------- ---------- ---------- ---------- ----------
Colutea persica             ---------- ---------- ---------- ---------- ----------
C. triphylla                ---------- ---------- ---------- ---------- ----------
Sphaerophysa salsula        ---------- ---------- ---------- ---------- ----------
Podlechiella vogelii        ---------- ---------- ---------- ---------- ----------
Carmichaelia australis      ---------- ---------- ---------- ---------- ----------
Sutherlandia frutescens     ---------- ---------- ---------- ---------- ----------
Astragalus membranaceous    ---------- ---------- ---------- ---------- ----------
A.mongholicus               ---------- ---------- ---------- ---------- ----------
A.nakaianus                 ---------- ---------- ---------- ---------- ----------
A.macropelmatus             ---------- ---------- ---------- ---------- ----------
A.iranicus                  ---------- ---------- ---------- ---------- ----------
A.denudatus                 ---------- ---------- ---------- ---------- ----------
A.odoratus                  ---------- ---------- ---------- ---------- ----------
Oxytropis szovitsii         ---------- ---------- ---------- ---------- ----------
O.iranica                   ---------- ---------- ---------- ---------- ----------
O.kotschyana                ---------- ---------- ---------- ---------- ----------
Erophaca baetica            ---------- ---------- ---------- ---------- ----------
Wisteria floribunda         ---------- ---------- ---------- ---------- ----------
W. sinensis                 ---------- ---------- ---------- ---------- ----------
Glycyrrhiza lepidota        ---------- ---------- ---------- ---------- ----------
G.uralensis                 ---------- ---------- ---------- ---------- ----------
G.glabra                    ---------- ---------- ---------- ---------- ----------
Meristotropis xathioides    ---------- ---------- ---------- ---------- ----------
Robinia pseudoacacia        ---------- ---------- ---------- ---------- ----------
Lotus japonicus             ---------- ---------- ---------- ---------- ----------
Vicia alpestris             ---------- ---------- ---------- ---------- ----------
V.sativa                    ---------- ---------- ---------- ---------- ----------
V.peregrina                 ---------- ---------- ---------- ---------- ----------
V.sepium                    ---------- ---------- ---------- ---------- ----------
V. faba                     ---------- ---------- ---------- ---------- ----------
```

V.canescens	----------	----------	----------	----------	----------
V.monantha	----------	----------	----------	----------	----------
V.tetrasperma	----------	----------	----------	----------	----------
V.narbonensis	----------	----------	----------	----------	----------
Lens culinaris	----------	----------	----------	----------	----------
L.orientalis	----------	----------	----------	----------	----------
V.ervilia	----------	----------	----------	----------	----------
Galega officinalis	----------	----------	----------	----------	----------
Lathyrus palustris_K	TTACAGCCAC	AGTAGAATTC	TTTGTGATTA	TACTTCGCAT	TGTT------
L.palustris_H	TTACAGCCAC	AGTAGAATTC	TTTGTCATTA	TACTTCGAAT	TGTT------
L.davidii	TTACCGCAGT	AATAGAATTC	GGAGTAATTA	TACTGCGAAT	TGTT------
L.japonicus	TTACGGCTAT	ATTTGAATTC	TTTGTCATTA	TACTGCGCAT	TGTT------
L.ochroleucus	TTACGGCGAT	ATTAGACTTC	ACAGTAATAA	TACTGCGCAT	TCTT------
L.littoralis	TTACGGCTAT	ATTTGAATTC	TTTGTCATTA	TACTGCGCAT	TGTT------
L.venosus	TTACGGCGAT	AGTAGAATTC	TTTGTGATTA	TCTTGCGAAT	AGTT------
L.graminifolius	TTGTAGCTAT	CGTCGATTTC	TTTGTAATCA	TACTGCGCAT	CGTT------
L.pubescens	TCATGGAACA	AGTGGAAATC	TTGGGAAGTG	TGGTGAAAAT	GCTTAGTTTG
L.sativus_KJ	TTTTATGTTT	TGCGGCCTTT	CTTTTTAGGA	ATATCTTTTT	CGTTTTGTTT
L.sativus_HM	TTTTATGTTT	TGCGGCCTTT	CTTTTTAGGA	ATATCTTTTT	CGTTTTGTTT
L.pseudocicera	TGTTCTGTTT	TGGGGTGTTC	CTGTTTAGGA	ACATAGTGTT	CGTTTTATTT
L.chloranthus	----------	----------	----------	----------	----------
L.pratensis	----------	----------	----------	----------	----------
L.odoratus_HM	----------	----------	----------	----------	----------
L.odoratus_kJ	----------	----------	----------	----------	----------
L.hirsutus	----------	----------	----------	----------	----------
L.annuus	----------	----------	----------	----------	----------
L.cirrhosus	GATAAC--TT	GGTCTCGTTT	TTGATTGTCC	TTTTTCTCTT	TTTGTTTCAA
L.latifolius	GATAAC--TT	GGTCTCGTTT	TTGATTGGCC	TTTTTCTCTT	CTTGTTTCAA
L.tingitanus	----------	----------	----------	----------	----------
Pisum sativum_H	----------	----------	----------	----------	----------
Pisum sativum_K	----------	----------	----------	----------	----------
Pisum sativum_HG	----------	----------	----------	----------	----------
Pisum fulvum	----------	----------	----------	----------	----------
Vavilovia formosa	----------	----------	----------	----------	----------
L.clymenum	----------	----------	----------	----------	----------
L.ochrus	ACGGACTCTG	ACTTTGCTGA	ATTTTTATCG	GGTTAATAAG	AAGGCTATTT

```
                             351
Trifolium strictum          ---------- ---------- -ATGTCTGCT TTATTGGTTC ATGTTCCAAA
T. glanduliferum            ---------- ---------- -ATGTCTGCT TTATTGGTTC ATGTTCCAAA
T. boissieri                ---------- ---------- -ATGTCTGCC TTATTGGTTC ATGTTCCAAA
T. aureum                   ---------- ---------- -ATGTCTGCT TTATTGGTTC ATGTTCCAAA
T.meduseum                  ---------- ---------- -ATGTCTGCT TTATTGGTTC ATGTTCCCAA
T.subterraneum              ---------- ---------- -ATGTCTGCT TTATTGGTTC ATGTTCCCAA
T.pratense                  ---------- ---------- -ATGTCTGCT TTATTGGTTC ATGTTCCAAA
T.hybridum                  ---------- ---------- -ATGTCTGCT TTATTGGTTC ATGTTCCAAA
T.semipilosum               ---------- ---------- -ATGTCTGCT TTATTGGTTC ATGTTCCAAA
T.occidentale               ---------- ---------- -ATGTCTGCT TTATTAGTTC ATGTTCCAAA
T.repens                    ---------- ---------- -ATGTCTGCT TTATTAGTTC ATGTTCCAAA
T.lupinaster                ---------- ---------- -ATGTCTGCT TTATTGGTTC ATGTTCCAAA
Cicer oxyodon               ---------- ---------- -ATGTCTGCT TTATTCGTTT ATGTTCCAAA
C. chorrassanicum           ---------- ---------- -ATGTCTGTT TTATTCGTTT ATGTTCCAAA
C. arietinum                ---------- ---------- -ATGTCTGCT TTATTCGTTT ATGTTCCAAA
Medicago truncatula_KF      ---------- ---------- -ATGTCTGCT TTATTGGTTA ATGTTCCAAA
M.truncatula_AC             ---------- ---------- -ATGTCTGCT TTATTGGTTA ATGTTCCAAA
M.sativa_K                  ---------- ---------- -ATGTCTGCT TTATTGGTTA ATGTTCCAAA
M.sativa                    ---------- ---------- -ATGTCTGCT TTATTGGTTA ATGTTCCAAA
M.papillosa                 ---------- ---------- -ATGTCTGCT TTATTGGTTA ATGTTCCAAA
M.hybrida                   ---------- ---------- -ATGTCTGCT TTATTGGTTA ATGTTCCAAA
M.falcata                   ---------- ---------- -ATGTCTGCT TTATTGGTTA ATGTTCCAAA
M.sativa_KU                 ---------- ---------- -ATGTCTGCT TTATTGGTTA ATGTTCCAAA
Melilotus albus             ---------- ---------- -ATGTCTGCT TTATTGGTTA ATGTTCCAAA
Hedysarum formosum          ---------- ---------- ---------- ---------- ----------
H.varium                    ---------- ---------- ---------- ---------- ----------
H.singarense                ---------- ---------- ---------- ---------- ----------
H.minjanense                ---------- ---------- ---------- ---------- ----------
Tavrniera glabra            ---------- ---------- ---------- ---------- ----------
T.diffusa                   ---------- ---------- ---------- ---------- ----------
Greuteria membranacea       ---------- ---------- ---------- ---------- ----------
Corethrodendron scoparium   ---------- ---------- ---------- ---------- ----------
Eversmannia subspinosa      ---------- ---------- ---------- ---------- ----------
Onobrychis bungei           ---------- ---------- ---------- ---------- ----------
O.cornuta                   ---------- ---------- ---------- ---------- ----------
O.michauxii                 ---------- ---------- ---------- ---------- ----------
O.subacaulis                ---------- ---------- ---------- ---------- ----------
O.teheranica                ---------- ---------- ---------- ---------- ----------
Alhagi maurorum             ---------- ---------- ---------- ---------- ----------
Caragana microphylla        ---------- ---------- ---------- ---------- ----------
C.korshinski                ---------- ---------- ---------- ---------- ----------
C.kozlowii                  ---------- ---------- ---------- ---------- ----------
C.rosea                     ---------- ---------- ---------- ---------- ----------
Tibetia liangshanensis      ---------- ---------- ---------- ---------- ----------
Gueldenstaedtia verna       ---------- ---------- ---------- ---------- ----------
Halimodendron halodendron   ---------- ---------- ---------- ---------- ----------
Smirnowia turkestana        ---------- ---------- ---------- ---------- ----------
Eremosparton flaccidum      ---------- ---------- ---------- ---------- ----------
Colutea persica             ---------- ---------- ---------- ---------- ----------
C. triphylla                ---------- ---------- ---------- ---------- ----------
Sphaerophysa salsula        ---------- ---------- ---------- ---------- ----------
Podlechiella vogelii        ---------- ---------- ---------- ---------- ----------
Carmichaelia australis      ---------- ---------- ---------- ---------- ----------
Sutherlandia frutescens     ---------- ---------- ---------- ---------- ----------
Astragalus membranaceous    ---------- ---------- ---------- ---------- ----------
A.mongholicus               ---------- ---------- ---------- ---------- ----------
A.nakaianus                 ---------- ---------- ---------- ---------- ----------
A.macropelmatus             ---------- ---------- ---------- ---------- ----------
A.iranicus                  ---------- ---------- ---------- ---------- ----------
A.denudatus                 ---------- ---------- ---------- ---------- ----------
A.odoratus                  ---------- ---------- ---------- ---------- ----------
Oxytropis szovitsii         ---------- ---------- ---------- ---------- ----------
O.iranica                   ---------- ---------- ---------- ---------- ----------
O.kotschyana                ---------- ---------- ---------- ---------- ----------
Erophaca baetica            ---------- ---------- ---------- ---------- ----------
Wisteria floribunda         ---------- ---------- ---------- ---------- ----------
W. sinensis                 ---------- ---------- ---------- ---------- ----------
Glycyrrhiza lepidota        ---------- ---------- ---------- ---------- ----------
G.uralensis                 ---------- ---------- ---------- ---------- ----------
G.glabra                    ---------- ---------- ---------- ---------- ----------
Meristotropis xathioides    ---------- ---------- ---------- ---------- ----------
Robinia pseudoacacia        ---------- ---------- ---------- ---------- ----------
Lotus japonicus             ---------- ---------- ---------- ---------- ----------
Vicia alpestris             ---------- ---------- -ATGTCTGCT TTATTGGTTC CTGTTCCAAA
V.sativa                    ---------- ---------- -ATGTCTGCT TTATTGGTTC ATGTTACAAA
V.peregrina                 ---------- ---------- -ATGTCTGCT TTATTGGTTC ATGTTACAAA
V.sepium                    ---------- ---------- -ATGTCTGCT TTATTGGTTC ATGTTACAAA
V. faba                     ---------- ---------- -ATGTCTGCT TTATTGGTTC ATGTTCCAAA
```

```
V.canescens               ---------- ---------- -ATGTCTGCT TTATTGGTTC ATGTTCCAAA
V.monantha                ---------- ---------- -ATGTCTGCT TTATTGGTTC ATGTTCCAAA
V.tetrasperma             ---------- ---------- -ATGTCTGGT TTATTGGTTC ATGTTCCAAA
V.narbonensis             ---------- ---------- -ATGTCTGCT TTATTGGTTC ATGTTCCAAA
Lens culinaris            ---------- ---------- -ATGTCTGCT TTCTTGGTTC ATGTTCCAAA
L.orientalis              ---------- ---------- -ATGTCTGCT TTCTTGGTTC AAGTTCCAAA
V.ervilia                 ---------- ---------- -ATGTCTGCT TTATTGGTTC CTGTTCCAAA
Galega officinalis        ---------- ---------- -ATGTCGACT TTATTGGTGA ATGTTCCTAA
Lathyrus palustris_K      ------CTAG TGTGGCTTTT TTTATATTAT TTGATAGAAA GAACTTTTCG
L.palustris_H             ------CTAG TGTGGCTTTT TTTATATTAT TTGATAGAAA GAACTATTCG
L.davidii                 ------CTAG TGTGGCTTTT TTTCTATTTT TTGATGGAAA AGACATTTCG
L.japonicus               ------CTAG TGTGGCTTTT TGTATATTAT TTGATAGAAA CAACTTTTCG
L.ochroleucus             ------CTAG TGTGGCTTTT TTTCTATTAT TTGATAGAAC GGACTGTTCG
L.littoralis              ------CTAG TGTGGCTTTT TTTATATTAT TTGATCGAAA GAACCATTCG
L.venosus                 ------CTGG TGTGGCTTTT TTTATATTAT TTGATAGAAA GAACTATTCG
L.graminifolius           ------CTGG TGTGGCTTTT TCTATATTAT TTGATAGAAA GAACTTTTCG
L.pubescens               ATCCCCCTTG GATTGCTTCT TTTCTTGAAT AGTGTAGGAA AATCCATTTG
L.sativus_KJ              TTTTATTTCC TTAGAACAAT TTTGGATTAT TTACTTCAAA TGATCCGTGA
L.sativus_HM              TTTTATTTCC TTAGAACAAT TTTGGATTAT TTACTTCAAA TGATCCGTGA
L.pseudocicera            TTTTATTTCG TTAGAACAAT TTTTAATTAT TTACTTCAAA TCGTCCATGA
L.chloranthus             ---------- ---------- ---------- ---------- ----------
L.pratensis               ---------- ---------- ---------- ---------- ----------
L.odoratus_HM             ---------- ---------- ---------- ---------- ----------
L.odoratus_kJ             ---------- ---------- ---------- ---------- ----------
L.hirsutus                ---------- ---------- ---------- ---------- ----------
L.annuus                  ---------- ---------- ---------- ---------- ----------
L.cirrhosus               AGTCAATTTT ATAAGCGTGT TGTACCCTAT TTATTTCAAA CCGGCATTAA
L.latifolius              ATTCAATTTG GAAGGCGTGT TGTATCCTAT TTATTTCAAA CCGGCATTAA
L.tingitanus              ---------- ---------- ---------- ---------- ----------
Pisum sativum_H           ---------- ---------- ---------- ---------- ----------
Pisum sativum_K           ---------- ---------- ---------- ---------- ----------
Pisum sativum_HG          ---------- ---------- ---------- ---------- ----------
Pisum fulvum              ---------- ---------- ---------- ---------- ----------
Vavilovia formosa         ---------- ---------- ---------- ---------- ----------
L.clymenum                ---------- ---------- ---------- ---------- ----------
L.ochrus                  ATATGGATAT AAATGTGCCG GTTATAAAGG TGAATATACT ATTAGGAGCT
```

```
                              401
Trifolium strictum          ATTAATTAGT TGGGGATCAG AAAAACATGG TTGTC----- ----GTTCAC
T. glanduliferum            ATTCATTAGT TGGGGATCAG AAAAACATGG TTGTC----- ----GTTCAC
T. boissieri                ATTCATTAGT GGGGGATCAG AAAAACATGG TTGTC----- ----GTTCAC
T. aureum                   ATTCATTAGT GGGGGATCAG AAAAACATGG TTGTC----- ----GTTCAC
T.meduseum                  ATTCATTAGT TGGGAAGCAG AAAAACATGG TTGTC----- ----GTTCAC
T.subterraneum              ATTCATTAGT TGGGAAGCAG AAAAACATGG TTGTC----- ----GTTCAC
T.pratense                  ATTAATTAGT TGGGGATCAG AAAAACATGG TTGTC----- ----GTTCAC
T.hybridum                  ATTCATTAGT TGGGGATCAA AAAAACATGG TTGTC----- ----GTTCAC
T.semipilosum               ATTCATTAGT TGGGGATCAA AAAAACATGG TTGTC----- ----GTTCAC
T.occidentale               ATTCATTAGT TGGGGATCGA AAAAACAGGG TTGTC----- ----GTTCAC
T.repens                    ATTCATTAGT TGGAGATCGA AAAAACAGGG TTGTC----- ----GTTCAC
T.lupinaster                ATTCATTAGT TGGGGATCAG AAAAACATGG TTGTC----- ----GTTCAC
Cicer oxyodon               ATTCATT--- TTGGGGTCAG AAAAATATGT TTGTC----- ----GTTCAC
C. chorrassanicum           ATTCATT--- TTGGGGTCAG AAAAATATGT TTGTC----- ----GTTCAC
C. arietinum                ATTCATT--- TTGGGGTCAG AAAAATATGT TTGTC----- ----GCTCAC
Medicago truncatula_KF      ATTAATTAGT TGGGGATCAG AAAAATATGG TTGTC----- ----GTTCAC
M.truncatula_AC             ATTTATTAGT TGGGGATCAG AAAAACATGG TTGTC----- ----GTTCAC
M.sativa_K                  ATTCATTAGT TGGGGATCAG AAAAACATGG TTGTC----- ----GTTCAC
M.sativa                    ATTCATTAGT TGGGGATCAG AAAAACATGG TTGTC----- ----GTTCAC
M.papillosa                 ATTCATTAGT TGGGGATCAG AAAAACATGG TTGTC----- ----GTTCAC
M.hybrida                   ATTCATTAGT TGGGGATCAG AAAAACATGG TTGTC----- ----GTTCAC
M.falcata                   ATTCATTAGT TGGGGATCAG AAAAACATGG TTGTC----- ----GTTCAC
M.sativa_KU                 ATTCATTAGT TGGGGATCAG AAAAACATGG TTGTC----- ----GTTCAC
Melilotus albus             ATTCGTTAGT TGGGGATCAG AAAAACATGG TTGTC----- ----GTTCAC
Hedysarum formosum          ---------- ---------- --ATGCGTAG CTTCCAA--- ---GGACAAG
H.varium                    ---------- ---------- --ATGCGTAG CTTCCAA--- ---GGACAAG
H.singarense                ---------- ---------- --ATGCGTAG CTTCCAA--- ---GGACAAG
H.minjanense                ---------- ---------- --ATGCGTAG CTTCCAA--- ---GGACAAG
Tavrniera glabra            ---------- ---------- --ATGCGTAG CTTCCAA--- ---GGACAAG
T.diffusa                   ---------- ---------- --ATGCGTAG CTTCCAA--- ---GGACAAG
Greuteria membranacea       ---------- ---------- --ATGCGTAG CTTCCAA--- ---GGACAAG
Corethrodendron scoparium   ---------- ---------- --ATGCGTAG CTTCCAA--- ---GGACAAG
Eversmannia subspinosa      ---------- ---------- --ATGCGTAG CTTCCAA--- ---GGACAAG
Onobrychis bungei           ---------- ---------- --ATGCGTAG CTTCCAA--- ---GGACAAG
O.cornuta                   ---------- ---------- --ATGCGTAG CTTCCAA--- ---GGACAAG
O.michauxii                 ---------- ---------- --ATGCGTAG CTTCCAA--- ---GGACAAG
O.subacaulis                ---------- ---------- --ATGCGTAG CTTCCAA--- ---GGACAAG
O.teheranica                ---------- ---------- --ATGCGTAG CTTCCAA--- ---GAACAAG
Alhagi maurorum             ---------- ---------- --ATGCGTCG CTTCCAA--- ---GAACAAG
Caragana microphylla        ---------- ---------- --ATGCGTCG CTACCAA--- ---GAACAAG
C.korshinski                ---------- ---------- --ATGCGTCG CTACCAA--- ---GAACAAG
C.kozlowii                  ---------- ---------- --ATGCGTCG CTACCAA--- ---GAACAAG
C.rosea                     ---------- ---------- --ATGCGTCG CTACCAA--- ---GAACAAG
Tibetia liangshanensis      ---------- ---------- --ATGCGTCG CTTC------ ------CAAG
Gueldenstaedtia verna       ---------- ---------- --ATGCGTCG CTTC------ ------CAAA
Halimodendron halodendron   ---------- ---------- --ATGTCTCG ATTCCAA--- ---GATCTCA
Smirnowia turkestana        ---------- ---------- --ATGCGTCG CTTC------ ------GCAG
Eremosparton flaccidum      ---------- ---------- --ATGCGTCG CTTC------ ------GCAG
Colutea persica             ---------- ---------- --ATGCGTCG CTTC------ ------GCAG
C. triphylla                ---------- ---------- --ATGCGTCG CTTC------ ------GCAG
Sphaerophysa salsula        ---------- ---------- --ATGCGTCG CTTC------ ------GCAG
Podlechiella vogelii        ---------- ---------- --ATGCGTCG CTTC------ ------GCAG
Carmichaelia australis      ---------- ---------- --ATGCGTCG CTTC------ ------GCAG
Sutherlandia frutescens     ---------- ---------- --ATGCGTCG CTTC------ ------GCAG
Astragalus membranaceous    ---------- ---------- --ATGCGTCG CTTC------ ------GCAG
A.mongholicus               ---------- ---------- --ATGCGTCG CTTC------ ------GCAG
A.nakaianus                 ---------- ---------- --ATGCGTCG CTTC------ ------GCAG
A.macropelmatus             ---------- ---------- --ATGCGTCG CTTC------ ------GCAG
A.iranicus                  ---------- ---------- --ATGCGTCG CTTC------ ------GCAG
A.denudatus                 ---------- ---------- --ATGCGTCG CTTC------ ------GCAG
A.odoratus                  ---------- ---------- --ATGCGTCG CTTC------ ------GCAG
Oxytropis szovitsii         ---------- ---------- --ATGCGTCG CTTC------ ------GCAG
O.iranica                   ---------- ---------- --ATGCGTCG CTTC------ ------GCAG
O.kotschyana                ---------- ---------- --ATGCGTCG CTTC------ ------GCAG
Erophaca baetica            ---------- ---------- --ATGCGTCG CTTC------ ------GCAG
Wisteria floribunda         ---ATGATTG GGGAGTCAAA ACATGCTTGG CGCT------ -------CAG
W. sinensis                 ---ATGATTG GGGAGTCAAA ACATGCTTGG CGCT------ -------CAG
Glycyrrhiza lepidota        ---ATGACTT GGGAGTCAGA ACATGTTTGG CGCT------ -------CAG
G.uralensis                 ---ATGACTT GGGAGTCAGA ACATGTTTGG CGCT------ -------CAG
G.glabra                    ---ATGACTT GGGAGTCAGA ACATGTTTGG CGCT------ -------CAG
Meristotropis xathioides    ---ATGACTT GGGAGTCAGA ACATGTTTGG CGCT------ -------CAG
Robinia pseudoacacia        ---ATGTTTT GGATCTCAAA AGATACATGC CACA------ -------CAG
Lotus japonicus             ---ATGACTT GGATATCAGA AGATACGTGG CAGT------ -------CAG
Vicia alpestris             ATTCATTAGT TGGGGGTCAG AAAAACATAG TTGTC----- ----GTTCAC
V.sativa                    ATTCAGTAGT TGGGGGTCAG AAAAACTTGG TTGTC----- ----GTTCAC
V.peregrina                 ATTCAGTAGT TGGGGGTCAG AAAAACTTGG TTGTC----- ----GTTCAC
V.sepium                    ATTCAGTAGT TGGGGGTCAG AAAAACTTGG TTGTC----- ----GTTCAC
V. faba                     ATTCATTAGT TGGGGGTCAG AAAAACTTGG TTGTC----- ----GTTCAC
```

```
V.canescens                 ATTCATTAGT TGGGGGTCAG AAAAACATGG TTGTC----- ----GTTCAC
V.monantha                  ATTCATTAGT TGGGGGTCAG AAAAACATGG TTGTC----- ----GTTCAC
V.tetrasperma               ATTCATTAGT TGGGGGTCAG AAAAACATGG TTGTC----- ----GTTCAC
V.narbonensis               ATTCATTAGT TGGGGGTCAA AAAAACATGG TTGTC----- ----GTTCAC
Lens culinaris              ATT------- --GGGGTCAG AAAAACAAGG TTGTC----- ----GTTCAC
L.orientalis                ATT------- --GGGGTCAG AAAAACAAGG TTTTC----- ----GTTCAC
V.ervilia                   ATTCATTAGT TGGGGGTCAG AAAAACATAG TTGTC----- ----GTTCAC
Galega officinalis          ATTCATTAGT TGGGGGTCAG AAAAACACGG TTGTC----- ----GTTCAC
Lathyrus palustris_K        ------CAAG AGGAGGGCAA AAAAACACGG TTTTTGTTCC TATCTCTCAA
L.palustris_H               ------GAAT AGGAGGGCAA AAAAACACGG TTTTTGTTCC TATCTCTCAA
L.davidii                   ------CAAT AGGCGTGCAA AAAAACACGG TTTTTGTTCG TATTTATCAA
L.japonicus                 ------CAAG AGGAGGGCAA AAAAACACGG TTTTTGTTCC TATCTGTCAA
L.ochroleucus               ------TAAG AGGAGGGCAA AAAAACACGG TTTTTGTTCA TATAGGTCAA
L.littoralis                ------CAAG AGGAGGGAAA AAAAACACGG TTTTTGTTCC TATCTATCAA
L.venosus                   ------CAAG CGGAGGGCAA AAAAACACGG TTTTTGTTCA CACATGTCAA
L.graminifolius             ------GAAG AGGAGGGCAA AAAAACACGG TTTTTGTTAC TCTCTATCAA
L.pubescens                 ------CAAC GGATGGGCAA AAAAACGGGG TCTTCGGTCC TATATTTCAG
L.sativus_KJ                ATTCATTAAG GACGAGACAA ACAAAAAAGT TTTATGTTCC TATGTGTCAG
L.sativus_HM                ATTCATTAAG GACGAGACAA ACAAAAAAGT TTTATGTTCC TATGTGTCAG
L.pseudocicera              ATGCATTAAC GACGAGACAA AGAAAAAAGT TTTGTGTTCT TATGTCACAG
L.chloranthus               ---------- ---------- ---------- ---------- ----------
L.pratensis                 ---------- ---------- ---------- ---------- ----------
L.odoratus_HM               ---------- ---------- ---------- ---------- ----------
L.odoratus_kJ               ---------- ---------- ---------- ---------- ----------
L.hirsutus                  ---------- ---------- ---------- ---------- ----------
L.annuus                    ---------- ---------- ---------- ---------- ----------
L.cirrhosus                 CATAATTCGC TGGGTGACAC GCAAAGAAGT GCTGGATTAT TACGTCGCAC
L.latifolius                CGTAATTCGC TGGGTGACAC GCAAAGAAGT GCTGGATTAT TACGTCTCAC
L.tingitanus                ---------- ---------- ---------- ---------- ----------
Pisum sativum_H             ---TAATATA TAATATATAT AGTAGTCCAA TTTTTGTACG ATGGGACTGT
Pisum sativum_K             ---TAATATA TAATATATAT AGTAGTCCAA TTTTTGTACG ATGGGACTGT
Pisum sativum_HG            ----TATATA TAATATATAT AGTAGTCCAA TTTTTGTACG ATGGGACTGT
Pisum fulvum                ---TAATATA TAATATATAT AGTAGTCCAA TTTTTGTACG ATGGGACTGT
Vavilovia formosa           ---------- ---TATATAT AGTAGTCAGA TTTTTGTCCG ATGGAACTAT
L.clymenum                  ---------A TGGTGATTTT TGCAGTCCTT TCTTTATGGA TTTGTGTTGA
L.ochrus                    TTGAAAAATA TGGTGGGTTT TGCAGTCCTT TCTTTATGGA TTTGTGTTGA
```

```
                             451
Trifolium strictum          AAGACGCATG GCTTGACATT GTACCGGGGT CCCGAAAAAC AATCAATTTC
T. glanduliferum            AAGACGCATG GCTTGACATT GTACCGGGGT CCCGAAAAAC AATCAATTTC
T. boissieri                AAGACGCATG GCTTGACATT GTACCGGGGT CCCGAAAAAC AATCAATTTC
T. aureum                   AAGACGCATG GCTTGACATT GTACCGGGAT CCCGAAAAAC AATCAATTTC
T.meduseum                  AAGACGCATG GCTTGACATT GTACCGGGGT CCAAAAAAAC AATCAATTTC
T.subterraneum              AAGACGCATG GCTTGACATT GTACCGGGGT CCAAAAAAAC AATCAATTTC
T.pratense                  AAGACGCATG GCTTGACATT GTACCGGGGT CCAAAAAAAC AATCAATTTC
T.hybridum                  AAGACGCGTG GCTTGACATT GTACCGGGGT CCAAAAAAAC TATCAATTTC
T.semipilosum               AAGACGCGTG GCTTGACATT GTACCGGGGT CCAAAAAAAC TATCAATTTC
T.occidentale               AAGACGCATG GCTTGACATT GTACCGGGGT CCAAAAAAAC AATCAATTTT
T.repens                    AAGACGCATG GCTTGACATT GTACCGGGGT CCAAAAAAAC AATCAATTTT
T.lupinaster                AAGACGCATG GCTTGACATT GTACCGGGGT CCCAAAAAAC AATCAATTTC
Cicer oxyodon               AAGACGCATG GCTTGACATT GTAACGGGGT CCCGAAAACC AATCAATTTC
C. chorrassanicum           AAGACGCATG GCTTGACATT GTAACGGGGT CCCGAAAACC AATCAATTTC
C. arietinum                AAGACGCATG GCTTGACATT GTAACAGGGT CCCGAAAACC GATCAATTTC
Medicago truncatula_KF      AAGACGCATG GCTTGACATT GTACCGGGGT CCCGAAAACC AATCAATTTC
M.truncatula_AC             AAGACGCATG GCTTGACATT GTACCGGGGT CCCGAAAACC AATCAATTTC
M.sativa_K                  AAGACGCATG GCTTGACATT GTACCGGGGT CCCGAAAACC AATCAATTTC
M.sativa                    AAGACGCATG GCTTGACATT GTACCGGGGT CCCGAAAACC AATCAATTTC
M.papillosa                 AAGACGCATG GCTTGACATT GTACCGGGGT CCCGAAAACC AATCAATTTC
M.hybrida                   AAGACGCATG GCTTGACATT GTACCGGGGT CCCGAAAACC AATCAATTTC
M.falcata                   AAGACGCATG GCTTGACATT GTACCGGGGT CCCGAAAACC AATCAATTTC
M.sativa_KU                 AAGACGCATG GCTTGACATT GTACCGGGGT CCCGAAAACC AATCAATTTC
Melilotus albus             AAGACGCATG GCTTGACATT GTACCGGGGT CCCGAAAACC AATCAATTTC
Hedysarum formosum          AACACACATG GATTTATACT ATACCCGGGG CACGAAAACC AATTAATTTC
H.varium                    AACACACATG GATTTATACT ATACCCGGGG CACGGAAACC AATCAATTTC
H.singarense                AACACAGATG GATTTATACT ATACCCGGGG CACGAAAACC GACCAATTTC
H.minjanense                AACACACATG GATTTACACT ATACCCGGGG CCCGAAAACC AATCAATTTC
Tavrniera glabra            AACACACATG GATTTACACT ATACCCGGGG CCCGAAAACC AATCAATTTC
T.diffusa                   AACACACATG GATTTACACT ATACCCGGGG CCCGAAAACC AATCAATTTC
Greuteria membranacea       AACACACATG GATTTACACT ATACCCGGGG CCCGAAAACC AATCAATTTC
Corethrodendron scoparium   AACACACATG GATTTACACT ATACCCGGGG CCCGAAAACC AATCAATTTC
Eversmannia subspinosa      AACACACATG GATTTACACT ATACCCGGGG CCCGAAAACC AATCAATTTC
Onobrychis bungei           AACACACATG GATTTACACC ATACCCGGGG CCCGAAAACC AATCAATTTC
O.cornuta                   AACACACATG GATTTACACC ATACCCGGGG CCCGAAAACC AATCAATTTC
O.michauxii                 AACACACATG GATTTACACT ATACCCGGGG CCCGAAAACC AATCAATTTC
O.subacaulis                AACACACATG GATTTACATT ATACCCGGGG CCCGAAAACC AATCAATTTC
O.teheranica                AACACACATG GATTTACATT ATACCCGGGG CCCGAAAACC AATCAATTTC
Alhagi maurorum             AACACACATG GATCTACACT ATACCAGGGG CCCGAAAACC AATCAATTTC
Caragana microphylla        AACACACATG GATCTACACT ATACCAGGGG CCCGAAAACC AATCAATTTC
C.korshinski                AACACACATG GATCTACACT ATACCAGGGG CCCGAAAACC AATCAATTTC
C.kozlowii                  AACACACATG GATCTACACT ATACCAGGGG CCCGAAAACC AATCAATTTC
C.rosea                     AACACACATG GATCTACACT ATACCAGGGG CCCGAAAACC AATCAATTTC
Tibetia liangshanensis      AAGACGCATG GCTCTACACT ATACCAGGGG CCCGAAAACC AATCAATTTC
Gueldenstaedtia verna       AAAACCCAGG GCTCTACACT ATACCAGGGG CCCGAAAACC AATCAATTTC
Halimodendron halodendron   AACCTCCCAC CTTTTAAGTT AACCCCGGGG CCCGAAATCT CAA-AACTTC
Smirnowia turkestana        AAGACGCATG GCTCTACACT GTACCAGGGG CCCGAAAACC AATCAATTTC
Eremosparton flaccidum      AAGACGCATG GCTCTACACT GTACCAGGGG CCCGAAAACC AATCAATTTC
Colutea persica             AAGACGCATG GCTCTACACT GTACCAGGGG CCCGAAAACC AATCAATTTT
C. triphylla                AAGACGCATG GCTCTACACT GGACCAGGGG CCCGAAAACC AATTTATTTT
Sphaerophysa salsula        AAGACGCATG GCTCTACACT GTACCAGGGG CCCGAAAACC AATCAATTTC
Podlechiella vogelii        AAGACGCATG GCTCTACACT GTACCAGGGG CCCGAAAACC AATCAATTTC
Carmichaelia australis      AAGACGCATG GCTCTACAGT GTACCAGGGG CCCGAAAACC AATCAATTTC
Sutherlandia frutescens     AAGACGCATG GCTCTACACC GTACCAGGGG CCCGAAAACC AATCAATTTC
Astragalus membranaceous    AAGACGCATG GCTCTACACT GTACCTGGGG CTCGAAAACC AATCAATTTC
A.mongholicus               AAGACGCATG GCTCTACACT GTACCTGGGG CTCGAAAACC AATCAATTTC
A.nakaianus                 AAGACGCATG GCTCTACACT GTACCTGGGG CTCGAAAACC AATCAATTTC
A.macropelmatus             AAGACGCATG GCTCTACACT GTACCAGGGG CCCGAAAACC AATAAATTTA
A.iranicus                  AAGACGCATG GCTCTACACT GTACCTGGGG CCCGAAAACC AATCAATTTC
A.denudatus                 AAGACGCATG GCTCTACACT GTACCTGGGG CCCGAAAACC AATCAATTTC
A.odoratus                  AAGACGCATG GCTCTACACT GTACCTGGGG CCCGAAAACC AATCAATTTC
Oxytropis szovitsii         AAGACGCATG GCTCTACACT GTACCAGGGG CCCGAAAACC AATCAATTTT
O.iranica                   AAGACGCATG GCTCTACACT GTACCAGGGG CCCGAAAACC AATCAATTTT
O.kotschyana                AAGACGCATG GCTCTACACT GTACCAGGGG CCCGAAAACC AATCAATTTT
Erophaca baetica            AAGATGCATG GCTCTACACT GTACCAGGGG CCCGAAAACC TATCAATTTC
Wisteria floribunda         AAGACGGATG GATCGACTTT GTAAAGGGGT GTCGAAAACC AAGCGATTTC
W. sinensis                 AAGACGGATG GATCGACTTT GTAAAGGGGT GTCGAAAACC AAGCGATTTC
Glycyrrhiza lepidota        AAGACGCATG GATCGACATT GTAACGGGGT CTCGAAAACC AAGCGATTTC
G.uralensis                 AAGACGCATG GATCGACATT GTAACGGGGT CTCGAAAACC AAGCGATTTC
G.glabra                    AAGACGCATG GATCGACATT GTAACGGGGT CTCGAAAACC AAGCGATTTC
Meristotropis xathioides    AAGACGCATG GATCGACATT GTAACGGGGT CTCGAAAACC AAGCGATTTC
Robinia pseudoacacia        AAGAAATACG GGTAGATTTT GTAACAGGAT GTCGAAAACC GATTGATTTC
Lotus japonicus             AAGAAGTACG AGTAGATTTT GTAACGGGGT GTCGAAACCC CCGTGATTTC
Vicia alpestris             AAGACCCATC GCTTGACATT GTCCAGGGGT CCCGAAAAAT TATCTATTTC
V.sativa                    AAGACGCATG GCTTGACATT GTACCGGGGT CCCTAAAAAT TATCAATTTC
V.peregrina                 AAGACGCATG GCTTGACATT GTACCGGGGT CCCTAAAAAT TATCAATTTC
V.sepium                    AAGACGCATG GCTTGACATT GTACCGGGGT CCCTAAAAAT TATCAATTTC
V. faba                     AAGACGCATG GCTTGACATT GTACCGGGGT CCCGAAAAAT TATCAATTTA
```

V.canescens	AAGACGCATG	GCTTGACATT	GTACCGGGGT	CCCGAAAAAT	TATCAATTTC
V.monantha	AAGACGCCTG	GCTTGACATT	GTACCGGGGT	CCCGAAAAAT	TATCAATTTA
V.tetrasperma	AAGTAGCATG	GCTTGACATT	GTCCCGGGGT	CCCGAAAAAT	TATCAATTTA
V.narbonensis	AAGACGCATG	GCTTGACATT	GTACCGGGGT	CCCGAAAAAT	TATCAATTTT
Lens culinaris	AAGACGCATG	GCTTGACATT	GTACCGGGGT	CCAAAAAAAT	TATCAATTTC
L.orientalis	AAGACGCATG	GCTTGACATT	GTACCGGGGT	CCAAAAAAAT	TATCAATTTT
V.ervilia	AAGACGCATG	GCTTGACATT	GTACCGGGGT	CCCGAAAAAC	TATCAATTTC
Galega officinalis	AAGACGCATG	GCTTGACATT	GTACCGGGGT	CCCGAAAATC	AATCAATATC
Lathyrus palustris_K	ACGACATCTG	GCTCGATACT	ATACCTGGAT	CCAGAAATGG	TACGATTTTT
L.palustris_H	ACGACATTTG	GGTGGATAGT	ATACCAGGAT	CCAGAAATGG	GACGATTCTT
L.davidii	ACGACATATG	GGTAGATGAT	ATCCCTGGGT	CCAGAAATGG	TACCATTTTT
L.japonicus	AGGACATCTG	GATTGATACG	ATCCCTGGGT	CCAGAAATGG	GACGATTCTT
L.ochroleucus	ACAGCATATG	GGTAGACACT	ATACCTGGGT	CCAGAAATGG	CACGATTCTT
L.littoralis	AGGACATCTG	GTTTGATACT	ATCCCTGGGT	CCAGAAATGG	TACGATTTTG
L.venosus	AAAACGTATG	GATCGATACT	ATACCTGGAT	CGAGAAATGC	TACGATTCTT
L.graminifolius	AGGACATCTG	GATCGATATT	ATCCCCGGAT	CCAGAAATGG	TACGATTCTT
L.pubescens	AAGATATATG	GATTGACACG	ATACCTGGGT	CTCGAAATGG	TAACATTCTT
L.sativus_KJ	AAACTACTTG	GATGGACACT	ATCCCGGGTT	TCCAACACGT	TTTTTTTCGA
L.sativus_HM	AAACTACTTG	GATGGACACT	ATCCCGGGTT	TCCAACACGT	TTTTTTTCGA
L.pseudocicera	AAAATACTTG	GATGGACACT	ATCCCGGGTT	TCCAACATGT	GACTTTTAGA
L.chloranthus	----------	----------	----------	----------	----------
L.pratensis	----------	----------	----------	----------	----------
L.odoratus_HM	----------	----------	----------	----------	----------
L.odoratus_kJ	----------	----------	----------	----------	----------
L.hirsutus	----------	----------	----------	----------	----------
L.annuus	----------	----------	----------	----------	----------
L.cirrhosus	AAGACATTTG	GATTGACACT	ATCCCGGGTT	TCCGAAATGT	TTGGTTTCTC
L.latifolius	AAGACATTTG	GATTGACACT	ATCCCGGGTT	TCCGAAATGT	TTGGTTTCTC
L.tingitanus	----------	----------	----------	----------	----------
Pisum sativum_H	AGAGGGTATT	ATAGTAACTG	TTTTATCGCT	TGTTTTTGCT	AGAAAATTAA
Pisum sativum_K	AGAGGGTATT	ATAGTAACTG	TTTTATCGCT	TGTTTTTGCT	AGAAAATTAA
Pisum sativum_HG	AGAGGGTATT	ATAGTAACTG	TTTTATCGCT	TGTTTTTGCT	AGAAAATTAA
Pisum fulvum	AGAGGGTATT	ATAGTAACTG	TTTTATCGCT	TGTTTTTGCT	AGAAAATTAA
Vavilovia formosa	AACGTGTATT	ATAGTCACTG	GTTTATGGCT	CGTTTTTCCT	ACAAAATTAA
L.clymenum	AAAAGTCATT	C--GTCGCTT	GTCAAAAAAC	CGAGGTTTTC	GTTCATATCT
L.ochrus	AAAAGTCATT	C--GTCGCTT	GTCAAAAAAC	CGAGGTTTTC	GTTCATATCT

```
                             501
Trifolium strictum          TTCTGGGCTT CTTTCACTCT TTTAGGTTCA TTAGGGGTGT TATATATTTC
T. glanduliferum            TTCTGGGCTT CTTTCACTCT TTTAGGTTCA TTAGGGGTGT TATATATTTC
T. boissieri                TTCTGGGCTT CTTTCACTCT TTTAGGTTCA TTAGGGGTGT TATATATTTC
T. aureum                   TTCTGGGCTT CTTTCACTCT TTTAGGTTCA TTAGGGGTGT TATATATTTC
T.meduseum                  TTCTGGGCTT CTTTCACTCT TTTAGGTTCA TTAGGGGTGT TATATATTTC
T.subterraneum              TTCTGGGCTT CTTTCACTCT TTTAGGTTCA TTAGGGGTGT TATATATTTC
T.pratense                  TTCTGGGCTT CTTTCACTCT TTTAGGTTCA TTAGGGGTGT TATATATTTC
T.hybridum                  TTCTGGGCTT CTTTCACTCT TTTAGGTTCA TTAGGGGTCT TATATATTTC
T.semipilosum               TTCTGGGCTT CTTTCACTCT TTTAGGTTCA TTAGGGGTCT TATATATTTC
T.occidentale               TTCTGGGCTT CTTTCACTCT TTTAGGTTCA TTAGGGGTGT TATATATTTC
T.repens                    TTCTGGGCTT CTTTCACTCT TTTAGGTTCA TTAGGGGTGT TATATATTTC
T.lupinaster                TTCTGGGCTT CTTTCACTCT TTTAGGTTCA TTAGGGGTGT TATATATTTC
Cicer oxyodon               TTCTGGGCTT CTTTCACTCT TTTAGGTTCA TTAGGAGTGT TATATATTTC
C. chorrassanicum           TTCTGGGCTT CTTTCACTCT TTTAGGTTCA TTAGGGGTGT TATATATTTC
C. arietinum                TTCTGGGCTT CTTTCACTCT TTTAGGTTCA TTAGGAGTGT TATATATTTC
Medicago truncatula_KF      TTCTGGGCTT CTTTCACTCT TTTAGGTTCA TTAGGGGTGT TATATCTTTC
M.truncatula_AC             TTCTGGGCTT CTTTCACTCT TTTAGGTTCA TTAGGGGTGT TATATCTTTC
M.sativa_K                  TTCTGGGCTT CTTTCACTCT TTTAGGTTCA TTAGGGGTGT TATATATTTC
M.sativa                    TTCTGGGCTT CTTTCACTCT TTTAGGTTCA TTAGGGGTGT TATATATTTC
M.papillosa                 TTCTGGGCTT CTTTCACTCT TTTAGGTTCA TTAGGGGTGT TATATATTTC
M.hybrida                   TTCTGGGCTT CTTTCACTCT TTTAGGTTCA TTAGGGGTGT TATATATTTC
M.falcata                   TTCTGGGCTT CTTTCACTCT TTTAGGTTCA TTAGGGGTGT TATATATTTC
M.sativa_KU                 TTCTGGGCTT CTTTCACTCT TTTAGGTTCA TTAGGGGTGT TATATATTTC
Melilotus albus             TTCTGGGCTT CTTTCACTCT TTTAGGTTCA TTAGGGGTAT TATATATTTC
Hedysarum formosum          TTCTGGGCTT CTTTCACTCT TTTAGGTTCA TTAGGGGTTT TATTTATTTC
H.varium                    TTCTGGGCTT CTTTCACTCT TTTAGGTTCA TTAGGGGTTT TATTTATTTC
H.singarense                TTCTGGGCTT CTTTCACTCT TTTAGGTTCA TTAGGGGTTT TATTTATTTC
H.minjanense                TTCTGGGCTT CTTTCACTCT TTTAGGTTCA TTAGGGGTTT TATTTATTTC
Tavrniera glabra            TTCTGGGCTT CTTTCACTCT TTTAGGTTCA TTAGGGGTTT TATTTATTTC
T.diffusa                   TTCTGGGCTT CTTTCACTCT TTTCGGTTCA TTAGGGGTTT TATTTATTTC
Greuteria membranacea       TTCTGGGCTT CTTTCACTCT TTTAGGTTCA TTAGGGGTTT TATTTATTTC
Corethrodendron scoparium   TTCTGGGCTT CTTTCACTCT TTTAGGTTCA TTAGGGGTTT TATTTATTTC
Eversmannia subspinosa      TTCTGGGCTT CTTTCACTCT TTTAGGTTCA TTAGGGGTTT TATTTATTTC
Onobrychis bungei           TTCTGGGCTT CTTTCACTCT TTTAGGTTCA TTAGGGGTTT ATTTTATTTC
O.cornuta                   TTCTGGGCTT CTTTCACTCT TTTAGGTTCA TTAGGGGTTT TATTTATTTC
O.michauxii                 TTCTGGGCTT CTTTCACTCT TTTAGGTTCA TTAGGGGTTT TATTTATTTC
O.subacaulis                TTCTGGGCTT CTTTCACTCT TTTAGGTTCA TTAGGGGTTT TATTTATTTC
O.teheranica                TTCTGGGCTT CTTTCACTCT TTTAGGTTCA TTAGGGGTTT TATTTATTTC
Alhagi maurorum             TTCTGGGCTT CTTTCACTCT TTTAGGTTCA TTAGGGGTGT TATTTATTTC
Caragana microphylla        TTCTGGGCTT CTTTCACTCT TTTAGGTTCA TTAGGGGTGT TATTTATTTC
C.korshinski                TTCTGGGCTT CTTTCACTCT TTTAGGTTCA TTAGGGGTGT TATTTATTTC
C.kozlowii                  TTCTGGGCTT CTTTCACTCT TTTAGGTTCA TTAGGGGTGT TATTTATTTC
C.rosea                     TTCTGGGCTT CTTTCACTCT TTTAGGTTCA TTAGGGGTTT TATTTATTTC
Tibetia liangshanensis      TTCTGGGCTT CTTTCACTCT TTTAGGTTCA TTAGGGGTGT TATTTATTGC
Gueldenstaedtia verna       TTCTGGGCTT CTTTCACTCT TTTAGGTTCA TTAGGGGTGT TATTTATTGC
Halimodendron halodendron   TTCTAGGCCT CTTCCGTTTT ATT-GTTACG GAGGGGGGGG CAATTGTTA-
Smirnowia turkestana        TTCTGGGCTT CTTTCACTCT TTTAGGTTCG TTAGGGGTTT TAGTTATTTC
Eremosparton flaccidum      TTCTGGGCTT CTTTCACTCT TTTAGGTTCG TTAGGGGTTT TAGTTATTTC
Colutea persica             TTCTGGGCTT CTTTCACTCT TTTAGGTTCA TTAGGGGTTT TAGTTATTTC
C. triphylla                TTCTGGGCTT CTTTCACTCT TTTAGGTTCA TTAGGGGTTT TAGTTATCTC
Sphaerophysa salsula        TTCTGGGCTT CTTTCACTCT TTTAGGTTCA TTAGGGGTTT TAGTTATTTC
Podlechiella vogelii        TTCTGGGCTT CTTTCACTCT TTTAGGTTCA TTAGGGGTTT TAGTTATTTC
Carmichaelia australis      TTCTGGGCTT CTTTCACTCT TTTAGGTTCA TTAGGGGTTT TAGTTATTTC
Sutherlandia frutescens     TTCTGGGCTT CTTTCACTCT TTTAGGTTCA TTAGGAGTTT TAGTTATTTC
Astragalus membranaceous    TTCTGGGCTT CTTTCACTCT TTTAGGTTCA TTAGGGGTTT TAGTTATTTC
A.mongholicus               TTCTGGGCTT CTTTCACTCT TTTAGGTTCA TTAGGGGTTT TAGTTATTTC
A.nakaianus                 TTCTGGGCTT CTTTCACTCT TTTAGGTTCA TTAGGGGTTT TAGTTATTTC
A.macropelmatus             TTCTGGGCTT CTTTCACCCT TTTAGGTTCA TTAGGGGTTT TAGTTATTTC
A.iranicus                  TTTTGGGCTT CTTTCACTCT TTTAGGTTCA TTAGGGGTTT TAGTTATTTC
A.denudatus                 TTCTGGGCTT CTTTCACTCT TTTAGGTTCT TTAGGGGTTT TAGTAATTTC
A.odoratus                  TTCTGGGCTT CTTTCACTCT TTTAGGTTCA TTAGGGGTTT TAGTTATTTC
Oxytropis szovitsii         TTCTGGGCTT CTTTCACTCT TTTAGGTTCA TTAGGGGTTT TAGTTGTTTC
O.iranica                   TTCTGGGCTT CTTTCACTCT TTTAGGTTCA TTAGGGGTTT TAGTTGTTTC
O.kotschyana                TTCTGGGCTT CTTTCACTCT TTTAGGTTCA TTAGGGGTTT TAGTTATTTC
Erophaca baetica            TTCTGGGCTT CTTTCACCCT TTTAGGTTCA TTAGGGGTTT TATTTATTTC
Wisteria floribunda         TTCTGGGCTT CTTTCATTCT TTTAGGTTCA TTAGGGGTGT TATTGATTTC
W. sinensis                 TTCTGGGCTT CTTTCATTCT TTTAGGTTCA TTAGGGGTGT TATTGATTTC
Glycyrrhiza lepidota        TTCTGGGCTT CTTTCATTCT TTTAGGTTCA TTAGGGGTTT TATTGATTTC
G.uralensis                 TTCTGGGCTT CTTTCATTCT TTTAGGTTCA TTAGGGGTTT TATTGATTTC
G.glabra                    TTCTGGGCTT CTTTCATTCT TTTAGGTTCA TTAGGGGTTT TATTGATTTC
Meristotropis xathioides    TTCTGGGCTT CTTTCATTCT TTTAGGTTCA TTAGGGGTTT TATTGATTTC
Robinia pseudoacacia        TTCTCGGCCT CTATCAGTCT TTTTGGTTCA TTAGGGGGTT TATTGGTTGC
Lotus japonicus             TTCTGGGCAT TTATCACTCT TTTAGGTTCA GTAGGGTTTT TATTGGTTGC
Vicia alpestris             TTCTGGGCTT CTTTCACTCT TTTAGGTTCC TTAGGGGGTG TATATTTTTC
V.sativa                    TTCTGGGCTT CTTTCACTCT TTTAGGTTCA TTAGGGGTTT TATATATTTC
V.peregrina                 TTCTGGGCTT CTTTCACTCT TTTAGGTTCA TTAGGGGTTT TATATATTTC
V.sepium                    TTCTGGGCTT CTTTCACTCT TTTAGGTTCA TTAGGGGTTT TATATATTTC
V. faba                     TTCTGGGCGT CTTTCACTCT TTTAGGTTCA TTAGGAGTTT TATATATTTC
```

```
V.canescens               TTCTGGGCTT CTTTCACTCT TTTAGGTTCA TTGGGGGTTT TATCTATTTC
V.monantha                TTCTGGGCTT CTTTCACTCT TTTAGGTTCA TTAGGGGTTT TATATATTTC
V.tetrasperma             CTCTGGGTCT CTTTCACTCT TTTAGGTTCA TTAGGGGTTT TATATACTTC
V.narbonensis             TTCTGGGCTT CTTTCACTCT TTTAGGTTCA TTAGGGGTTT TATATATTTC
Lens culinaris            TTCTGGGCTT CTTTCACTCT TTTAGGGTCA TTAGGGGTTT TATATATTTC
L.orientalis              TTTTGGGCTT CTTTCACTCT TTTAGGGTCA TTAGGGGTTT TATATATTTC
V.ervilia                 TTCTGGGCTT CTTTCACTCT TTTAGGTTCA TTAGGGGTTT TATATATTTC
Galega officinalis        TTCTGGGCTT CTTTCATTCT TTTAGGTTCA TTAGGGGTAT TATATATTTC
Lathyrus palustris_K      TTGTTTTCGT ACTTTAATTT CTCTCAAACA ATCGAAGGAG TATGCCGTTT
L.palustris_H             TTATTTTCGT ACTTTAATTT CTATCAAACA ATCGAAGGAG TATGTCGTTT
L.davidii                 TTATTTTCGT ACTTTAATTT CCTTCAAACG ATAGAAGGAA TATGCCGTTT
L.japonicus               TTATTTTCTT ACTTTAATTT CTATCAAACA ATTGAAGGAA TATGCCGTTT
L.ochroleucus             TTGTTTTCGT ACTTACATTT CTATCAAACA ATCGAAGGAA TATCCCGTTT
L.littoralis              TTATTTTCTT ACTTTAATTT CTATCAAACA ATTGAAGGAA TATGCCGTTT
L.venosus                 TTATTTTCTT ACTTCAATTT CTCTCAAACA ATCGAAGGAG TATCCCGTTT
L.graminifolius           TTATTTTCTT ATTTAAATTT CTCGCTAACA CTAGAGGGAG TATCCCGTTT
L.pubescens               TTATTTGCCG TTTTCGGTTT CTATAAGTTC ATCGAGTTAG GATACCAGGG
L.sativus_KJ              ATTTTAAGTC ATGTCAGTTT TTTCGTGGGA TTCTTTTCTC TCTATCGTTT
L.sativus_HM              ATTTTAAGTC ATGTCAGTTT TTTCGTGGGA TTCTTTTCTC TCTATCGTTT
L.pseudocicera            ATCTTGAGTC ATGTGAGTTT TTTCATGGGA TTCTTTGCTC TCTATCGTTT
L.chloranthus             ---------- ---------- ---------- ---------- ----------
L.pratensis               ---------- ---------- ---------- ---------- ----------
L.odoratus_HM             ---------- ---------- ---------- ---------- ----------
L.odoratus_kJ             ---------- ---------- ---------- ---------- ----------
L.hirsutus                ---------- ---------- ---------- ---------- ----------
L.annuus                  ---------- ---------- ---------- ---------- ----------
L.cirrhosus               GTGTGTTCTT ATCTCAATTT TTATTTCGGG TTAGATTCTC TATCTCGTTT
L.latifolius              TTGTGTTCTT ATCTCAATTT TTCTTTAGGG TTCGATTCTC TATATCGTTT
L.tingitanus              ---------- ---------- ---------- ---------- ----------
Pisum sativum_H           TTTGTTGGGC AGAAGGTCTT CCTGCATATC GTTTAGAAGA CTCATGGATT
Pisum sativum_K           TTTGTTGGGC AGAAGGTCTT CCTGCATATC GTTTAGAAGA CTCATGGATT
Pisum sativum_HG          TTTGTTAGGC AGAAGGTCTT CCTGCATATC GTTTAGAAGA CTCATGGATT
Pisum fulvum              TTTGTTGGGC AGAAGGTCTT CCTGCATATC GTTTAGAAGA CTCATGGATT
Vavilovia formosa         TT-------- ---------- ---------- ---------- ----------
L.clymenum                TTCAGAAAAC GTATGGATTG ACAGTATACC GGGGTCCCGA AACAGTACCA
L.ochrus                  TTCAGAAAAC GCATGGATTG ACAGAATACC GGGGTCCCGA AACAGTACCA
```

	551				
Trifolium strictum	CGTTTCCAGT	TATTATGGTA	GGCATTTTTT	CTCTTTCATT	TCGTCCGAAT
T. glanduliferum	CGTTTCCAGT	TATTATGGTA	GGCATTTTTT	CTCTTTCATT	TCATCCGA--
T. boissieri	CGTTTCCAGT	TATTATGGTA	GGCATTTTTT	CTCTTTCATT	TCGTCCGAAT
T. aureum	CGTTTCCAGT	TATTATGGTA	GGCATTTTTT	CTCTTTCATT	TCGTCCGATT
T.meduseum	CGTTTCCAGT	TATTATGGTA	GGCATTTTTT	CTCTTTCATT	TCGTCCGAAT
T.subterraneum	CGTTTCCAGT	TATTATGGTA	GGCATTTTTT	CTCTTTCATT	TCGTCCGAAT
T.pratense	CGTTTCCAGT	TATTATGGTA	GGCATTTTTT	CTCTTTCATT	TCGTCCGAAT
T.hybridum	CGTTTCCAGT	TACTACGGTA	GGCATTTTTT	CTCTTTCATT	TCGTCCGAAT
T.semipilosum	CGTTTCCAGT	TACTATGGTA	GGCATTTTTT	CTCTTTCATT	TCGTCCGAAT
T.occidentale	CGTTTCCAGT	TACTATGGTA	GGCATTTTTT	CTCTTTCATT	TCGTCCGAAT
T.repens	CGTTTCCAGT	TACTATGGTA	GGCATTTTTT	CTCTTTCATT	TCGTCCGAAT
T.lupinaster	CGTTTCCAGT	TATTATGGTA	GGCATTTTTT	CTCTTTCATT	TCGTCCGAAT
Cicer oxyodon	AGTTTCTAGT	TATTATGGTA	GGCATTTTTT	CTCTTTCATT	TCGTCCGAAT
C. chorrassanicum	AGTTTCTAGT	TATTATGGTA	GGCATTTTTT	CTCTTTCATT	TCGTCCGAAT
C. arietinum	AGTTTCTAGT	TATTATGGTA	GGCAGTTTTT	CTCTTTCATT	TCGTCCGAAT
Medicago truncatula_KF	AGTTTCCAGT	TATTATGGTA	GACATTTTTT	CTCTTTGATT	TCATCTGAAT
M.truncatula_AC	AGTTTCCAGT	TATTATGGTA	GACATTTTTT	CTCTTTGATT	TCATCTGAAT
M.sativa_K	AGTTTCCAGT	TATTATGGTA	GACATTTTTT	CTCTTTGATT	TCATCTGAAT
M.sativa	AGTTTCCAGT	TATTATGGTA	GACATTTTTT	CTCTTTGATT	TCATCTGAAT
M.papillosa	AGTTTCCAGT	TATTATGGTA	GACATTTTTT	CTCTTTGATT	TCATCTGAAT
M.hybrida	AGTTTCCAGT	TATTATGGTA	GACATTTTTT	CTCTTTGATT	TCATCTGAAT
M.falcata	AGTTTCCAGT	TATTATGGTA	GACATTTTTT	CTCTTTGATT	TCATCTGAAT
M.sativa_KU	AGTTTCCAGT	TATTATGGTA	GACATTTTTT	CTCTTTGATT	TCATCTGAAT
Melilotus albus	CGTTTCCAGT	TATTATGGTA	GACATTTTTT	CTCTTTCATT	TCATCCGAAT
Hedysarum formosum	AGCTTCCAGT	TATTATGGTA	GTAATTTTTT	C------AAT	TCGTCCGAAT
H.varium	AGCTTCCAGT	TATTATGGTA	GTAATTTTTT	C------AAT	TCGTCCGAAT
H.singarense	AGCTACCAGT	TATTATGGTA	GTAATTTTTT	C------AAT	CCGTCCGAAT
H.minjanense	AGCTTCCAGT	TATTATGGTA	GTCATTTTTT	C------AAT	TCGTCCGAAT
Tavrniera glabra	AGCTTCCAGT	TATTATGGTA	GTAATTTTTT	C------AAT	TCGTCCGAAT
T.diffusa	AGCTTCCAGT	TATTATGGTA	GTAATTTTTT	C------AAT	TCGTCCGAAT
Greuteria membranacea	AGCTTCTAGT	TATTATGGTA	GTAATTTTTT	C------AAT	TCGTCCGAAT
Corethrodendron scoparium	AGCTTCTAGT	TATTATGGTA	GTAATTTTTT	C------AAT	TCGTCCGAAT
Eversmannia subspinosa	AGCTTCTAGT	TATTATGGTA	ATAATTTTTT	C------AAT	TCGTCCGAAT
Onobrychis bungei	AGCTTCGAGT	TATTATGGTA	GTAATTTTTT	C------AAT	TCGTCTGAAT
O.cornuta	AGCTTCGAGT	TATTATGGTA	GTAATTTTTT	C------AAT	TCGTCCGAAT
O.michauxii	AGCTTCCAGT	TATTATGGTA	GTAATTTTTT	C------AAT	TCGTCTGAAT
O.subacaulis	AGCTTCCAGT	TATTATGGTA	GTAATTTTTT	C------AAT	TCGTCCGAAT
O.teheranica	AGCTTCCAGT	TATTATGGAA	GTAATTTTTT	C------AAT	TCGTCCGAAT
Alhagi maurorum	AGCTTCTAGT	TATTATGGTA	GCAATTTTTT	C------AAT	TCGTCCGAAT
Caragana microphylla	AGCTTCCAGT	TTTTATGGTA	GGAATTTTTT	CTCTTTCAAT	TCGTCCGAAT
C.korshinski	AGCTTCCAGT	TTTTATGGTA	GGAATTTTTT	CTCTTTCAAT	TCGTCCGAAT
C.kozlowii	AGCTTCCAGT	TTTTATGGTA	GGAATTTTTT	CTCTTTCAAT	TCGGACGAAT
C.rosea	AGCTTCCAGT	TTTTATGGTA	GGAATTTTTT	CTCTTTCAAT	TCGTCCGAAT
Tibetia liangshanensis	AGCTTCCAGT	TATTTTGGTA	GGAATTTTTT	CTCTTTTAAT	TCGTCCGACT
Gueldenstaedtia verna	AGCTTCCAGT	TATTTTGGTA	GGAATTTTTT	CTCTTTGAAT	TCGTCCGACT
Halimodendron halodendron	GGGTCCCAGT	CTTTATGGAA	GAAATATTTA	T--CTTCTAT	TGTTTGGAAT
Smirnowia turkestana	AGCTTCCAGT	TATTATGGTC	GGAATTTTTT	CTCTTTCAAT	TCGGACGAAT
Eremosparton flaccidum	AGCTTCCAGT	TATTATGGTC	GGAATTTTTT	CTCTTTCAAT	TCGGACGAAT
Colutea persica	AGCTTCCAGT	TATTATGGTC	GGAATTTTTT	CTCTTTCAAT	TCGGACGAAT
C. triphylla	AGCTTCCAGT	TATTATGGTC	GGAATTTTTT	CTCTTTCAAT	TCGGACGAAT
Sphaerophysa salsula	AGCTTCCAGT	TATTATGGTC	GGAATTTTTT	CTCTTTCAAT	TCGGACGAAT
Podlechiella vogelii	AGCTTCCAGT	TATTATGGTC	GGAATTTTTT	ATCTTTCAAT	TCAGACGAAT
Carmichaelia australis	CGCTTCCAGT	TATTTTGGTC	GGAATTTTTT	CTCTTTAAAT	TCGGACGAAT
Sutherlandia frutescens	AGCTTCCAGT	TATTATGGTC	GGAATTTTTT	CTCTTTCAAT	TCGGGCGAAT
Astragalus membranaceous	AGCTTCCAGT	TATTATGGTC	GGAATTTTTT	CTCTTTCAAT	TCGTCCGAAT
A.mongholicus	AGCTTCCAGT	TATTATGGTC	GGAATTTTTT	CTCTTTCAAT	TCGTCCGAAT
A.nakaianus	AGCTTCCAGT	TATTATGGTC	GGAATTTTTT	CTCTTTCAAT	TCGTCCGAAT
A.macropelmatus	AGCTTCCAGT	TATTATGGTC	GGAATTTTTT	CTCTTTCAAT	TCGTCCGAAT
A.iranicus	AGCTTCCAGT	TATTATGGTC	GGAATTTTTT	CTCTTTCAAT	TCGTCCGAAT
A.denudatus	AGCTTCCAGT	TATTATGGTC	GGAATTTTTT	CTCTTTCAAT	TCGTCCGAAT
A.odoratus	AGCTTCCAGT	TATTATGGTC	GGAATTTTTT	CTCTTTCAAT	TCGTCCGAAT
Oxytropis szovitsii	AGCTTCCAGT	TATTATGGTC	GGAATTTTTT	CTCTTTCAAT	TCGTCCGAAT
O.iranica	AGCTTCCAGT	TATTATGGTC	GGAATTTTTT	CTCTTTCAAT	TCGTCCGAAT
O.kotschyana	AGCTTCCAGT	TATTATGGTC	GGAATTTTTT	CTCTTTCAAT	TCGTCCGAAT
Erophaca baetica	AGCTTCGAGT	TATTATGGTT	GGAATTTTTT	CTCTTTCAAT	TCGTCCGAAT
Wisteria floribunda	AGCTTCCAGT	TTTTATGGTA	GGCATTTTTT	ATCTTTCATT	TCGTCTGAGT
W. sinensis	AGCTTCCAGT	TTTTATGGTA	GGCATTTTTT	ATCTTTCATT	TCGTCTGAGT
Glycyrrhiza lepidota	AGCTTCCAGT	TATTATGGCA	GGAATTTGTT	ATCTTTCATT	TCGTCGGAGT
G.uralensis	AGCTTCCAGT	TATTATGGCA	GGAATTTGTT	ATCTTTCATT	TCGTCGGAGT
G.glabra	AGCTTCCAGT	TATTATGGCA	GGAATTTGTT	ATCTTTCATT	TCGTCGGAGT
Meristotropis xathioides	AGCTTCCAGT	TATTATGGCA	GGAATTTGTT	ATCTTTCATT	TCGTCGGAGT
Robinia pseudoacacia	AGCTTCCAGT	TATGATGGAA	TGAATTACTT	ATATTTCATT	TCGTATGAGT
Lotus japonicus	AGCTTCCAGT	TATCTTCATA	AGAATTTTTT	ATCTTTCATT	TCGTCTGAGT
Vicia alpestris	TGTTTCCCGT	TATTATCGGG	GGGCTTTTTC	CCTCTTCATT	TCGTCGGAAT
V.sativa	AGTTTCCAGT	TATTATGGGG	GGGATTTTTT	CTCTTTCATT	TCGTCCGAAT
V.peregrina	AGTTTCCAGT	TATTATGGGG	GGGATTTTTT	TTCTTTCATT	TCGTCCGAAT
V.sepium	AGTTTCCAGT	TATTATGGGG	GGGATTTTTT	CTCTTTCATT	TCGTCCGAAT
V. faba	AGTTTCCAGT	TATTATGGGA	GGGATTTTTT	CTCTTTCATT	TCGTCCGAAT

```
V.canescens               AGTTTCCAGT TATTATGGGA GGGATTTTTT CCCTTTCATT TCGTCCGAAT
V.monantha                AGTTTCCAGT TATTATGGGA GGTATTTTTT CCCTTTCATT TCGTCCGAAT
V.tetrasperma             AGTTTCCAGA TATTATGGGA GGGATTTATT CTCTTTGATT TCGTCCGAAT
V.narbonensis             AGTTTCCAGT TATTATGGGA GGGATTTTTT CTCTTTCATT TCGTCCGAAT
Lens culinaris            AGTTTCCAGT TATTATGGGA GGGATTTTTT CTCTTTCATT TCGTCCGAAT
L.orientalis              AGTTTCCAGT TATTATGGGA GGGATTTTTT CTCTTTCCAT TCGTCCGAAT
V.ervilia                 TGTTTCCAGT TATTATGGGA GGGATTTTTT TTCTTTCATT TCGTCCGAAT
Galega officinalis        AGTTTCCAGT TATTATGGTA GGTCTTTTTT CTCTTTCATT TCGTCCGAAG
Lathyrus palustris_K      GTTTGGCGGG TTGTACGACG ACAGAATGTT TTTCGATTTG AGGGTCTCGG
L.palustris_H             GTTTGACGGG TTGTACGACG ACAGAATGTT TTTCGATTTG AGGGTCTCGG
L.davidii                 GTTTGGCGCA CTCTACGACG ACAGAATGTT TCTGGATTTG A------CGG
L.japonicus               GTTTGGCGCG TTATACGAGG ACAGACTGTT TCTCGATTTG AGGGTCTCGG
L.ochroleucus             GTTTAGCGAA ATATATTATG ACAGGCTGTT TCTCGATTTG AGGGTCGCGG
L.littoralis              GTTTGGCGCG CTGTACGAGG ACAGAATGTT TCTCGATTTG AGGGTCTCGG
L.venosus                 GTTTGGCGAA TTATATTATG ACAGGCTGTT TCTCGATTTG AAGGTCGCGG
L.graminifolius           GTGTCACGAG TTGTTTTACG ATTCCCTCTA TCTCGATTTG AGGGTCTCGG
L.pubescens               GTCTAAAAGA ATCTCTGGAT ATTTCTTGAT CGCGTTCCCT ATTACCCCTA
L.sativus_KJ              CTATAG---C AATCTTACTC GGATGAACAT TTTCGATGCA CCGATT-AAA
L.sativus_HM              CTATAG---C AATCTTACTC GGATGAACAT TTTCGATGCA CCGATT-AAA
L.pseudocicera            CTATAG---C AATCTTACTC GGATGAACAT TTTTGATGCA CCGATT-ACA
L.chloranthus             ---------- ---------- ---------- ---------- ----------
L.pratensis               ---------- ---------- ---------- ---------- ----------
L.odoratus_HM             ---------- ---------- ---------- ---------- ----------
L.odoratus_kJ             ---------- ---------- ---------- ---------- ----------
L.hirsutus                ---------- ---------- ---------- ---------- ----------
L.annuus                  ---------- ---------- ---------- ---------- ----------
L.cirrhosus               CTATATTTTC AATAAGGTAA GCACTGAATT TTTCGAGTCC CCGATT-GAC
L.latifolius              CTATATTTTC AATAAGATAA GCACTGAATT TTTCGAGTCC CCGATT-GAC
L.tingitanus              ---------- ---------- ---------- -------ATG CCGACC-CGC
Pisum sativum_H           GCCTTTGTAG CAGGGTCCCA AAACAGTTTC ACTATTTTGT GTGGTTCTTT
Pisum sativum_K           GCCTTTGTAG CAGGGTCCCA AAACAGTTTC ACTATTTTGT GTGGTTCTTT
Pisum sativum_HG          GCCTTTGTAG CAGGGTCCCA AAACAGTTTC ACTATTTTGT GTGGTTCTTT
Pisum fulvum              GCCTTTGTAG CAGGGTCCCA AAACAGTTTC ACTATTTTGT GTGGTTCTTT
Vavilovia formosa         ---------- ---------- ---------- ---------- --GGTTTTTT
L.clymenum                ATATCTTCTT TGGTTATTTC CATCTTTTAC AGTCATTATG GCGCGTATAT
L.ochrus                  ATATCTTCTT TGGTTATTTC CATCTTTTAC AGTCATTATG GCGCGTATAT
```

```
                             601
Trifolium strictum          TTG------- --TAGTTCCT TTTTTGCCAC AAGGGGTCAC GCTTACTTTC
T. glanduliferum            -TG------- --TAATTCCT TTTTTGCCAC AAGGGGTCAC GCTTACTTTC
T. boissieri                TTG------- --TAGTTCCT TTTTTGCCAC AAGGGGTCAC GCTTACTTTC
T. aureum                   TTG------- --TAGTTCCG TTTTTGCCAC AAGGGGTCAC GCTTACTTTC
T.meduseum                  TTG------- --TAGTTCCT TTTTTGCCAC AAGGGGTCAC GCTTACTTTC
T.subterraneum              TTG------- --TAGTTCCT TTTTTACCAC AAGGGGTCAC GCTTACTTTC
T.pratense                  TTG------- --TAGTTCCT TTTTTGCCAC AAGGGGTCAC GCTTACTTTC
T.hybridum                  TTG------- --TAGTTCCT TTTTTGCCAC AAGGGGTCAC GCTTACTTTC
T.semipilosum               TTG------- --TAGTTCCT TTTTTGCCAC AAGGGGTCAC GCTTACTTTC
T.occidentale               TTG------- --TAGTTCCT TTTTTGCCAC AAGGGGTCAC GCTTACTTTC
T.repens                    TTG------- --TAGTTCCT TTTTTGCCAC AAGGGGTCAC GCTTACTTTC
T.lupinaster                TTG------- --TAATTCCT TTTTTGCCAC AAGGGGTCAC GCTTACTTTC
Cicer oxyodon               TTG------- --TAGTTCCT TTTTTGCCAC AAGGGGTCAC ACTTACTTTC
C. chorrassanicum           TTG------- --TAGTTCCT TTTTTGCCAC AAGGGGTCAC ACTGACTTTC
C. arietinum                TTG------- --TAGTTCCT TTTTTGCCAC AAGGGGTCAC ACTGACTTTC
Medicago truncatula_KF      TTG------- --TAGTTCCT TTTTTGCCAC AAGGGGTCAC CCTGACTTTC
M.truncatula_AC             TTG------- --TAGTTCCT TTTTTGCCAC AAGGGGTCAC CCTGACTTTC
M.sativa_K                  TTG------- --TAGTTCCT TTTTTGCCAC AAGGGGTCAC CCTGACTTTC
M.sativa                    TTG------- --TAGTTCCT TTTTTGCCAC AAGGGGTCAC CCTGACTTTC
M.papillosa                 TTG------- --TAGTTCCT TTTTTGCCAC AAGGGGTCAC CCTGACTTTC
M.hybrida                   TTG------- --TAGTTCCT TTTTTGCCAC AAGGGGTCAC CCTGACTTTC
M.falcata                   TTG------- --TAGTTCCT TTTTTGCCAC AAGGGGTCAC CCTGACTTTC
M.sativa_KU                 TTG------- --TAGTTCCT TTTTTGCCAC AAGGGGTCAC CCTGACTTTC
Melilotus albus             TTG------- --TAGTTCCC TTTTTGCCAC AAGGGGTCAC CCTGACTTTC
Hedysarum formosum          CTG------- --TAGTTCCT TTTTTGCCAC AAGGGGCCAC GCTGACTTTC
H.varium                    CTG------- --TAGTTCCT TTTTTGCCAC AAGGGGCCAC GCTGACTTTC
H.singarense                CTG------- --TAGTTCCT TTTTTGCCAC AAGGGGCCAC GCTTACTTTC
H.minjanense                CTG------- --TAGTTCCT TTTTTGCCAC AAGGGGCCAC ACTGACTTTC
Tavrniera glabra            CTG------- --TAGTTCCT TTTTTGCCAC AAGGGGCCAC GCTAACTTTC
T.diffusa                   CTG------- --TAGTTCCT TTTTTGCCAC AAGGGGCCAC GCTAACTTTC
Greuteria membranacea       CTG------- --TAGTTCCT TTTTTGCCAC AGGGGGCTAC GCTGACTTTC
Corethrodendron scoparium   CTG------- --TAGTTCCT TTTTTGCCAC AGGGGGCCAC GCTGACTTTC
Eversmannia subspinosa      TTG------- --TAGTTCCC TTTTTGCCAC AGGGGGCCAC GCTGACTTTC
Onobrychis bungei           CTG------- --TAGTTCCT TTTTTGCCAC AGGGGGCCAC ACTCACTTTC
O.cornuta                   CTG------- --TAGTTCCT TTTTTGCCAC AGGGGGCCAC GCTCACTTTC
O.michauxii                 CTG------- --TAGTTCCT TTTTTGCCAC AGGGGGCCAC GCTGACTTTC
O.subacaulis                CTG------- --TAGTTCCT TTTTTGCCAC AGGGGGCCAC GCTAACTTTC
O.teheranica                CTG------- --TAGTTCCT TTTTTGCCAC AGGGGGCCAC GCTGACTTTC
Alhagi maurorum             TTG------- --TAGTTCCT TTTTTGCCAC AAGGGGCCAC GCTAACTTTC
Caragana microphylla        TTG------- --TAGTTCCT TTTTTGCCAC AAGGGGCCAC GCTGACTTTC
C.korshinski                TTG------- --TAGTTCCT TTTTTGCCAC AAGGGGCCAC GCTGACTTTC
C.kozlowii                  TTG------- --TAGTTCCT TTTTTGCCAC AAGGGGCCAC GCTGACTTTC
C.rosea                     TTG------- --TAGTTCCC TTTTTGCCAC AAGGGGCCAC GCTGACTTTC
Tibetia liangshanensis      TTG------- --TAGTTCCT TTTTTGCCAC AAGGGGCCAC GCTTACTTTC
Gueldenstaedtia verna       TTG------- --TAGTTCCT TTTTTGCCAC AAGGGGCCAC GCTTACTTTC
Halimodendron halodendron   AGC------- --AAGGTCTA TATTTACCTC AGGGGAC-AT GCTT-CTTTC
Smirnowia turkestana        TTA------- --TAGTTCCT TTTTTGCCAC AAGGGGTCAC GCTGACTTTC
Eremosparton flaccidum      TTA------- --TAGTTCCT TTTTTGCCAC AAGGGGTCAC GCTGACTTTC
Colutea persica             TTC------- --TAGTTCCT TTTTTGCCAC AAGGGGTCAC GCTGACTTTC
C. triphylla                TTC------- --TAGTTCCT TTTTTGCCAC AAGGGGTCAC GGTGACTTTC
Sphaerophysa salsula        TTA------- --TAGTTCCT TTTTTGCCAC AAGGGGTCAC GCTGACTTTC
Podlechiella vogelii        TTC------- --TAGTTCCT TTTTTGCCAC AAGGGGTCAC GCTGACTTTC
Carmichaelia australis      TTC------- --TAGTTCCT TTTTTGCCAC AAGGGGTCAC GCTGACTTTC
Sutherlandia frutescens     TTC------- --TAGTTCCT TTTTTGCCAC AAGGGGTCAC GGTGACTTTC
Astragalus membranaceous    TTC------- --TAGTTCCT TTTTTGCCAC AAGGGGTCAC GCTGACTTTC
A.mongholicus               TTC------- --TAGTTCCT TTTTTGCCAC AAGGGGTCAC GCTGACTTTC
A.nakaianus                 TTC------- --TAGTTCCT TTTTTGCCAC AAGGGGTCAC GCTGACTTTC
A.macropelmatus             TTC------- --TAGTTCCT TTTTTGCCAC AAGGGGTCAC GCTGACTTTC
A.iranicus                  TTC------- --TAGTTCCT TTTTTGCCAC AAGGGGTCAC GCTGACTTTC
A.denudatus                 TTC------- --TAGTTCCT TTTTTGCCAC AAGGGGTCAC GCTGACTTTC
A.odoratus                  TTC------- --TAGTTCCT TTTTTGCCAC AAGGGGTCAC GCTGACTTTC
Oxytropis szovitsii         TTC------- --TAGTTCCT TTTTTGCCAC AAGGGGTCAC GCTGACTTTC
O.iranica                   TTC------- --TAGTTCCT TTTTTGCCAC AAGGGGTCAC GCTGACTTTC
O.kotschyana                TTC------- --TAGTTCCT TTTTTGCCAC AAGGGGTCAC GCTGACTTTC
Erophaca baetica            TTG------- --TAGTTCCT TTTTTGCCAC AAGGGGTCAC GCTGACTTTC
Wisteria floribunda         TTGAGCCTGA GCTAGTTCCT TTTTTGCCAC AAGGGGTCAC GATGACTATC
W. sinensis                 TTGAGCCTGA GCTAGTTCCT TTTTTGCCAC AAGGGGTCAC GATGACTATC
Glycyrrhiza lepidota        TTGAGCCTGA GCTAGTTCCT TTTTTGCCAC AAGGGGCCAC GATGACTTTC
G.uralensis                 TTGAGCCTGA GCTAGTTCCT TTTTTGCCAC AAGGGGCCAC GATGACTTTC
G.glabra                    TTGAGCCTGA GCTAGTTCCT TTTTTGCCAC AAGGGGCCAC GATGACTTTC
Meristotropis xathioides    TTGAGCCTGA GCTAGTTCCT TTTTTGCCAC AAGGGGCCAC GATGACTTTC
Robinia pseudoacacia        TTGACCCCGG GCTAATTTCT TTTTTGCCAC AAGGGGCTAC GATAATGGTC
Lotus japonicus             TTGATTCCGA GCTAATTCGT TTTTTGCCAC AAGGGGCTAC GATAACTGTT
Vicia alpestris             CGC------- --TCTTCTCT TTCTTCCCAC AAGGGGTCAG GGTGACTCGC
V.sativa                    TTG------- --TCATTCCT TTTTTGCCAC AAGGAGTCAC ACTGACTTTC
V.peregrina                 TTG------- --TCATTCCT TTTTTGCCAC AAGGAGTCAC ACTGACTTTC
V.sepium                    TTG------- --TCATTCCT TTTTTGCCAC AAGGAGTCAC GCTTACTTTC
V. faba                     TTG------- --TCATTCCT TTTTTGACAC AAGGAGTCAC GCTGACTTTC
```

```
V.canescens              TTG------- --TCATTCCT TTTTTGCCAC AAGGGGTCAC GCTGACTTTC
V.monantha               TTG------- --TCATTCCT TTTTTGCCAC AAGGGGTCAC GCTGACTTTC
V.tetrasperma            TTG------- --TCATTCCT TTTGTGCCAC AAGGGGTCAC GCTGACTTTC
V.narbonensis            TTG------- --TCATTCCT TTTTTGCCAC AAGGGGTCAC GCTTACTTTC
Lens culinaris           TTT------- --TCATTCCT TTTTTGCCAC AAGGGGTCAC GCTGACTTTC
L.orientalis             TTT------- --TCATTCCT TTTTTGCCAC AAGGGGTCAC CGTGACTTTC
V.ervilia                CTC------- --TCGTTTCT TTTTTGCCAC AAGGGGTCAC GCTCACTTTC
Galega officinalis       ---------- --TAGTTCCT TTTTTGCCAC AAGGGGTCAC GCTAACTTTC
Lathyrus palustris_K     TCGTC----- -CATCTCCTT ACAACACGGA AATATGTAAT ATTAATTCTC
L.palustris_H            TCGTC----- -CATCTCCTT ACAACACGGA AATATGTAAT ATTAATTCTC
L.davidii                TAGGC----- -CATATCCTT AAAACACGGA AATATCAGAT ATTAATTCTC
L.japonicus              TCGTC----- -CATATCCTT ACAACACGGA AATATGTAAT ATTAGTTCTC
L.ochroleucus            TCGTC----- -CATATCCTT ACAACACGGA AATATGTGAT ATTCATTCTG
L.littoralis             TCGTC----- -CATATCCTT ACAACACGGA AATATGTGAT ATTAATTCTC
L.venosus                TCGTC----- -CATATGCTT ACAACACGGA AATATGTGAT ATTAATTCTC
L.graminifolius          TCGTC----- -CATATCCTT ACAACACGGA AATATGTGAT ATTTATTCTC
L.pubescens              TTATC----- -CGTATCGAT AACCCAGTAA AATATATGAA GCATGCTCCT
L.sativus_KJ             TGGCT----- -CTTTGTGCT ACCAAATAAG ATTG---TTT CTCTGCTTAT
L.sativus_HM             TGGCT----- -CTTTGTGCT ACCAAATAAG ATTG---TTT CTCTGCTTAT
L.pseudocicera           TGGCT----- -CTTTGTGCC ACAAAATAAG ATTA---TTT ATCTGCTTGT
L.chloranthus            ---------- ---------- ---------- ---------- ----------
L.pratensis              ---------- ---------- ---------- ---------- ----------
L.odoratus_HM            ---------- ---------- ---------- ---------- ----------
L.odoratus_kJ            ---------- ---------- ---------- ---------- ----------
L.hirsutus               ---------- ---------- ---------- ---------- ----------
L.annuus                 ---------- ---------- ---------- ---------- ----------
L.cirrhosus              AGGGT----- -CTTCGATGT GCCAGTTAAT ACTACGCTTT TTCTGCTTGG
L.latifolius             AGGGT----- -CTTCGATGT GCCAGTTAAA ACTACGCTTT TTCTGCTTGG
L.tingitanus             GCGGT----- -CTATGTGCC ACCAGAGAAT AAGG---TAA GGGTGCTTGT
Pisum sativum_H          CAAATT---- -TTTACATTC ATT-TGGGAG GAAGTTTTTC GCTTACATTC
Pisum sativum_K          CAAATT---- -TTTACATTC ATT-TGGGAG GAAGTTTTTC GCTTACATTC
Pisum sativum_HG         CAAATT---- -TTTACATTC ATT-TGGGAG GAAGTTTTTC GCTTACATTC
Pisum fulvum             CAAATT---- -TTTACATTC ATT-TGGGAG GAAGTTTTTC GCTTACATTC
Vavilovia formosa        TCATTT---- -TTTAGATTC ATT-TGGGAG CAAGTTTTTC GCTTACATTC
L.clymenum               CGTTTAG--- -TTTGCAATT ATTGTGGGCG TACTTTTTTA GCTTTCATGC
L.ochrus                 CGTTTAG--- -TTTGCAATT ATTATGGGCG TACTTTTTTA GCTTTCATGC
```

```
                             651
Trifolium strictum          TAT---GGAA TCGCGGGTCT CTTTCTAAGT TTACATTGGT GGCTTCTTAT
T. glanduliferum            TAT---GGAA TCGCGGGTCT CTTTCTAAGT TTACATTGGT GGCTTCTTAT
T. boissieri                TAT---GGAA TCGCGGGTCT CTTTCTAAGT TTACATTGGT GGCTTCTTAT
T. aureum                   TAT---GGAA TCGCAGGTCT CTTTCTAAGT TTACATTGGT GGCTTCTTAT
T.meduseum                  TAT---GGAA TCGCGGGTCT CTTTCTAAGT TTACATTGGT GGCTTCTTAT
T.subterraneum              TAT---GGAA TCGCGGGTCT CTTTCTAAGT TTACATTGGT GGCTTCTTAT
T.pratense                  TAT---GGAA TCGCGGGTCT CTTTCTAAGT TTACATTGGT GGCTTCTTTT
T.hybridum                  TAT---GGAA TCGCAGGTCT CTTTCTAAGT TTACATTGGT GGCTTCTTAT
T.semipilosum               TAT---GGAA TCGCAGGTCT CTTTCTAAGT TTACATTGGT GGCTTCTTAT
T.occidentale               TAT---GGAA TCGCAGGTCT CTTTCTAAGT TTACATTGGT GGCTTCTTAT
T.repens                    TAT---GGAA TCGCAGGTCT CTTTCTAAGT TTACATTGGT GGCTTCTTAT
T.lupinaster                TAT---GGAA TCGCAGGTCT CTTTCTAAGT TTACATTGGT GGCTTCTTAT
Cicer oxyodon               TAT---GGAA TCGCGGGTCT CTTTCTAAGT TTTCATTGGT GGCTTCTCAT
C. chorrassanicum           TAT---GGAA TCGCGGGTCT CTTTCTAAGT TTTCATTGGT GGCTTCTCAT
C. arietinum                TAT---GGAA TCGCCGGTTT CTTTCTAAGT TTTCATTGGT GGCTTCTCAT
Medicago truncatula_KF      TAT---GGAA TCGCAGGTCT CTTTCTAAGT TTACATTGGT GGCTTCTAAT
M.truncatula_AC             TAT---GGAA TCGCAGGTCT CTTTCTAAGT TTACATTGGT GGCTTCTAAT
M.sativa_K                  TAT---GGAA TCGCAGGTCT CTTTCTAAGT TTACATTGGT GGCTTCTAAT
M.sativa                    TAT---GGAA TCGCAGGTCT CTTTCTAAGT TTACATTGGT GGCTTCTAAT
M.papillosa                 TAT---GGAA TCGCAGGTCT CTTTCTAAGT TTACATTGGT GGCTTCTAAT
M.hybrida                   TAT---GGAA TCGCAGGTCT CTTTCTAAGT TTACATTGGT GGCTTCTAAT
M.falcata                   TAT---GGAA TCGCAGGTCT CTTTCTAAGT TTACATTGGT GGCTTCTAAT
M.sativa_KU                 TAT---GGAA TCGCAGGTCT CTTTCTAAGT TTACATTGGT GGCTTCTAAT
Melilotus albus             TAT---GGAA TCGCAGGTCT CTTTTTAAGT TTACATTGGT GGCTTCTAAT
Hedysarum formosum          TAT---GGAA TCGCGGGTCT GTTTGTAAGT TTACATTGGT GGATTCTAAT
H.varium                    TAT---GGAA TCGCGGGTCT GTTTGTAAGT TTACATTGGT GGATTCTCAT
H.singarense                TAT---GGAA TCGCAGGTCT GTTTGTAAGT TTTCATTGGT GGATTCTCAT
H.minjanense                TAT---GGAA TCGCGGGTCT TTTTGTCAGT TTTCATTGGT GGATTCTAAT
Tavrniera glabra            TAT---GGAA TCGCGGGTCT GTTTGTAAGT TTCCATTGGT GGATTCTAAT
T.diffusa                   TAT---GGAA TCGCGGGTCT GTTTGTAAGT TTCCATTGGT GGATTCTAAT
Greuteria membranacea       TAT---GGAA TCGCGGGTCT ATTTGTAAGT TTTCATTGGT GGATTCTAAT
Corethrodendron scoparium   TAT---GGAA TCGCGGGTCT ATTTGTAAGT TTTCATTGGT GGATTCTAAT
Eversmannia subspinosa      TAT---GGAA TCGCGGGTCT ATTTGTAAGT TTTCATTGGT GGATTCTAAT
Onobrychis bungei           TAT---GGAA TCGCGGGTCT GTTTGTAAGT TTTCATTGGT GGATTCTAAT
O.cornuta                   TAT---GGAA TCGCGGGTCT GTTTGTAAGT TTTCATTGGT GGATTCTAAT
O.michauxii                 TAT---GGAA TCGCGGGTCT GTTTGTAAGT TTTCATTGGT GGATTCTAAT
O.subacaulis                TAT---GGAA TCGCGGGTCT GTTTGTAAGT TTTCATTGGT GGATTCTAAT
O.teheranica                TAT---GGAA TCGCGGGTCT GTTTGTAAGT TTTCATTGGT GGATTCTAAT
Alhagi maurorum             TAT---GGAA TCGCGGGTCT ATTTGTAAGT TTTCATTGGT GGATTCTCAT
Caragana microphylla        TAT---GGAA TCGCGGGTCT CTTTGTAAGT TTTCATTGGT GGATTCTAAT
C.korshinski                TAT---GGAA TCGCGGGTCT CTTTGTAAGT TTTCATTGGT GGATTCTAAT
C.kozlowii                  TAT---GGAA TCGCGGGTCT GTTTGTAAGT TTTCATTGGT GGATTCTAAT
C.rosea                     TAT---GGAA TCGCGGGTCT CTTTGTAAGT TTTCATTGGT GGATTCTAAT
Tibetia liangshanensis      TAT---GGAA TCGCGGGTCT CTTTGTAAGT TTTCATTGGT GGATTCTCAT
Gueldenstaedtia verna       TAT---GGAA TCGCGGGTCT CTTTGAAAGT TTTCATTGGT GGATTCTCAT
Halimodendron halodendron   TTT---AGAA TCGGTGGTTG ATCTTGTAGA TTCCATGGGA GATTGGATTT
Smirnowia turkestana        TAT---GGAA TCTCAGGTCT TTTTGTAAGT TTTCATTGGT GGCTTCTAAT
Eremosparton flaccidum      TAT---GGAA TCTCAGGTCT TTTTGTAAGT TTTCATTGGT GGCTTCTAAT
Colutea persica             TAT---GGAA TCTCAGGTCT TTTTGTAAGT TTTCATTGGT GGCTTTTAAT
C. triphylla                TAT---GGAA TCTCAGGTCT TTTTGTAAGT TTTCATTGGG GGCTTTTAAT
Sphaerophysa salsula        TAT---GGAA TCTCAGGTCT TTTTGTAAGT TTTCATTGGG GGGTTTTAAT
Podlechiella vogelii        TAT---GGAA TCTCAGGTCT TTTTGTAAGT TTTCATTGGT GGCTTCTAAT
Carmichaelia australis      TAT---GGAA TCTCAGGTCT TTTTGTAAGT TTTCATTGGT GGCTTCTAAT
Sutherlandia frutescens     TAT---GGAA TTTCCGGTCT TTTTGTAAGT TTTCATTGGT GGCTTCTAAT
Astragalus membranaceous    TAT---GGAA TCTCAGGTCT TTTTGTAAGT TTTCATTGGT GGCTTCTAAT
A.mongholicus               TAT---GGAA TCTCAGGTCT TTTTGTAAGT TTTCATTGGT GGCTTCTAAT
A.nakaianus                 TAT---GGAA TCTCAGGTCT TTTTGTAAGT TTTCATTGGT GGCTTCTAAT
A.macropelmatus             TAT---GGAA TCTCAGGTCT TTTTGTAAGT TTTCATTGGT GGCTTCTAAT
A.iranicus                  TAT---GGAA TCTCAGGTCT TTTTGTAAGT TTTCATTGGT GGCTTCTAAT
A.denudatus                 TAT---GGAA TCTCAGGTCT TTTTGTAAGT TTTCATTGGT GGCTTCTAAT
A.odoratus                  TAT---GGAA TCTCAGGTCT TTTTGTAAGT TTTCATTGGT GGCTTCTAAT
Oxytropis szovitsii         TAT---GGAA TCTCAGGTCT TTTTGTAAGT TTTCATTGGT GGCTTCTAAT
O.iranica                   TAT---GGAA TCTCAGGTCT TTTTGTAAGT TTTCATTGGT GGCTTCTAAT
O.kotschyana                TAC---GGAA TCTCAGGTCT TTTTGTAAGT TTTCATTGGT GGCTTCTAAT
Erophaca baetica            TAT---GGAA TCTCAGGTCT TTTTGTAAGT TTTCATTGGT GGCTTCTAAT
Wisteria floribunda         TAT---GGAA CCGCGGGTCT CTTTCTAAGT TTTTCTTGGT GGCTTCTAAT
W. sinensis                 TAT---GGAA CCGCGGGTCT CTTTCTAAGT TTTTCTTGGT GGCTTCTAAT
Glycyrrhiza lepidota        TAT---GGAA CCGCGGGTCT CTTTCTAAGT TTTTCTTGGT GGCTTCTAAT
G.uralensis                 TAT---GGAA CCGCGGGTCT CTTTCTAAGT TTTTCTTGGT GGCTTCTAAT
G.glabra                    TAT---GGAA CCGCGGGTCT CTTTCTAAGT TTTTCTTGGT GGCTTCTAAT
Meristotropis xathioides    TAT---GGAA CCGCGGGTCT CTTTCTAAGT TTTTCTTGGT GGCTTCTAAT
Robinia pseudoacacia        TAT---TCAA CCATGTTTCT ATTTTTAAGT TTTTCTTGGT GGCTTCTAAT
Lotus japonicus             TAT---GGGA CCGCGGGTCT ATTTGTTAGT TTGTTTTTGT GGCTTACCAT
Vicia alpestris             TAT---GGAA TGGCAGGCGT CCCTCTTAG- TCCCGCTGCA CCACCAAATC
V.sativa                    TAT---GGAA TTGCAGGTCT CTTTCTAAGT TTACATTGGT GGCTTCTCCT
V.peregrina                 TAT---GGAA TTGCAGGTCT CTTTCTAAGT TTACATTGGT GGCTTCTCCT
V.sepium                    TAT---GGAA TTGCAGGTCT CTTTCTAAGT TTACATTGGT GGCTTCTCAT
V. faba                     TAT---GGAA TTGCCGGTCT CTTTCTAAGT TTACATTGGT GGCTTCTAAT
```

```
V.canescens                TAT---GGAA TTGCGGGTCT CTTTCTAAGT TTACATTGGT GGCTTATAAT
V.monantha                 TAT---GGAA TTGCGGGTCT CTTTCTAAGT TTACATTGGT GGCTTCTAAT
V.tetrasperma              TAT---GGAA TTGCGGGTCT CTTTCTAAGT TTACATTGGT GGCTTCTAAT
V.narbonensis              TAT---GGAA TTGCAGGTCT CTTTCTAAGT TTACATTGGT GGCTTCTATT
Lens culinaris             TAT---GGAA TTGCAGGTCT CTTTCTAAGT TTACATTGGT GGCTTCTAAT
L.orientalis               TAT---GGAA TTGCAGGTCT CTTTCTAAGT TTCCCTTGGT GGCTTCTAAT
V.ervilia                  TAT---GGAA TCGCGGGTCT CCTTCTAAGT TTACATTGGT GGCTTCTAAT
Galega officinalis         TAT---GGAA TCGCGGGTCT CTTTCTAAGT TTCCATTGGT GGCTTCTAAT
Lathyrus palustris_K       CTT---GCAA TTATAAATAT ACTCATAACT ATAGGGGGTT CATTTCTATT
L.palustris_H              CTT---GCAG TTATAAATAT ACTCATAACT ATAGGGGGTT CATTTCTATT
L.davidii                  CTT---GCAA TTGGAAATAT ACTCATAAGT ATCGCAGGTT CCTTCTTATT
L.japonicus                CTT---GCAA TTCTAACTAT ATTCATAAGT ATAGCAGGTT CATTTGTATT
L.ochroleucus              CTT---GCAA TTATAAATAT ACTAATTGCA ATAGGAGGTT CATTTCTTTT
L.littoralis               CTT---GGAA TTATAAATAT ACTCATAAGT CTAGCAGGTT CATTTCTATT
L.venosus                  CTT---GCAA TTATAAATAT ACTAATTGGC ATAGGGGGTT CATTTCTTTT
L.graminifolius            TTT---GCAA TAATAAATAT ACTCCTGAGT CTAGGTGGTT CATTTCTTTT
L.pubescens                CTATTAGCAA TGCTAAGTCT CCTTGGAAGT TTAGCGTGTT CGCTTGTATT
L.sativus_KJ               CACT--GCAA TTGTCAGTAT CCTAAGCGGT ATCATGTTTC AATATATTAA
L.sativus_HM               CACT--GCAA TTGTCAGTAT CCTAAGCGGT ATCATGTTTC AATATATTAA
L.pseudocicera             CACT--GCAA TTGTAAGTAT CCTAATCGGT ATCATGTTTC AATATATTAA
L.chloranthus              ---------- ---------- ---------- ---------- ----------
L.pratensis                ---------- ---------- ---------- ---------- ----------
L.odoratus_HM              ---------- ---------- ---------- ---------- ----------
L.odoratus_kJ              ---------- ---------- ---------- ---------- ----------
L.hirsutus                 ---------- ---------- ---------- ---------- ----------
L.annuus                   ---------- ---------- ---------- ---------- ----------
L.cirrhosus                CAAA--GGAA TCGTAAGTCT CGTTTTAGGT TTTTTATTAG TATACAAAAC
L.latifolius               CAAA--GGAA TCGTAGGTAT CGTTTTAGGT TTTTTGTTGG TATACAAAAC
L.tingitanus               CTTT--GGAA TTGTAGGTAT CCTTCTAGGT TTCGGGTGTT TGCTTAATCT
Pisum sativum_H            AGAT--GAAG TTGTCCAGTG TGCCACGAG- ----AAGAAA GGGTCAGGCT
Pisum sativum_K            AGAT--GAAG TTGTCCAGTG TGCCACGAG- ----AAGAAA GGGTCAGGCT
Pisum sativum_HG           AGAT--GAAG TTGTCCAGTG TGCCACGAG- ----AAGAAA GGGTCAGGCT
Pisum fulvum               AGAT--GAAG TTGTCCAGTG TGCCACGAG- ----AAGAAA GGGTCAGGCT
Vavilovia formosa          AGAA--GAAG TTGTCCAGTG TGCCACGAG- ----AAATAA GGGTCAGGCT
L.clymenum                 CGGT--CAAC TGGATCTTCG TGCCACCAGC -CAGACAAAA GGGTCTGGCT
L.ochrus                   CGGT--CAAC TGGATCTTCG TGCCACCAG- ----ACAAAA GGGTCTGGCT
```

```
                             701
Trifolium strictum          TTTTTGGAAT GT-GGGGAGT GGTTATAATT TTTTCGATAA AAAAAATCGA
T. glanduliferum            TTTTTGGAAT GT-GGGGAGT GGTTATAATT TTTTCGATAA AAAAAATCGA
T. boissieri                TTTTTGGAAT GT-GGGGAGT GGTTATAATT TTTTCGATAA AAAAAATCGA
T. aureum                   TTTTTGGAAT GT-GGGGAGT GGTTATAATT TTTTCGATAA AAAAAATCGA
T.meduseum                  TTTTTGGAAT GT-GGGGAGT GGTTATAATT TTTTCGATAA AAAAAATCGA
T.subterraneum              TTTTTGGAAT GT-GGGGAGT GGTTATAATT TTTTCGATAA AAAAAATCGA
T.pratense                  TTTTTGGGAT GT-GGGAAGT GGCTATAATT TTTTCGATAA AAAAAATCGA
T.hybridum                  TTTTTGGAAT GT-GGGGAGT GGTTATAATT TTTTCGATAA AAAAAATCGA
T.semipilosum               TTTTTGGAAT GT-GGGGAGT GGTTATAATT TTTTCGATAA AAAAAATCGA
T.occidentale               TTTTTGGAAT GT-GGGGAGT GGTTATAATT TTTTCGATAA AAAAAATCGA
T.repens                    TTTTTGGAAT GT-GGGGAGT GGTTATAATT TTTTCGATAA AAAAAATCGA
T.lupinaster                TTTTTGGAAT GT-GGGGAGT GGCTATAATT TTTTCGATAA AAAAAATCGA
Cicer oxyodon               TTTTTGGAAT GT-GGGTAGT GGTTATAATT TTTTTGATAA AAAAAATAGA
C. chorrassanicum           TTTTTGGAAT GT-GGGTAGT GGTTATAATT TTTTTGATAA AAAAAATAGA
C. arietinum                TTTTTGGAAT GT-GGGTAGT GGTTATAATT TTTTTGATAA AAAAAATAGA
Medicago truncatula_KF      TTTTTGGAAT GT-GGGGAGT GGTTATAATT TTTTCGATAA AAAAAAGAGA
M.truncatula_AC             TTTTTGGAAT GT-GGGGAGT GGTTATAATT TTTTCGATAA AAAAAAGAGA
M.sativa_K                  TTTTTGGAAT GT-GGGGAGT GGTTATAATT TTTTCGATAA AAAAAATAGA
M.sativa                    TTTTTGGAAT GT-GGGGAGT GGTTATAATT TTTTCGATAA AAAAAATAGA
M.papillosa                 TTTTTGGAAT GT-GGGGAGT GGTTATAATT TTTTCGATAA AAAAAATAGA
M.hybrida                   TTTTTGGAAT GT-GGGGAGT GGTTATAATT TTTTCGATAA AAAAAATAGA
M.falcata                   TTTTTGGAAT GT-GGGGAGT GGTTATAATT TTTTCGATAA AAAAAATAGA
M.sativa_KU                 TTTTTGGAAT GT-GGGGAGT GGTTATAATT TTTTCGATAA AAAAAATAGA
Melilotus albus             TTTTTGGAAT GT-GGGGAGT GGTTATAATT TTTTCGATAA AAAAAATCGA
Hedysarum formosum          TTTTTGGAAT GT-GGGTAGT GGTTATAATC TTTATGATAA CCAAATTGGA
H.varium                    TTTTTGGAAT GT-GGGTAGT GGTTATAATC TTTATGATAA CCAAATTGGA
H.singarense                TTTTTGGAAT GT-GGGTAGT GGTTATAATC TTTATGATAA CCAAATAGGA
H.minjanense                TTTTTGGAAT GT-GGGTAGT GGTTATAATC TTTATGATAA CCAAATTGGA
Tavrniera glabra            TTTTTTTAAT GT-GGGTAGT GGTTATAATC TTTACGATAA CCAAATTGGA
T.diffusa                   TTTTTTTAAT GT-GGGTAGT GGTTATAATC TTTACGATAA CCAAATTGGA
Greuteria membranacea       TTTTTGGAAT GT-GGGTAGT GGTTATAATC TTTACGATAA CCAAATTGGA
Corethrodendron scoparium   TTTTTGGAAT GT-GGGTAGT GGTTATAATC TTTACGATAA CCAAATTGGA
Eversmannia subspinosa      TTTTTGGAAT GT-AGGTAGT GGTTATAATC TTTACGATAA CCAAATTGGA
Onobrychis bungei           TTTTTGGAAT GT-GGGTAGT GGTTATAATC TTTACGATAA CCAAATTGGA
O.cornuta                   TTTTTGGAAT GT-GGGTAGT GGTTATAATC TTTACGATAA CAAAATTGGA
O.michauxii                 TTTTTGGAAT GT-GGGTAGT GGTTATAATC TTTATGATAA CCAAATTGGA
O.subacaulis                TTTTTGGAAT GT-GGGTAGT GGTTATAATC TTTACGATAA CCAAATTGGA
O.teheranica                TTTTTGGAAT GT-GGGTAGT GGTTATAATC TTTACGATAA CCAAATTGGA
Alhagi maurorum             TTTTTGGAAT GT-GGGTAGT GGTTATAATC TTTACGATAA CCAAATTGGA
Caragana microphylla        TTTTTGGAAT GT-GGGTAGT GGTTATAATC TTTACGATAA TCAAATTGGA
C.korshinski                TTTTTGGAAT GT-GGGTAGT GGTTATAATC TTTACGATAA TCAAATTGGA
C.kozlowii                  TTTTTGTAAT GT-GGGTAGT GGTTATAATC TTTACGATAA TCAAATTGGA
C.rosea                     TTTTTGGAAT GT-GGGTAGT GGTTATAATC TTTACGATAA TCAAATTGGA
Tibetia liangshanensis      TTTTTGGAAT GT-GGGTAGT GGTTATAATC TTTACGATAG CAAAATTGGA
Gueldenstaedtia verna       TCTTTGGAAT GT-GGGTAGG GGTAATAATC TTTACAATAA -AAAATTGGA
Halimodendron halodendron   TATTTTTAAT GT-AACTCGT TTTTTTATTT ATATGGAGAT ACAACGTGCT
Smirnowia turkestana        TTTGTGGAAT GT-GGGTAGT GGTTATAATC TTTACGATAA AAAAAATGGA
Eremosparton flaccidum      TTTGTGGAAT GT-GGGTAGT GGTTATAATC TTTACGATAA AAAAAATGGA
Colutea persica             TTTGTGGAAT GT-GGGTAGT GGTTATAATC TTTACGATAA AAAAAATGGA
C. triphylla                TTTGTGGAAT GT-GGGTAGT GGTTATAATC TTTACGATAA AAAAAATGGA
Sphaerophysa salsula        TTTGTGGAAA GT-GGGTAGG GGTTATAATC TTTACGATAA AAAAAATGGA
Podlechiella vogelii        TTTGTGGAAT GT-GGGTAGT GGTTATAATC TTTACGATAA AAAAAATGGA
Carmichaelia australis      TTTGTGGAAT GT-GGGTAGT GGTTATAATC TTTACGATAA AAAAAATGGA
Sutherlandia frutescens     TTTGTGGAAT GT-GGGTAGT GGTTATAATC TTTACGATAA AAAAAATGGA
Astragalus membranaceous    TTTGTGGAAT GT-GGGTAGT GGTTATAATC TTTACGATAA AAAAAATGGA
A.mongholicus               TTTGTGGAAT GT-GGGTAGT GGTTATAATC TTTACGATAA AAAAAATGGA
A.nakaianus                 TTTGTGGAAT GT-GGGTAGT GGTTATAATC TTTACGATAA AAAAAATGGA
A.macropelmatus             TTTGTGGAAT GT-GGGTAGT GGTTATAATC TTTACGATAA AAAAAATGGA
A.iranicus                  TTTGTGGAAT GT-GGGTAGT GGTTATAATC TTTACGATAA AAAAAATGGA
A.denudatus                 TTTGTGGAAT GT-GGGTAGT GGTTATAATC TTTACGATAA AAAAAATGGA
A.odoratus                  TTTGTGGAAT GT-GGGTAGT GGTTATAATC TTTACGATAA AAAAAATGGA
Oxytropis szovitsii         TATGTGGAAT GT-GGGTAGT GGTTATAACC TTTACGATAA AAAAAATGGA
O.iranica                   TATGTGGAAT GT-GGGTAGT GGTTATAACC TTTACGATAA AAAAAATGGA
O.kotschyana                TATGTGGAAT GT-GGGGAGT GGTTATAATC TTTACGATAA AAAAAATGGA
Erophaca baetica            TTTGTGGAAT GT-GGGTAGT GGTTATAATC TTTACGATAA AAAAAATGGA
Wisteria floribunda         TTTTTGGAAT GT-GGGTAGT GGTTATGATT TTTTTGATAA AAAAAATGGA
W. sinensis                 TTTTTGGAAT GT-GGGTAGT GGTTATGATT TTTTTGATAA AAAAAATGGA
Glycyrrhiza lepidota        TTTTTGGAAT GT-GGGTAGT GGTTATGATT TTTTCGATAA AAAAAATAAA
G.uralensis                 TTTTTGGAAT GT-GGGTAGT GGTTATGATT TTTTCGATAA AAAAAATAAA
G.glabra                    TTTTTGGAAT GT-GGGTAGT GGTTATGATT TTTTCGATAA AAAAAATAAA
Meristotropis xathioides    TTTTTGGAAT GT-GGGTAGT GGTTATGATT TTTTCGATAA AAAAAATAAA
Robinia pseudoacacia        TTTTTGGAAT GT-GGGTAGT GGCTATGATC TTTTCGATAA AAAAACTAGA
Lotus japonicus             TTTTTGGAAT GT-GGGCAGT GGTTATGATC TTTTCGATAA AAAAAGAGGA
Vicia alpestris             TGATTGCACC ACCAGGGAGT GCTTATAATA ATTACGACCC ACCAAATGAA
V.sativa                    TTTTTGGAAT GT-GGGGAGT GGCTATAATT TTTTCGATAA AAAAAATCAA
V.peregrina                 TTTTTGGAAT GT-GGGGAGT GGCTATAATT TTTTCGATAA AAAAAATAAA
V.sepium                    TTTTTGGAAT GT-GGGGAGT GGCTATAATT TTTTCGATAA AAAAAATCGA
V. faba                     TTTTTGGAAT GT-GGGGAGT GGCTATAATT TTTTCGATAA AAAAAATCGA
```

```
V.canescens                TTTTTGGAAT GT-GGGGAGT GGCTATAATT TTTTCGATAA AAAAAATAGA
V.monantha                 TTTTTGGAAT GT-GGGGAGT GGCTATAATT TTTTCGATAA AAAAAATAGA
V.tetrasperma              TTTTTGGAAT GT-CGGGAGT GGCTATAATT TTTTCGATAA AAAAAATAGA
V.narbonensis              TTTTTGGAAT GT-GGGGAGT GGCTATAATT TTTTCGATAA AAAAAATAGA
Lens culinaris             TTTTTGGAAT GT-GGGGAGT GGCTATAATT TTTTCGATAA AAAAAATAGA
L.orientalis               TTTTTGGAAT GT-GGGGAGT GGCTATAATT TTTTCGATAA AAAAAATAGA
V.ervilia                  TTTTTGGAAT GT-AGGGAGT GGTTATAATT TTTTCGATAA AAAAAATCGA
Galega officinalis         TTTTTGGAAT GT-GGGTAGT GGTTATAATT TTTTTGATAA AAAAAATAGA
Lathyrus palustris_K       TTATTGGAAT AA-AGGGGGT GGCTCAAATT TTTTTGATAA AAAAAATGGA
L.palustris_H              TTATTGGAAT AA-AGGGGGT GGATCCAATT TTTTTGATAA AAAAAATGGA
L.davidii                  CTATTATAAT AG-AGGAGCT GGATATAATT TCTTCGATAG AGAGAAGGGT
L.japonicus                TTATTGGAAC AG-GGGCGGT GGCTACAATT TGTTCGATAA AAAAAATGGA
L.ochroleucus              TTATTGGAAC AA-GGGGTCC GGCTTCAATT TTTTCGATAA AAAAAATGGC
L.littoralis               CTATTGGAAT CG-GGGAGGT GGCTCCAATT TTTTCGATAA AAAAAATGAA
L.venosus                  TTATTGGAAC AA-GGGCAGC GGCGCCAATT TTTTCGATAA AAAAAATGGT
L.graminifolius            TTATTGGAAT AC-GGGCGCC GGCTCCAATT TTTTCGATAA AAAAAAAGAT
L.pubescens                TTATTGGAAT GT-AGGAAGC GGGTATAATC TATACCATAA AGCAAAAGGA
L.sativus_KJ               TTATTGGACT GT-AATTGGC GGAACTAATT TTTTCGATAA AAGAAAAGGA
L.sativus_HM               TTATTGGACT GT-AATTGGC GGAACTAATT TTTTCGATAA AAGAAAAGGA
L.pseudocicera             TTATTGCACT GT-AACTGGC GGAACTAATT TTTTCGATAA AGAAGAAGGA
L.chloranthus              ---------- ---------- ---------- ---------- ----------
L.pratensis                ---------- ---------- ---------- ---------- ----------
L.odoratus_HM              ---------- ---------- ---------- ---------- ----------
L.odoratus_kJ              ---------- ---------- ---------- ---------- ----------
L.hirsutus                 ---------- ---------- ---------- ---------- ----------
L.annuus                   ---------- ---------- ---------- ---------- ----------
L.cirrhosus                CTATTGCAAT AA-TAACAGC GGAACTAATT GTTTTGATAA AACAAAAGGA
L.latifolius               CTATTCCAAT AG-TAACAGC GGAACTAATT GTTTTGATAA AACAAAAGGA
L.tingitanus               TTATTGCAAT GT-GGGGAGT GGCTCTAATT TTTTCGATAA AAAAAATGGA
Pisum sativum_H            GCTTTTGTAT GC-----AAT TGTAAAGATC TTTCTAACGT --TATATCGA
Pisum sativum_K            GCTTTTGTAT GC-----AAT TGTAAAGATC TTTCTAACGT --TATATCGA
Pisum sativum_HG           GCTTTTGTAT GC-----AAT TGTAAAGATC TTTCTAACGT --TATATCGA
Pisum fulvum               GCTTTTGTAT GC-----AAT TGTAAAGATC TTTCTAACGT --TATATCGA
Vavilovia formosa          GCTTGTGTAT GC-----AAT TGTACATATC CTTGGATTTT --TATATAAT
L.clymenum                 GCTTTTCTAT GC-----AAT TGGAAGTATC GCTCTATGTT --TATGGTGT
L.ochrus                   GCTTTTCTAT GC-----AAT TGGAAGTATC GCTCTATGTT --TATGGTGT
```

```
                             751
Trifolium strictum          ATGGTGTGTT TTTTTCGTTA CGGATTT--- ---CCTGGAA CATATCGTCG
T. glanduliferum            ATGGTGTGTT TTTTTCGTTA CGGATTT--- ---CCTGGAA CATATCGTCG
T. boissieri                ATGGTGTGTT TTTTTCGTTA CGGATTT--- ---CCTGGAA CATATCGTCG
T. aureum                   ATGGTGTGTT TTTTTCGTTA CGGATTT--- ---CCTGGAA CATATCGTCG
T.meduseum                  ATGGTTTGTT TTTTTCGTTA CGGATTT--- ---CCTGGAA CATATCGTCG
T.subterraneum              ATGGTTTGTT TTTTTCGTTA CGGATTT--- ---CCTGGAA CATATCGTCG
T.pratense                  ATGGTTTGTT TTTTTCGTTA CGGATTT--- ---CCCGGAA CATATCGTCG
T.hybridum                  ATGATTTGTT TTTTTCGTTA TGGATTT--- ---CCTGGAA CATATCGTCG
T.semipilosum               ATGGTTTGTT TTTTTCGTTA TGGATTT--- ---CCTGGAA CATATCGTCG
T.occidentale               ATGGTTTGTT TTTTTCGTTA CGGATTT--- ---CCTGGAA CATATCGTCG
T.repens                    ATGGTTTGTT TTTTTCGTTA CGGATTT--- ---CCTGGAA CATATCGTCG
T.lupinaster                ATGGTGTGTT TTTTTCGTTA CGGATTT--- ---CCTGGAA CATATCGTCG
Cicer oxyodon               ATGGTGTGTT TTTTTCGTTA CGGATTT--- ---CCTGGAA CATATCGTCG
C. chorrassanicum           ATGGTGTGTT TTTTTCGTTA CGGATTT--- ---CCTGGAA CATATCGCCG
C. arietinum                ATGGTGTGTT TTTTTCGTTA CGGATTT--- ---CCTGGAA CATATCGTCG
Medicago truncatula_KF      ATGGTGTGTT TTTTTCGTTA CGGATTT--- ---CCTGGAA CATATCGTCG
M.truncatula_AC             ATGGTGTGTT TTTTTCGTTA CGGATTT--- ---CCTGGAA CATATCGTCG
M.sativa_K                  ATGGTGTGTT TTTTTCGTTA CGGATTT--- ---CCTGGAA CATATCGTCG
M.sativa                    ATGGTGTGTT TTTTTCGTTA CGGATTT--- ---CCTGGAA CATATCGTCG
M.papillosa                 ATGGTGTGTT TTTTTCGTTA CGGATTT--- ---CCTGGAA CATATCGTCG
M.hybrida                   ATGGTGTGTT TTTTTCGTTA CGGATTT--- ---CCTGGAA CATATCGTCG
M.falcata                   ATGGTGTGTT TTTTTCGTTA CGGATTT--- ---CCTGGAA CATATCGTCG
M.sativa_KU                 ATGGTGTGTT TTTTTCGTTA CGGATTT--- ---CCTGGAA CATATCGTCG
Melilotus albus             ATGGTGTGTT TTTTTCGTTA CGGATTT--- ---CCTGGAA CATATCGTCG
Hedysarum formosum          ATGGTGTGGC TTTGTCGTTA CGGATTT--- ---CCTGGAG AAAATCGTTA
H.varium                    ATGGTGTGGC TTTGTCGTTA CGGATTT--- ---CCTGGAG AAAATCGTTA
H.singarense                ATGGTGTGGC TTTGTCGTTA CGGATTT--- ---CCTGGAG AAAATCGTTA
H.minjanense                ATGGTGTGGC TTTGTCGTTA CGGATTT--- ---CCTGGAG AAAATCGTTA
Tavrniera glabra            ATGGTGTGGC TTTGTCGTTA CGGATTT--- ---CCTGGAG AAAATCGTTA
T.diffusa                   ATGGTGTGGC TTTGTCGTTA CGGATTT--- ---CCTGGAG AAAATCGTTA
Greuteria membranacea       ATGGTGTGGC TTTGTCGTTA CGGATTT--- ---CCTGGAG AAAATCGTTA
Corethrodendron scoparium   ATGGTGTGGC TTTGTCGTTA CGGATTT--- ---CCTGGAG AAAATCGTTA
Eversmannia subspinosa      ATGGTGTGGC TTTGTCGTTA CGGATTT--- ---CCTGGAG AAAATCGTTA
Onobrychis bungei           ATGGTGTGGC TTTGTCGTTA CGGATTT--- ---CCTGGAG AAAATCGTTG
O.cornuta                   ATGGTGTGGC TTTGTCGTTA CGGATTT--- ---CCTGGAG AAAATCGTTC
O.michauxii                 ATGGTGTGGC TTTGTCGTTA CGGATTT--- ---CCGGGAG AAAATCGTTG
O.subacaulis                ATGGTGTGGC TTTGTCGTTA CGGATTT--- ---CCGGGAG AAAATCGTTA
O.teheranica                ATGGTGTGGC TTTGTCGTTA CGGATTT--- ---CCGGGAG AAAATCGTTG
Alhagi maurorum             ATGGTGTGGC TTTGTCGTTA CGGATTT--- ---CCTGGAG AAAATCGTTA
Caragana microphylla        ATGGTGTGGT TTTTTCGTTA CGGATTT--- ---CCTGGAG AAAATCGCTG
C.korshinski                ATGGTGTGGT TTTTTCGTTA CGGATTT--- ---CCTGGAG AAAATCGCTG
C.kozlowii                  ATGGTGTGGT TTTTTCGTTA CGGATTT--- ---CCTGGAG AAAATCGTTG
C.rosea                     ATGGTGTGGT TTTTTCGTTA CGGATTT--- ---CCTGGAG AAAATCGTTG
Tibetia liangshanensis      ATGGTGTGGT TATTTCGTTA CGGATTT--- ---CCTGGAG AAAATCGTTG
Gueldenstaedtia verna       ATGGTGCGGT -ATTTCGT-A CGGATTT--- ---CCTGGCG AAAATCGT-G
Halimodendron halodendron   GTGTTGTGAA TTATTTATTA ATGGTTT--- ---TCCAGAG AAATTTTTCG
Smirnowia turkestana        ATGGTGTGGT TTTTTCGTTA CGGATTT--- ---CCCGGAG AAAATCGTCG
Eremosparton flaccidum      ATGGTGTGGT TTTTTCGTTA CGGATTT--- ---CCCGGAG AAAATCGTCG
Colutea persica             ATGGTGTGGT TTTTTCGTTA CGGATTT--- ---CCCGGAG AAAATCGTCG
C. triphylla                ATGGGGTGGT TTTTTCGGTA CGGATTT--- ---CCCGGAG AAAATCGTCG
Sphaerophysa salsula        ATGGTGTGGT TTTTTCGTTA CGGGTTT--- ---CCCCGGG AAAATTGTCG
Podlechiella vogelii        ATGGTGTGGT TTTTTCGTTA CGGATTT--- ---CCTGGAG AAAATCGTCG
Carmichaelia australis      ATGGTGTGGT TTTTTCGTTA CGGATTT--- ---CCCGGAG AAAATCGTCG
Sutherlandia frutescens     ATGGTATGGT TTTTTCGTTA CGGATTT--- ---CCCGGAG AAAATCGTCG
Astragalus membranaceous    ATGGTGCGGT TTTTTCGTTA CGGATTT--- ---CCCGGAG AAAATCGTCG
A.mongholicus               ATGGTGCGGT TTTTTCGTTA CGGATTT--- ---CCCGGAG AAAATCGTCG
A.nakaianus                 ATGGTGCGGT TTTTTCGTTA CGGATTT--- ---CCCGGAG AAAATCGTCG
A.macropelmatus             ATGGTGCGGT TTTTTCGTTA CGGATTT--- ---CCCGGAG AAAATCGTCG
A.iranicus                  ATGGTGCGTT TTTTTCGTTA CGGATTT--- ---CCCGGAG AAAATCGTCG
A.denudatus                 ATGGTGCGTT TTTTTCGTTA CGGATTT--- ---CCCGGAG AAAATCGTCG
A.odoratus                  ATGGTGCGTT TTTTTCGTTA CGGATTT--- ---CCCGGAG AAAATCGTCG
Oxytropis szovitsii         ATGGTGCGAT TTTTTCGTTA CGGATTT--- ---CCCGGAG AAAATCGTCG
O.iranica                   ATGGTGCGGT TTTTTCGTTA CGGATTT--- ---CCCGGAG AAAATCGTCG
O.kotschyana                ATGGTGCGGT TTTTTCGTTA CGGATTT--- ---CCCGGAG AAAATCGTCG
Erophaca baetica            ATGGTGTGGT TTTTTCGTTA CGGATTT--- ---CCTGGAG AAAATCGTCG
Wisteria floribunda         ATGGTGTCTT TTTTTCGTTA CGGATTT--- ---CCTGGAA AAAATCGTCG
W. sinensis                 ATGGTGTCTT TTTTTCGTTA CGGATTT--- ---CCTGGAA AAAATCGTCG
Glycyrrhiza lepidota        ATGGTGTGTT TTTTTCGTTA CGGATTT--- ---CCTGGAA AAAATCGTCG
G.uralensis                 ATGGTGTGTT TTTTTCGTTA CGGATTT--- ---CCTGGAA AAAATCGTCG
G.glabra                    ATGGTGTGTT TTTTTCGTTA CGGATTT--- ---CCTGGAA AAAATCGTCG
Meristotropis xathioides    ATGGTGTGTT TTTTTCGTTA CGGATTT--- ---CCTGGAA AAAATCGTCG
Robinia pseudoacacia        ACAGTGCTCT TTTTTCGTTA TGGATTT--- ---CCTGGAA AAAATCGTCG
Lotus japonicus             ATAGTGTGCA TTTTTCGTTA CGGATTT--- ---CCTGGAA AAAATCGTCG
Vicia alpestris             ATGGTATATT CTTCTCCACA TCCAGTT--- ---CTTCCAA CATTTTCTTG
V.sativa                    ATGGTATGTT TTTTTCGTTA CGGATTT--- ---CCTGGAA CATATCGTCG
V.peregrina                 ATGGTATGTT TTTTTCGTTA CGGATTT--- ---CCTGGAA CATATCGTCG
V.sepium                    ATGGTATGTT TTTTTCGTTA CGGATTT--- ---CCTGGAA CATATCGTCG
V. faba                     ATGGTATGTT TTTTTCGTTA CGGATTT--- ---CCTGGAA CATATCGTCG
```

V.canescens	ATGGTATGTT	TTTTTCGTTA	CGGATTT---	---CCTGGAA	CATATCGTCG
V.monantha	ATGGTATGTT	TTTTTCGTTA	CGGATTT---	---CCTGGAA	CATATCGTCG
V.tetrasperma	ATGGTATGTT	TTGTTCGCTA	CGGATTT---	---CCTGGAA	CATATCGTCG
V.narbonensis	ATGGTATGTT	TTTTTCGTTA	CGGATTT---	---CCTGGAA	CATATCGTCG
Lens culinaris	ATGGTATGTT	TTTTTCGTTA	CGGATTT---	---CCTGGAA	CATATCGTCG
L.orientalis	ATGGTATGTT	TTTTTCGGTA	CGGATTT---	---CCTGGAA	CATATTGTTG
V.ervilia	ATGGTATGTT	TTTTTCGTTA	CGGATTT---	---CCTGGAA	CAAATCGTCG
Galega officinalis	ACGGTATGTT	TTTTTCGTTA	CGGATTT---	---CCTGGAA	CATATCGTCG
Lathyrus palustris_K	CAGGTTTATA	TTACTCGTTA	CGGAATC---	---CCTGGAG	AGGGTCGTTT
L.palustris_H	CGGATTTATA	TTACTCGTTA	CGGAATC---	---CCTGGAG	AGGGTCGTTT
L.davidii	TATATCCATA	TTACTCGTTA	CGGAATC---	---CCTGGAG	AAGGTCGTTT
L.japonicus	CGTGTTTATA	TTACTCGTTA	CGGAATC---	---CCTGGAG	AAGGTCGTTT
L.ochroleucus	AGGGTCTATA	TTACTCGTTA	CGGAATC---	---CCTGGAG	AAGGTCGTTT
L.littoralis	CGCGTCTATC	TTACTCGTTA	CGGAATC---	---CCTGGAG	AAGGTCGTTT
L.venosus	AAGGTCTATA	TTACTCGTTA	CGGAATC---	---CCTGGAG	AAGGTCGTTT
L.graminifolius	GTAATCTATA	TTACTCGTTA	CGGAATC---	---CCTGGAG	AAGGTCGTTT
L.pubescens	ATGGTATATA	TTTCTCGTAA	CGGATTT---	---CCTGGAA	AAGGTCGTTT
L.sativus_KJ	GTGCTATATA	TTACTCGTTA	CAGACTTCCT	ATTCCTGGAA	TAAGTAGCTC
L.sativus_HM	GTGCTATATA	TTACTCGTTA	CAGACTTCCT	ATTCCTGGAA	TAAGTAGCTC
L.pseudocicera	GTGCTATATA	TTACTCGTTA	CGGACTTCCT	ATTCCTGGAA	TAAGTCGCTC
L.chloranthus	----------	----------	----------	----------	----------
L.pratensis	----------	----------	----------	----------	----------
L.odoratus_HM	----------	----------	----------	----------	----------
L.odoratus_kJ	----------	----------	----------	----------	----------
L.hirsutus	----------	----------	----------	----------	----------
L.annuus	----------	----------	----------	----------	----------
L.cirrhosus	ATGCTATATA	TTATTCGTTA	CGAATTCTCC	TTCCCTGGAA	AAACTAGTCG
L.latifolius	ATGCTATATA	TTATTCGTTA	CGGATTCCCC	TTCCCTGGAA	AAACTAGTCG
L.tingitanus	ATGGTATCTA	TTACTCGTTA	CGGACTT---	---CCTGGAA	AAGATCGTTG
Pisum sativum_H	ATGAGATTTA	GTTGTCGTTC	CGGCTTT---	---TCTGGAA	GAGATCGTTG
Pisum sativum_K	ATGAGATTTA	GTTGTCGTTC	CGGCTTT---	---TCTGGAA	GAGATCGTTG
Pisum sativum_HG	ATGAGATTTA	GTTGTCGTTC	CGGCTTT---	---TCTGGAA	GAGATCGTTG
Pisum fulvum	ATGAGATTTA	GTTGTCGTTC	CGGCTTT---	---TCTGGAA	GAGATCGTTG
Vavilovia formosa	ATAACATTTA	TTTCTCGTTC	CGGCTTT---	---CCTGGAA	GAGATCGTTG
L.clymenum	TTGGTTCTAA	TTTATTG---	---CAAT---	---CTGCGGA	GTGGCTTTAA
L.ochrus	TTGGTTCTAA	TTTATTG---	---CAAT---	---CTGCGGA	GTGGCTTTAA

```
                            801
Trifolium strictum         TATTTTTCTC CGAGTTCGTA TGGAAGATAT TCAGTCCCTC ATACTACAAG
T. glanduliferum           TATTTTTCTC CGAGTTCGTA TGGAAGATAT TCAGTCCCTC ATACTACAAG
T. boissieri               TATTTTTCTC CGAGTTCGTA TGGAAGATAT TCAGTCCCTC ATACTACAAG
T. aureum                  TATTTTTCTC CGAGTTCGTA TGGAAGATAT TCAGTCCCTC ATACTACAAG
T.meduseum                 TATTTTTCTC CGAGTTCGTA TGGAAGATAT TCAGTCCCTC ATACTACAAG
T.subterraneum             TATTTTTCTC CGAGTTCGTA TGGCAGATAT TCAGTCCCTC ATACTACAAG
T.pratense                 TATTTTTCTC CGAGTTCGTA TGGAAGATAT TCAGTCCCTC GTACTACAAG
T.hybridum                 TATTTTTCTC CGAGTTCGTA TGGAAGATAT TCAGTCCCTC ATACTACAAG
T.semipilosum              TATTTTTCTC CGAGTTCGTA TAGAAGATAT TCAGTCCCTC ATACTACAAG
T.occidentale              TATTTTTCTC CGAGTTCGTA TGGAAGATAT TCAGTCCCTC ATACTACAAG
T.repens                   TATTTTTCTC CGAGTTCGTA TGGAAGATAT TCAGTCCCTC ATACTACAAG
T.lupinaster               TATTTTTCTC CGAGTTCGTA TGGAAGATAT TCAGTCCCTC ATACTACAAG
Cicer oxyodon              TATTTTTCTC CGAGTTCGTA TGGAAGATAT TCAGTCCCTC ATACTACAAG
C. chorrassanicum          TATTTTTCTC CGAGTTCGTA TAGAAAATAT TCAGTCCCTC ATACTACAAG
C. arietinum               TATTTTTCTC CGAGTTCGTA TGGAAGATAT TCAGTCCCTC ATACTACAAG
Medicago truncatula_KF     TATTTTTCTC AGAGTTCGTA TGGAAGATAT TCAGTCCCTC ATACTACAAG
M.truncatula_AC            TATTTTTCTC CGAGTTCGTA TGGAAGATAT TCAGTCCCTC ATACTACAAG
M.sativa_K                 TATTTTTCTC CGAGTTCGTA TGGAAGATAT TCAGTCTCTC ATACTACAAG
M.sativa                   TATTTTTCTC CGAGTTCGTA TGGAAGATAT TCAGTCTCTC ATACTACAAG
M.papillosa                TATTTTTCTC CGAGTTCGTA TGGAAGATAT TCAGTCTCTC ATACTACAAG
M.hybrida                  TATTTTTCTC CGAGTTCGTA TGGAAGATAT TCAGTCTCTC ATACTACAAG
M.falcata                  TATTTTTCTC CGAGTTCGTA TGGAAGATAT TCAGTCTCTC ATACTACAAG
M.sativa_KU                TATTTTTCTC CGAGTTCGTA TGGAAGATAT TCAGTCTCTC ATACTACAAG
Melilotus albus            TATTTTTCTC CGAGTTCGTA TGGAAGATAT TCAGTCCCTC ATACTACAAG
Hedysarum formosum         TATTCTTTCC CAAGTTCGTG TGGAAGATAT TTTGGCCCTT CAATTTTATG
H.varium                   TATTCTTTCC CAAGTTCGTG TGGAAGATAT TTTGGCCCTT CAATTTTATG
H.singarense               TATTCTTTCC CAAGTTCGTG TGGAAGATAT TTTGGCCCTT CAATTTTATG
H.minjanense               TATTCTTTCC CAAGTTCGTG TGGAAGATAT TTTGGCCCTT CAATTTTATG
Tavrniera glabra           TATTCTTTCC CAAGTTCGTG TGGAAGATAT TTTGGCCCTT CAATTTTATG
T.diffusa                  TATTCTTTCC CAAGTTCGTG TGGAAGATAT TTTGGCCCTT CAATTTTATG
Greuteria membranacea      TATTCTTTCC CAAGTTCGTG TGGAAGATAT TTTGGCCCTT CAATTTTACG
Corethrodendron scoparium  TATTCTTTCC CAAGTTCGTG TGGAAGATAT TTTGGCCCTT CAATTTTACG
Eversmannia subspinosa     TATTCTTTCC CAAGTTCGTG TAGAAGATAT TTTGGCCCTT CAATTTTACG
Onobrychis bungei          TATTCTTTCC CAAGTTCGTG TGGAAGATAT TTTGGCCCTT CAATTTTATG
O.cornuta                  TATTCTTTCC CAAGTTCGTG TGGAAGATAT TTTGGCCCTT CAATTTTACG
O.michauxii                TATTCTTTCC CAAGTTCGTG TAGAAGATAT TTTGGCCCTT CAATTTTATG
O.subacaulis               TATTCTTTCC CAAGTTCGTG TGGAAGATAT TTTGGCTCTT CAATTTTACG
O.teheranica               TATTCTTTCC CAAGTTCGTG TAGAAGATAT TCTGGCTCTT CAATTTTACG
Alhagi maurorum            TATTCTTTCC CACGTTCGTG TGGAAGATAT TCTGGCCCTC CAATTTTACT
Caragana microphylla       TATTCTTTCC CACGTCCGTG TAGAAGATAT TCTGGCCCTC CAATTTTACG
C.korshinski               TATTCTTTCC CACGTCCGTG TAGAAGATAT TCTGGCCCTC CAATTTTACG
C.kozlowii                 TATTCTTTCC CACGTCCGTG TAGAAGATAT TCTGGCCCTC CAATTTTACG
C.rosea                    TATTCTTTCC CACGTCCGTG TAGAAGATAT TCTGGCCCTC CAATTTTACG
Tibetia liangshanensis     TATTCTTTCC CACGTCCGTG TGGAAGATAT TCTAGCCCTC CAATTTTACG
Gueldenstaedtia verna      AATTCTTTCC CACGTTCGTG TGAAAAATAT TCTAGCCCTC CAATTTTACG
Halimodendron halodendron  TATTCTTCCC CACCTCCACG TTGGAGATAA TCAGCCCCTC CAGCAATTTG
Smirnowia turkestana       TATTCTTTCC AAAGTCCGTG TGGAAGATAT TCTGGCCCTC CAATTTTTCG
Eremosparton flaccidum     TATTCTTTCC AAAGTCCGTG TGGAAGATAT TCTGGCCCTC CAATTTTTCG
Colutea persica            TATTCTTTCC AAAGTCCGTG TGGAAGATAT TCTGGCCCTC CAATTTTTCG
C. triphylla               GATTCTTTCC AAAGTCCGTG TGGAAGATAT TCTGGCCCTC CAATTTTTCG
Sphaerophysa salsula       TATTCTTTCC CAAGTCCGTG TGGAAGATAT TCTGGCCCTC CAAATTTTCG
Podlechiella vogelii       TATTCTTTCC AAAGTCCGTG TGGAAGATAT TTTGGCCCTC CAATTTTTCG
Carmichaelia australis     TATTCTTTCC AAAGTCCGTG TGGAAGATAT TCTGGCCCTC CAATTTTTCG
Sutherlandia frutescens    TATTCTTTCC AAAGTCCGTG TGGAAGATAT TCTGGCCCTT CAATTTTTCC
Astragalus membranaceous   TATTCTTTCC AAAGTCCGTG TGGAAGATAT TCTGGCCCTC CAATTTTTCG
A.mongholicus              TATTCTTTCC AAAGTCCGTG TGGAAGATAT TCTGGCCCTC CAATTTTTCG
A.nakaianus                TATTCTTTCC AAAGTCCGTG TGGAAGATAT TCTGGCCCTC CAATTTTTCG
A.macropelmatus            TATTCTTTCC AAAGTCCGTG TGGAAGATAT TCTGGCCCTC CAATTTTTCG
A.iranicus                 TATTCTTTCC AAAGTCCGTG TGGAAGATAT TCTGGCCCTC CAATTTTTCG
A.denudatus                TATTCTTTCC AAAGTCCGTG TGGAAGATAT TCTGGCCCTC CAATTTTTCG
A.odoratus                 TATTCTTTCC AAAGTCCGTG TGGAAGATAT TCTGGCCCTC CAATTTTTCG
Oxytropis szovitsii        TATTATTTCC AAAGTACGTG TGGAAGATAT TCTGGCCCTC CAATTTTTCG
O.iranica                  TATTATTTCC AAAGTACGTG TGGAAGATAT TCTGGCCCTC CAATTTTTCG
O.kotschyana               TATTATTTCC AAAGTACGTG TGGAAGATAT TCTGGCCCTC CAATTTTTCG
Erophaca baetica           TATTCTTTCC AAAGTCCGTG TGGAAGATAT TCTGGCCCTC CAATTTTTCG
Wisteria floribunda        TATTTTTCTC CGAGTCCGTA TGGACGATAT TCAGTCCCTC ATAATACACA
W. sinensis                TATTTTTCTC CGAGTCCGTA TGGACGATAT TCAGTCCCTC ATAATACACA
Glycyrrhiza lepidota       TATTTTTATC CGAGTCCGTA TGGACGATAT TCAGTCCCTC ATAATACAAA
G.uralensis                TATTTTTATC CGAGTCCGTA TGGACGATAT TCAGTCCCTC ATAATACAAA
G.glabra                   TATTTTTATC CGAGTCCGTA TGGACGATAT TCAGTCCCTC ATAATACAAA
Meristotropis xathioides   TATTTTTATC CGAGTCCGTA TGGACGATAT TCAGTCCCTC ATAATACAAA
Robinia pseudoacacia       TATTTTTACA AAAGTCCTTA TGGACGATAT TCAGGCCATC ATAGTACAAA
Lotus japonicus            TATTTTTATC CGAGTCCTTA TCAACGATAT TCAGTCCCTC ATAATACAAA
Vicia alpestris            CTCTTGTTTA TCAGATCGTT CGGTAGTACT ACAGTCCATC ATAATACCAT
V.sativa                   TATTTTTCTC CGAGTTCGTA TCGAAGATAT TCAGTGCCTC ATACTACAAG
V.peregrina                TATTTTTCTC CGAGTTCGTA TCGAAGATAT TCAGTGCCTC ATACTACAAG
V.sepium                   TATTTTTCTC CGAGTTCGTA TCGAAGATAT TCAGTGCCTC ATACTACAAG
V. faba                    TATTTTTCTC CGAGTTCGTA TGGAAGATAT TCAGTCCCTC ATACTACAAG
```

```
V.canescens               TATTTTTCTC CGAGTTCGTA TGGAAGATAT TCAGTCCCTC ATACTACAAG
V.monantha                TATTTTTCTC CGAGTTCGTA TGGAAGATAT TCAGTCCCTC ATACTACAAG
V.tetrasperma             TATTTTTCTC CGAGTTCGTA TGGAAGATAT TCAGTCCCTC ATACTACAAG
V.narbonensis             TATTTTTCTC CGAGTTCGTA TGGAAGATAT TCAGTCCCTC ATACTACAAG
Lens culinaris            TATTTTTCTC CGAGTTCATA TGGAAGATAT TCAGTCCCTC ATACTACAAG
L.orientalis              TATTTTTCTC CGAGTTCATA TGGAAGATAT TCAGTCCCTC ATAATACAAG
V.ervilia                 TATTTTTCTC CGAGTTCGTA TGGAAAATAT TAAGTCCCTC ATACTTCAAG
Galega officinalis        TATTTTTCTA CGAGTTCGTA TGGAAGATAT TCAGTCCCTC ATACTACAAG
Lathyrus palustris_K      TCGTGTTTGG GAAATTAAAA TTAAAGATAT ATCCTGCTTA ATATTAGTAA
L.palustris_H             TCGTGTTTGG GAAATTAAAA TGCAAGATAT ATGCTGCTTA ATATTAGTAA
L.davidii                 TCGTATTTGG AAAATTCAAA TTAAAGATGT TAATTGTTTA ACATTAGTAA
L.japonicus               TCGTGTTCGG GAAATTGATA TTCAAAATAT TTGCTGCTTA ATATTAGTAA
L.ochroleucus             TCGTGTTTGG GAAATATCAA CTCAAGATAT TAGCTGCTTA ATATTAGTCA
L.littoralis              TCATGTTCGG GAAATTAATA TGAAAAATAT TTGCTGCTTA ATATTAGTAA
L.venosus                 TCGTGTTTGG GAAATTGATA TGAAAGATAT TAGATGCTTA ATATTAGTAA
L.graminifolius           TCGTATTTGG AAGATTGAAA TGAAAGATAT TAGATGCTTA ATATTAGTAA
L.pubescens               TACTGTTCTG GAAGTTCCTC TGGAAGATAG TTTATCGATA ATCCTCAAAA
L.sativus_KJ              TAATGTTCGC CGCATTTTAA TAAAAGAAAT TTCGTGCCTC ATACTAGTAA
L.sativus_HM              TAATGTTCGC CGCATTTTAA TAAAAGAAAT TTCGTGCCTC ATACTAGTAA
L.pseudocicera            TAATGTTCGG CGCATTTTAA TAAAAGAAAT TTCGTGCCTT ATACTAGTAA
L.chloranthus             ---------- ---------- ---------- ---------- ----------
L.pratensis               ---------- ---------- ---------- ---------- ----------
L.odoratus_HM             ---------- ---------- ---------- ---------- ----------
L.odoratus_kJ             ---------- ---------- ---------- ---------- ----------
L.hirsutus                ---------- ---------- ---------- ---------- ----------
L.annuus                  ---------- ---------- ---------- ---------- ----------
L.cirrhosus               TACTGTTCTT CACATTCCGC TAAAAGATAT TACGTGCCTC ATACTAAGGA
L.latifolius              TACTGTTCTT CACATTCGGC TAAAAGATAT TACGTGCCTC ATACTCAAGA
L.tingitanus              TACTGTTTTA CAAGTTCCTA TGAAAGATAT TCCGTGCCTC ATACTAAAAA
Pisum sativum_H           TATTATTCCC AGAGTTCCTA TGAACGCTAG TCAGTGCCTC ATACAAAAAA
Pisum sativum_K           TATTATTCCC AGAGTTCCTA TGAACGCTAG TCAGTGCCTC ATACAAAAAA
Pisum sativum_HG          TATTATTCCC AGAGTTCCTA TGAACGCTAG TCAGTGCCTC ATACAAAAAA
Pisum fulvum              TATTATTCCC AGAGTTCCTA TGAACGCTAG TCAGTGCCTC ATACAAAAAA
Vavilovia formosa         TATTATTCCC AGAGTTCATA TGAACAATAG TCAGTGCCTC ATACAAAAAA
L.clymenum                ---------- ---------- ---------- ---------- ----------
L.ochrus                  TTTGTTCGAT AAAAAACATC TAGTGGTATA TATTACTCGT TACGGATTTT
```

```
                             851
Trifolium strictum          CTAACCCTAA CCC------- ----AGAACC ---------- ---------T
T. glanduliferum            CTAACCCTAA CCC------- ----AGAACC ---------- ---------T
T. boissieri                CTAACCCTAA CCC------- ----AGAACC ---------- ---------T
T. aureum                   CTAACCCTAA CCC------- ----AGAACC ---------- ---------T
T.meduseum                  CTAACCCTAA TCC------- ----AGAACC ---------- ---------T
T.subterraneum              CTAACCCTAA TCC------- ----AGAACC ---------- ---------T
T.pratense                  CTAACCCTAA TCC------- ----AGAACC ---------- ---------T
T.hybridum                  CTAACCCTAA CCC------- ----AGAACC ---------- ---------T
T.semipilosum               CTAACCCTAA CCC------- ----AGAACC ---------- ---------T
T.occidentale               CTAACCCTAA CCC------- ----AGAACC ---------- ---------T
T.repens                    CTAACCCTAA CCC------- ----AGAACC ---------- ---------T
T.lupinaster                CTAACCCTAA CCC------- ----AGAACC ---------- ---------T
Cicer oxyodon               CTAACCCTAA CCC------- ----AGAACC ---------- ---------T
C. chorrassanicum           CTAACCCTAA CCC------- ----AGAACC ---------- ---------T
C. arietinum                CTAACCCTAA CCC------- ----AGAACC ---------- ---------T
Medicago truncatula_KF      CTAACCCTAA CCC------- ----AGAACC ---------- ---------C
M.truncatula_AC             CTAACCCTAA CCC------- ----AGAACC ---------- ---------C
M.sativa_K                  CTAACCCTAA CCC------- ----AGAACC ---------- ---------C
M.sativa                    CTAACCCTAA CCC------- ----AGAACC ---------- ---------C
M.papillosa                 CTAACCCTAA CCC------- ----AGAACC ---------- ---------C
M.hybrida                   CTAACCCTAA CCC------- ----AGAACC ---------- ---------C
M.falcata                   CTAACCCTAA CCC------- ----AGAACC ---------- ---------C
M.sativa_KU                 CTAACCCTAA CCC------- ----AGAACC ---------- ---------C
Melilotus albus             CTAACCCTAA CCC------- ----AGAACC ---------- ---------T
Hedysarum formosum          CTAACCCAG- ---------- ------AACC ---------- ---------T
H.varium                    CTAACCCAG- ---------- ------AACC ---------- ---------T
H.singarense                CTAACCCAG- ---------- ------AACC ---------- ---------T
H.minjanense                CTAACCCAG- ---------- ------AACC ---------- ---------T
Tavrniera glabra            CTAATCCAG- ---------- ------AACC ---------- ---------T
T.diffusa                   CTAACCCAG- ---------- ------AACC ---------- ---------T
Greuteria membranacea       CTAACCCAG- ---------- ------AACC ---------- ---------T
Corethrodendron scoparium   CTAACCCAG- ---------- ------AACC ---------- ---------T
Eversmannia subspinosa      CTAACCCAG- ---------- ------AACC ---------- ---------T
Onobrychis bungei           CTAACCCCG- ---------- ------AACC ---------- ---------T
O.cornuta                   CTAACCCAG- ---------- ------AACC ---------- ---------T
O.michauxii                 CTAACCCAG- ---------- ------AACC ---------- ---------T
O.subacaulis                CTAACCCAG- ---------- ------AACC ---------- ---------T
O.teheranica                CTAACCCAG- ---------- ------AACC ---------- ---------T
Alhagi maurorum             CTAATTCAGC AG-------- ------AACC ---------- ---------T
Caragana microphylla        CTAACCCAG- ---------- ------AACC ---------- ---------T
C.korshinski                CTAACCCAG- ---------- ------AACC ---------- ---------T
C.kozlowii                  CTAACCCAG- ---------- ------AACC ---------- ---------T
C.rosea                     CTAACCCAG- ---------- ------AACC ---------- ---------T
Tibetia liangshanensis      CTAACCCAG- ---------- ------AACC ---------- ---------T
Gueldenstaedtia verna       CTAGGCCAA- ---------- ------AACC ---------- ---------T
Halimodendron halodendron   AAGCGCCGGC AG-------- ------ATCC ---------- ---------T
Smirnowia turkestana        ATAACCCGG- ---------- ------AACC ---------- ---------T
Eremosparton flaccidum      ATAACCCGG- ---------- ------AACC ---------- ---------T
Colutea persica             ATAACCCAG- ---------- ------AACC ---------- ---------T
C. triphylla                ATAACCCAG- ---------- ------AACC ---------- ---------T
Sphaerophysa salsula        ATAACCCAG- ---------- ------AACC ---------- ---------T
Podlechiella vogelii        ATAACCCAG- ---------- ------AACC ---------- ---------T
Carmichaelia australis      ATAACCCAG- ---------- ------AACC ---------- ---------T
Sutherlandia frutescens     ATAACCCCG- ---------- ------AACC ---------- ---------T
Astragalus membranaceous    ATAACCCAG- ---------- ------AACC ---------- ---------T
A.mongholicus               ATAACCCAG- ---------- ------AACC ---------- ---------T
A.nakaianus                 ATAACCCAG- ---------- ------AACC ---------- ---------T
A.macropelmatus             ATAACCCAG- ---------- ------AACC ---------- ---------T
A.iranicus                  ATAACCCAG- ---------- ------AACC ---------- ---------T
A.denudatus                 ATAACCCAG- ---------- ------AACC ---------- ---------T
A.odoratus                  ATAACCCAG- ---------- ------AACC ---------- ---------T
Oxytropis szovitsii         ATAACCCAG- ---------- ------AACC ---------- ---------T
O.iranica                   ATAACCCAG- ---------- ------AACC ---------- ---------T
O.kotschyana                ATAACCCAG- ---------- ------AACC ---------- ---------T
Erophaca baetica            ATAAACCCG- ---------- ------AACC ---------- ---------T
Wisteria floribunda         GTCAAATCGA AACTAAAAAA GATAGTAGGT CTAAAAAAGA AAGTCAGTAT
W. sinensis                 GTCAAATCGA AACTAAAAAA GATAGTAGGT CTAAAAAAGA AAGTCAGTAT
Glycyrrhiza lepidota        GTCAAATAGA AACTAAAAAA GATAGTAGGT ---------- --------AT
G.uralensis                 GTCAAATAGA AACTAAAAAA GATAGTAGGT ---------- --------AT
G.glabra                    GTCAAATAGA AACTAAAAAA GATAGTAGGT ---------- --------AT
Meristotropis xathioides    GTCAAATAGA AACTAAAAAA GATAGTAGGT ---------- --------AT
Robinia pseudoacacia        GTAAAGTAGG AAATTCAAAA GATGGTAAGT AT-------- -------CTT
Lotus japonicus             GTAAAAAGGA AAATAAAAAA GAGGGTAGGT AT-------- -------CTT
Vicia alpestris             CCTCCTCAG- ---------- ------AACC ---------- ---------T
V.sativa                    CTAACCCAG- ---------- ------AACC ---------- ---------T
V.peregrina                 CTAACCCAG- ---------- ------AACC ---------- ---------T
V.sepium                    CTAACCCAG- ---------- ------AACC ---------- ---------T
V. faba                     CTAACCCAG- ---------- ------AACC ---------- ---------T
```

```
V.canescens               CTAACCCAG- ---------- ------AACC ---------- ---------T
V.monantha                CTAACCCAG- ---------- ------AACC ---------- ---------T
V.tetrasperma             CTAACCCAG- ---------- ------AACC ---------- ---------T
V.narbonensis             CTAACCCAG- ---------- ------AACC ---------- ---------T
Lens culinaris            CTAACCCAG- ---------- ------AACC ---------- ---------T
L.orientalis              GTAACCCAG- ---------- ------AACC ---------- ---------T
V.ervilia                 CTAACCCAG- ---------- ------AACC ---------- ---------T
Galega officinalis        CTAACGCTAA CCC------- ----AGAACC ---------- ---------T
Lathyrus palustris_K      CTAATAATAT ---------- ---------- ---------- ---------T
L.palustris_H             CTAATAATTT ---------- ---------- ---------- ---------T
L.davidii                 CTAACCCTCT ---------- ---------- ---------- ---------T
L.japonicus               CTAACTACTA ---------- ---------- ---------- ---------T
L.ochroleucus             CTAATACCTA ---------- ---------- ---------- ---------C
L.littoralis              CTAACAATTA ---------- ---------- ---------- ---------T
L.venosus                 CTAACCCTTA ---------- ---------- ---------- ---------C
L.graminifolius           CTAATATCTA ---------- ---------- ---------- ---------T
L.pubescens               TGAACCAAAA ---------- ---------- ---------- ---------T
L.sativus_KJ              CTAACCGAAT ---------- ---------- ---------- ---------C
L.sativus_HM              CTAACCGAAT ---------- ---------- ---------- ---------C
L.pseudocicera            CTCACCGAAT ---------- ---------- ---------- ---------C
L.chloranthus             ---------- ---------- ---------- ---------- ----------
L.pratensis               ---------- ---------- ---------- ---------- ----------
L.odoratus_HM             ---------- ---------- ---------- ---------- ----------
L.odoratus_kJ             ---------- ---------- ---------- ---------- ----------
L.hirsutus                ---------- ---------- ---------- ---------- ----------
L.annuus                  ---------- ---------- ---------- ---------- ----------
L.cirrhosus               GTGATCCAAT ---------- ---------- ---------- ---------A
L.latifolius              CTGACCCAAT ---------- ---------- ---------- ---------A
L.tingitanus              CGAACCCAGA ---------- ---------- ---------- ---------T
Pisum sativum_H           CTAACCCAGA TAGT------ ---AATGATG TCCTTGTCCT TTCTATGAAA
Pisum sativum_K           CTAACCCAGA TAGT------ ---AATGATG TCCTTGTCCT TTCTATGAAA
Pisum sativum_HG          CTAACCCAGA TAGT------ ---AATGATG TCCTTGTCCT TTCTATGAAA
Pisum fulvum              CTAACCCAGA TAGT------ ---AATGATG TCCTTGTCCT TTCTATGAAA
Vavilovia formosa         TGAACCGAGA TAGT------ ---AATGATG TCCTT----- -TCTATGAAA
L.clymenum                ---------- ---------- ---------- ---------- ----------
L.ochrus                  TTGGAAAAGG TCGA------ ---TGTATTG TTTCCCAAGT TCCTATGAAA
```

```
                             901
Trifolium strictum          AGTAGTGGTG TCCTTTATAT GCAAACCAGA GAACAGGGGA CCATTCCCTT
T. glanduliferum            AGTAGTGGTG TCCTTTATAT GCAAACCAGA GAACAGGGGA CCATTCCCTT
T. boissieri                AGTAGTGGTG TCCTTTATAT GCAAACCAGA GAACAGGGGA CCATTCCCTT
T. aureum                   AGTAGTGGTG TCCTTTATAT GCAAACCAGA GAACAGGGGA CCATTCCCTT
T.meduseum                  AGTAGTGGTG TCCTTTATAT GCAAACCAGA GAACAGGGGA CCATTCCCTT
T.subterraneum              AGTAGTGGTG TCCTTTATAT GCAAACCAGA GAACAGGGGA CCATTCCCTT
T.pratense                  AGTAGTGGTG TCCTTTATAT ACAAACCAGA GAACAGGGGA CCATTCCCTT
T.hybridum                  AGTAGTGGTG TCCTTTATAT GCAAACCAGA GAACAGGGGA CCATTCCCTT
T.semipilosum               AGTAGTGGTG TCCTTTATAT GCAAACCAGA GAACAGGGGA CCATTCCCTT
T.occidentale               AGTAGTGGTG TCCTTTATAT GCAAACCAGA GAACAGGGGA CCATTCCCTT
T.repens                    AGTAGTGGTG TCCTTTATAT GCAAACCAGA GAACAGGGGA CCATTCCCTT
T.lupinaster                AGTAGTGGTG TCCTTTATAT GCAAACCAGA GAACAGGGGA CCATTCCCTT
Cicer oxyodon               AGTAGCGGTG TCCTTTATAT GCAAACCAGA GAACAAGGGA CCATTCCCTT
C. chorrassanicum           AGTAGCGGTG TCCTTTATAT GCAAACCAGA GAACAAGGGA CCATTCCCTT
C. arietinum                AGTAGCGGTG TCCTTTATAT GCAAACCAGA GAACAAGGGA CCATTCCCTT
Medicago truncatula_KF      AGTAGTGGTG TCCTTTATAT GCAAACACGA GAACAAGGGA CCATTCCTTT
M.truncatula_AC             AGTAGTGGTG TCCTTTATAT GCAAACACGA GAACAAGGGA CCATTCCTTT
M.sativa_K                  AGTAGTGGTG TCCTTTATAT GCAAACAAGA GAACAAGGGA CCATTCCTTT
M.sativa                    AGTAGTGGTG TCCTTTATAT GCAAACAAGA GAACAAGGGA CCATTCCTTT
M.papillosa                 AGTAGTGGTG TCCTTTATAT GCAAACAAGA GAACAAGGGA CCATTCCTTT
M.hybrida                   AGTAGTGGTG TCCTTTATAT GCAAACAAGA GAACAAGGGA CCATTCCTTT
M.falcata                   AGTAGTGGTG TCCTTTATAT GCAAACAAGA GAACAAGGGA CCATTCCTTT
M.sativa_KU                 AGTAGTGGTG TCCTTTATAT GCAAACAAGA GAACAAGGGA CCATTCCTTT
Melilotus albus             AGTAGTGGTG TCCTTTATAT GCAAACCAGA GAACAAGGGA CCATTCCCTT
Hedysarum formosum          CGTACGGGTG TCCTTTATAT GTACACCAGA GAACAGGGGG CCATCCCCTT
H.varium                    CGTACGGGTG TCCTTTATAT GTACACCAGA GAACAGGGGG CCATTCCCTT
H.singarense                CGTATGGGTG TCCTGTATAT GTACACCAGA GAACAGGGGG CCATTCCCTT
H.minjanense                CGTACGGGTG TCCTTTATAT GTACACCAGA GAACAGGGGG CCATTCCCTT
Tavrniera glabra            CGTACGGGTG TCCTTTATAT GTACACCAGA GAACAGGGGG CCATTCCCTT
T.diffusa                   CGTACGGGTG TCCTTTATAT GTACACCAGA GAACAGGGGG CCATTCCCTT
Greuteria membranacea       CGTACGGGTG TCCTTTATAT GTACACCAGA GAACAGGGGG CCATTCCCTT
Corethrodendron scoparium   CGTACGGGTG TCCTTTATAT GTACACCAGA GAACAGGGGG CCATTCCCTT
Eversmannia subspinosa      CGTACGGGTG TCCTTTATAT GTACACCAGA GAACAGGGGG CCATTCCCTT
Onobrychis bungei           CGTAAGGGTG TTCTTTATAT GTACACCAGA GAACAGGGGG CCATTCCCTT
O.cornuta                   CGTAAGAGTG TCCTTTATAT GTACACCAGA GAACAGGGGG CCATTCCCTT
O.michauxii                 CGTACGGGTG TTCTTTATAT GTACACTAGA GAACAGGGGG CCATTCCCTT
O.subacaulis                CGTACGGGTG TTCTTTATAT GTACACTAGA GAACAGGGGG CCATTCCCTT
O.teheranica                CGTACGGGTG TTCTTTATAT GTACACTAGA GAACAGGGGG CCATTCCCTT
Alhagi maurorum             CATGCGGGTG TCCTTTATAT GTACACCAGA GAACAGGGGG CCATTCCCTT
Caragana microphylla        CGTACTGGTG TCCTTTATAT GTACACCAGA GAACAGGGGG CCATTCCCTT
C.korshinski                CGTACTGGTG TCCTTTATAT GTACACCAGA GAACAGGGGG CCATTCCCTT
C.kozlowii                  CGTACTGGTG TCCTTTATAT GTACACCAGA GAACAGGGGG CCATTCCCTT
C.rosea                     CGTACTGGTG TCCTTTATAT GTATACCAGA GAACAGGGGG CCATTCCATT
Tibetia liangshanensis      CGTACTGGTG TCCTTTATAT GTATACCAGA GAACAGGGGG CCATTCCCTT
Gueldenstaedtia verna       CGAACGGGGG CCCTTTATGT GTACACCAGA AAACAGGGGG CCGTTCCCTT
Halimodendron halodendron   TGTAGTGTCG TCCATGAGAC CTCCACCAGA CAGCGGGGCG CCACTCCCAT
Smirnowia turkestana        TTTAGTGGTG TCCTTTATAT GCACACCAGA GAACAGGGGG ACATTCCCTT
Eremosparton flaccidum      CGTAGTGGTG TCCTTTATAT GCACACCAGA GAACAGGGGG ACATTCCCTT
Colutea persica             TTTAGTGGTG TCCTTTATAT GCACACCAGA GAACAGGGGG ACATTCCCTT
C. triphylla                TTTAGTGGGG TCCTTTATAT GCACACCAGA GAACAGGGGG ACATTCCCTT
Sphaerophysa salsula        TGTAGTGGGG TCCTTTATAT GCACACCAGA GAACAAGGGG ACATTCCCTT
Podlechiella vogelii        TTTAGTGGTG TCCTTTATAT GCACACCAGA GAACAGGGGG ACATTCCCCT
Carmichaelia australis      CTTAGTGGTG TCCTTTATAT GCACACCAGA GAACAGGGGG ACATTCCCTT
Sutherlandia frutescens     CGTAGTGGTG TCCTTTATAT GCACACCAGA GAACAGGGGG ACATTCCCTT
Astragalus membranaceous    CTTAGTGGTG TCCTTTATAT GCACACCAGA GAACAGGGGG ACATTCCCTT
A.mongholicus               CTTAGTGGTG TCCTTTATAT GCACACCAGA GAACAGGGGG ACATTCCCTT
A.nakaianus                 TTTAGTGGTG TCCTTTATAT GCACACCAGA GAACAGGGGG ACATTCCCTT
A.macropelmatus             CTTAGTGGTG TCCTTTATAT GCACACCAGA GAACAGGGGG ACATTCCCTT
A.iranicus                  CTTAGTGGTG TCCTTTATAT GCACACCAGA GAACAGGGGG ACATTCCCTT
A.denudatus                 CTTAGTGGTG TCCTTTATAT GCACACCAGA GAACAGGGGG ACATTCCCTT
A.odoratus                  CTTAGTGGTG TCCTTTATAT GCACACCAGA GAACAGGGGG ACATTCCCTT
Oxytropis szovitsii         CGTAGTGGCG TCCTTTATAT GCACACCAGA GAACAGGGGG ACATTCCCTT
O.iranica                   CGTAGTGGTG TCCTTTATAT GCACACCAGA GAACAGGGGG ACATTCCCTT
O.kotschyana                CGTAGTGGTG TCCTTTATAT GCACACCAGA GAACAGGGGG ACATTCCCTT
Erophaca baetica            TTTAGTGGTA TCCCTTATAT GCACACCCGA GAACAGGGGG ACATTCCCTT
Wisteria floribunda         CGTAGTGGTG TCCTTTATAT GCAAACCAGA GAACAGGGGG CCATCCCCCT
W. sinensis                 CGTAGTGGTG TCCTTTATAT GCAAACCAGA GAACAGGGGG CCATCCCCCT
Glycyrrhiza lepidota        CGTAATGGTG TCCTTTATAT GCAAACCAGA GAACAGGGAG CCATTCCCTT
G.uralensis                 CGTAATGGTG TCCTTTATAT GCAAACCAGA GAACAGGGAG CCATTCCCTT
G.glabra                    CGTAATGGTG TCCTTTATAT GCAAACCAGA GAACAGGGAG CCATTCCCTT
Meristotropis xathioides    CGTAATGGTG TCCTTTATAT GCAAACCAGA GAACAGGGGG CCATTCCCTT
Robinia pseudoacacia        CGTGGTGGTG TCCTTTATAT TCAAACTCGA GAACAGGGGG CCTTTGCCTT
Lotus japonicus             CGCAGTGGTG TCCTTTATAT GCAAACCAGA GAACAGGGGG CCATTCCCCT
Vicia alpestris             CATCACTGTG TCCTTTATAA GCAAACCACA GAACAAGGGA CCATTCCCTT
V.sativa                    AGTAGTGGTG TCCTTTATAT GCAAACCAGA GAACAGGGGA CCATTCCCTT
V.peregrina                 AGTAGTGGTG TCCTTTATAT GCAAACCAGA GAACAGGGGA CCATTCCCTT
V.sepium                    AGTAGTGGTG TCCTTTATAT GCAAACCAGA GAACAGGGGA CCATTCCCTT
V. faba                     AGTAGTGGTG TCCTTTATAT GCAAACCAGA GAACAGGGGA CCATTCCCTT
```

```
V.canescens              AGTAGTGGTG TCCTTTATAT GCAAACCAGA GAACAGGGGA CCATTCCCTT
V.monantha               AGTAGTGGTG TCCTTTATAT GCAAACCAGA GAACAGGGGA CCATTCCCTT
V.tetrasperma            AGTAGTGGTG TCCTTTATAT GCAAACCAGA GAACAGGGGA CCATTCCCTT
V.narbonensis            AGTAGTGGTG TCCTTTATAT GCAAACCATA GAACAGGGGA CCATTCCCTT
Lens culinaris           AGTAGTGGTG TTCTTTATAT GCAAACCAGA GAACAGGGGA CCATTCCCTT
L.orientalis             TGTAGTGGTG TTCTTTATAT GCAAACCAGA GAACCGGGGA CCATTCCCCT
V.ervilia                AGTAGTGGTG TCCTTTATAT GCAAACCAGA GAACAGGGGA CCATTCCCTT
Galega officinalis       AGTAGTGGTG TCCTTTATAT GCAAACCAGA GAACAGGGGA CCATTCCCCT
Lathyrus palustris_K     TGGGGTGATG TGTTATATTT AAAAACCCGC GAACAGGAGA CCTTGGCCTT
L.palustris_H            TGTGGTGATG TCTTGTATTT AAAAACCCGC GAACAGGAGA CCTTCGCCTT
L.davidii                TGGGGTGATG TTTTATACTT AAAATTAAAT ACCGGCGAAC AGGTGGCCTT
L.japonicus              TGGGGTGATG TGCTGTATTT ACAAACTGTC GAACAGGAAA CCTTGAACTT
L.ochroleucus            TGGGGGGATG TTTTGTATTT ACAAACCTTC GAACAGAAGA CCATTACATT
L.littoralis             TGGGGTGATG TGCTGTATTT ACAAACCATC GAACAGGAAA CCTTGAACTT
L.venosus                TGGGGGGATG TGATGTATTT ACAAACCATC AAACAGGGGA CCTTTACCTT
L.graminifolius          TGGGGGGATG TGCTGCATTT AAAAACGAGA GATCACAAGA CTTTCCCCTT
L.pubescens              TGGGGTGATG TGCTTTATCT GGAAACCAGA GAACTGGGGA CCTTGACCTT
L.sativus_KJ             TGGGGTGATG TCCTTTATAT TGAAACCAGA AAACACGGGA CCTTGCCGAT
L.sativus_HM             TGGGGTGATG TCCTTTATAT TGAAACCAGA AAACACGGGA CCTTGCCGAT
L.pseudocicera           TGGGGTGATG TCCTTTATAT TGAAACCAGA AAACACGGGA CCTTGCCGAT
L.chloranthus            ---------- ---------- ---------- ---------- ----------
L.pratensis              ---------- ---------- ---------- ---------- ----------
L.odoratus_HM            ---------- ---------- ---------- ---------- ----------
L.odoratus_kJ            ---------- ---------- ---------- ---------- ----------
L.hirsutus               ---------- ---------- ---------- ---------- ----------
L.annuus                 ---------- ---------- ---------- ---------- ----------
L.cirrhosus              TTTGGTGATG TCCTTTATAT TCAAACCAGA GAACAGGGGA TCTTGGCGGT
L.latifolius             TTTGGTGATG TCCTTTATAT TCAAACCAGA GAACAGGGGA TCTTGGCGGT
L.tingitanus             TGTGGTGATG TCCTTTATAT GAAAACCAGA GAACAGGGGA CCGTGCCCTT
Pisum sativum_H          ACCAGAGAAA ---------- --------GA GAAAAGGGGA CCATTCCCTT
Pisum sativum_K          ACCAGAGAAA ---------- --------GA GAAAAGGGGA CCATTCCCTT
Pisum sativum_HG         ACCAGAGAAA ---------- --------GA GAAAAGGGGA CCATTCCCTT
Pisum fulvum             ACCAGAGAAA ---------- --------GA GAAAAGGGGA CCATTCCCTT
Vavilovia formosa        ACCAGAGAAA AGGGGACCAT GAAAACCAGA GAAAAGGGGA CCATTCCCTT
L.clymenum               ---------- ---------- ---------- ---------- ----------
L.ochrus                 GAGATTAAGT GCCTCATACT AAAAACTGAC CCAGATCGTC ATGATGTCCT
```

```
                             951
Trifolium strictum          GACTCCTGTT GATGATTATT ATGATAGGAC TCCACGC--- AACGTTATAC
T. glanduliferum            GACTCCTGTT GATGATTATT ATGATAGGAC TCCACGC--- AACGTTATAC
T. boissieri                GACTCCTGTT GATGATTATT ATGATAGGAC TCCGCGC--- AACGTTATAC
T. aureum                   GACTCCTGTT GATGATTATT ATGATAGGAC TCCACGC--- AACGTTATAC
T.meduseum                  GACTCCTGTT GATAATTATT ATGATAGGAC TCCACAC--- AACGTTATAC
T.subterraneum              GACTCCTGTT GATAATTATT ATGATAGGAC TCCACAC--- AACGTTATAC
T.pratense                  GACTCCTGTT GATAATTATT ATGATAGGAC TCCACGC--- AACGTTATAC
T.hybridum                  GACTCCTGTT GATGATTATT ATGATAGGAC TCCGCGC--- AACGTTATAC
T.semipilosum               GACTCCTGTT GATGATTATT ATGATAGGAC TCCGCGC--- AACGTTATAC
T.occidentale               GACTCCTGTT GATGATTATT ATGATAGGAC TCCGCGC--- AACGTTATAC
T.repens                    GACTCCTGTT GATGATTATT ATGATAGGAC TCCGCGC--- AACGTTATAC
T.lupinaster                GACTCCTGTT GATGATTATT ATGATAGGAC TCCACGC--- AATGTTATAC
Cicer oxyodon               GACTCCTGTT GATGATTATT ATGATAGGAC TCCACGC--- AACGTTATAC
C. chorrassanicum           GACTGCTGTT GATGATTATT ATGATAGGAC TCCACGC--- AACGTTATAC
C. arietinum                GACTCCTGGT GATGATTATT ATGATAGGAC TCCACGC--- AACGTTATAC
Medicago truncatula_KF      GACTCCTGTT GATGATTATT ATGATAGGAC TCCACGC--- AACGTTATAC
M.truncatula_AC             GACTCCTGTT GATGATTATT ATGATAGGAC TCCACGC--- AACGTTATAC
M.sativa_K                  GACTCCTGTT GATGATTATT ATGATAGGAC TCCACGC--- AACGTTATAC
M.sativa                    GACTCCTGTT GATGATTATT ATGATAGGAC TCCACGC--- AACGTTATAC
M.papillosa                 GACTCCTGTT GATGATTATT ATGATAGGAC TCCACGC--- AACGTTATAC
M.hybrida                   GACTCCTGTT GATGATTATT ATGATAGGAC TCCACGC--- AACGTTATAC
M.falcata                   GACTCCTGTT GATGATTATT ATGATAGGAC TCCACGC--- AACGTTATAC
M.sativa_KU                 GACTCCTGTT GATGATTATT ATGATAGGAC TCCACGC--- AACGTTATAC
Melilotus albus             GACTCCTGTT GATGATTATT ATGATAGGAC TCCCCGC--- AACGTTATAC
Hedysarum formosum          GACTCCTGCT GATGATTATT ATGAGAGGAC TCCACGCGCC AGCGTTTTCG
H.varium                    GACTCCTGCT GATGATTATT ATGCGAGGAC TCCACGCGCC AGCGTTTTCG
H.singarense                GACTCCTGCT GATGATTA-- -TGAGAGGAC TCCACGCGCC AGCGTTTTCG
H.minjanense                GACTCCTGTT GATGATTATT ATGAGAGGAC TCCACGCGCC AGCGTTTTCG
Tavrniera glabra            GACTCCTGTT GATGATTATT ATGACAGGAC TCCACGCGCC AGCGTTTTTG
T.diffusa                   GACTCCTGTT GATGATTATT ATGACAGCAC TCCACGCGCC AGCGTTTTTG
Greuteria membranacea       GACTCCTCTT GATGATTATT ATGAGAGGAC TCCACGCACC AGCGTTTTCG
Corethrodendron scoparium   GACTCCTCTT GATGATTATT ATGAGAGGAC TCCACGCACC AGCGTTTTCG
Eversmannia subspinosa      GACTCCTCTT GATGATTATT ATGAGAGGGC TCCACGCACC AGCGTTTTCG
Onobrychis bungei           GACCCCTCTT GATGATTATT ATGAGAGGAC TCCACGCACC AGCGTTTTTG
O.cornuta                   GACTCCTCTT GATGATTATT ATGAGAGGAC TCCACGCACC AGCGTTTTCG
O.michauxii                 GACTCCTCTT GATGATTATT ATGAGATGAC TCCACGCACC AGCGTTTTTG
O.subacaulis                GACTCCTCTT GATGATTATT ATGAGATGAC TCCACGCACC AGCGTTTTTG
O.teheranica                GACTCCTCTT GATGATTATT ATGAGATGAC TCCACGCACC AGCGTTTTTG
Alhagi maurorum             GACTCCTGTT GATTATTATT ATGATAAGAC TCCACGCGCC AGCGTTTTCC
Caragana microphylla        GACTCCTGTT GATGTTTATT ATGATAGGAC TCCGCGCGCC AGCGTTTTCG
C.korshinski                GACTCCTGTT GATGTTTATT ATGATAGGAC TCCGCGCGCC AGCGTTTTCG
C.kozlowii                  GACTCCTGTT GATGTTTATT ATGATAGGAC TCCGCGCGCC AGCGTTTTCG
C.rosea                     GACTCCTGTT GATGTTTATT ATGATAGGAC TCCGCGCGCC AGCGTTTTCG
Tibetia liangshanensis      GACTCCTCTT GATGATTATT ATGATAGGAC TCCACGCGCC AGCGTTTTCC
Gueldenstaedtia verna       GACCCC---- ---------- ---------- ---------- ----------
Halimodendron halodendron   TACTCCTGTT GATGTTTATT ATAATAGGAC TCCGCGCTCC AGCGTTTTAC
Smirnowia turkestana        GACTCTTGTT GATGATTATT ATGATAGGAC TCCCCGC--- AACATTTTAC
Eremosparton flaccidum      GACTCTTGTT GATGATTATT ATGATAGGAC TCCACGC--- AACATTTTAC
Colutea persica             GACTCTTGTT GATGATTATT ATGATAGGAC TCCCCGC--- AACATTTTAC
C. triphylla                GACTCTTGGT GATGATTATT ATGATAGGAC TCCCCGC--- AACATTTTAC
Sphaerophysa salsula        GACTCTTGTT GAAGATTATT TTGATAGGAC TCCCCGC--- AACATTTTAC
Podlechiella vogelii        GACTCTTGTT GATGATTATT ATGATAGGAC TCCGCGC--- AACATTTTAC
Carmichaelia australis      GACTCTTGTT GATGATTATT ATGATAGGAC TCCGCGC--- AACATTTTAC
Sutherlandia frutescens     GACTCTTGTT GATGATTATT ATGATAGGGC TCCGCGC--- AACATTTTAC
Astragalus membranaceous    GACTCTTGTT GATGATTATT ATGATAGGAC TCCACGG--- AACATTTTAC
A.mongholicus               GACTCTTGTT GATGATTATT ATGATAGGAC TCCACGG--- AACATTTTAC
A.nakaianus                 GACTCTTGTT GATGATTATT ATGATAGGAC TCCCCGG--- AACATTTTAC
A.macropelmatus             GACCCTTGTT GATGATTATT ATGATAGGAC TCCACGG--- AACATTTTAC
A.iranicus                  GACTCTTGTT GATGATTATT ATGATAGGAC TCCACGG--- AACATTTTAC
A.denudatus                 GACTCTTGTT GATGATTATT ATGATAGGAC TCCACGG--- AACATTTTAC
A.odoratus                  GACTCTTGTT GATGATTTTT ATGATAGGAC TCCACGG--- AACATTTTAC
Oxytropis szovitsii         GACTCTTGTT GATGATTATT ATGATAGGAC TCCGCGC--- AACATTTTAC
O.iranica                   GACTCTTGTT GATGATTATT ATGATAGGAC TCCGCGC--- AACATTTTAC
O.kotschyana                GACTCTTGTT GATGATTATT ATGATAGGAC TCCGCGC--- AACATTTTAC
Erophaca baetica            GACTCTTGTT GATGATTATT ATGATAGGAC TCCCCGC--- AACATTTTGC
Wisteria floribunda         GACTCCTATT GATGATTATT ---ATAGGAC TTCAGCC--- AAAGTTATAC
W. sinensis                 GACTCCTATT GATGATTATT ---ATAGGAC TTCAGCC--- AAAGTTATAC
Glycyrrhiza lepidota        GACTCCTATT GATGATTATT ---ATAGGAC TCCACGC--- AAAGTTGCAC
G.uralensis                 GACTCCTATT GATGATTATT ---ATAGGAC TCCACGC--- AAAGTTGCAC
G.glabra                    GACTCCTATT GATGATTATT ---ATAGGAC TCCACGC--- AAAGTTGCAC
Meristotropis xathioides    GACTCCTATT GATGATTATT ---ATAGGAC TCCACGC--- AAAGTTGCAC
Robinia pseudoacacia        GACTCCTATT GACGATTATT ------GGGA TCCATTC--- AAAGTTGCAC
Lotus japonicus             AACTGCTGTT GACGATTCTT ------GGAA TCCACCA--- AAAATTGCAC
Vicia alpestris             GACTCCTCTC GATGAT---- -----AGGTC TTCTCGC--- AACGTTATAC
V.sativa                    GACTCCTGTT GATGAT---- -----AGGAC TCCCCGC--- AACGTTATAC
V.peregrina                 GACTCCTGTT GATGAT---- -----AGGAC TCCCCGC--- AACGTTATAC
V.sepium                    GACTCCTGTT GATGAT---- -----AGGAC TCCCCGC--- AACGTTATAC
V. faba                     GACTCCTGTT GATGAT---- -----AGGAC TCCATGC--- AACGTTATAC
```

```
V.canescens              GACTCCTGTT GATGAT---- -----AGGAC TCCACGC--- AACGTTATAC
V.monantha               GACTCCTGTT GATGAT---- -----AGGAC TCCACGC--- AACGTTATAC
V.tetrasperma            GACTCCTGTT GATGAT---- -----AGGAC TCCACGC--- AACGTTATAC
V.narbonensis            GACTCCTGTT GATGAT---- -----AGGAC TCCACGC--- AACGTTATAC
Lens culinaris           GACTCCTGTT GATGAT---- -----AGGAC TCTACGC--- AACGTTATAC
L.orientalis             GGCTCCCGTT GATGAT---- -----AGGGC TCTACGC--- AACGTTATAC
V.ervilia                GAGTCCTGTT GATGAT---- -----AGGAC TCCACGC--- AACGTTATAC
Galega officinalis       GACTCCTGTT GATGAGTATT ATGATAGGAC TCCGCGT--- AACGTTATCC
Lathyrus palustris_K     AAC---GCCT ATTAAT---- --GGGACTTG T---TAC--- AATCTTAGAG
L.palustris_H            AAC---TCCT ATTAAT---- --GGGAGTTG T---TAC--- AATCTTAGAG
L.davidii                AAC---ACCT AGTAAT---- --GGTAGCTG T---TAC--- ACTCTTAGAG
L.japonicus              AAC---GCCT ATTAAT---- --GGAAGTTG T---TAC--- AATCTTAGAG
L.ochroleucus            AAC---GCCT ATTAAT---- --GGGAGTTG T---TAC--- AATCTTAGAG
L.littoralis             AAC---GCCT ATTAAT---- --GGGAGTTG T---TAC--- AATCTTAGAG
L.venosus                AAC---ACCT ATTAAT---- --GGGACTTG T---TAC--- AATCTTAGAG
L.graminifolius          AAC---GCCT ATTAAT---- --GGGAGTTG T---TAC--- AATCTTAGAG
L.pubescens              AAC---GCCT CCTCAT---- --GCTAGTTG T---TAC--- AATCTTCTAG
L.sativus_KJ             GAC---TCCT ACTAAA---- --GATGGTCG TAGATAT--- ATGCTTGCAA
L.sativus_HM             GAC---TCCT ACTAAA---- --GATGGTCG TAGATAT--- ATGCTTGCAA
L.pseudocicera           GAC---TCCT ACTAAA---- --GATGGTCG TAGATAC--- AGGCTTACAA
L.chloranthus            ---------- ---------- ---------- ---------- ACCCTTATGG
L.pratensis              ---------- ---------- ---------- ---------- ACCCTTATGG
L.odoratus_HM            ---------- ---------- ---------- ---------- ACCCTTATGG
L.odoratus_kJ            ---------- ---------- ---------- ---------- ACCCTTATGG
L.hirsutus               ---------- ---------- ---------- ---------- ACCCTTATGG
L.annuus                 ---------- ---------- ---------- ---------- ACCCTTATGG
L.cirrhosus              TACACCGCCT AATAAT---- --AATGGTCG TATATAC--- ACCCTTATGG
L.latifolius             TACACCTCCT AATAAT---- --AATGGTCG TATATAC--- ACCCTTATGG
L.tingitanus             GAC---TCCT GTTAAT---- --ACTCGTCG TACATAC--- AACCTTATGG
Pisum sativum_H          GAGTTCTATT AATGCT---- -----AGTCC TCCACGC--- AACCGTCTAA
Pisum sativum_K          GAGTTCTATT AATGCT---- -----AGTCC TCCACGC--- AACCGTCTAA
Pisum sativum_HG         GAGTTCTGTT AATGCT---- -----AGTCC TCCACGC--- AACCGTCTAA
Pisum fulvum             GAGTTCTGTT AATGCT---- -----AGTCC TCCACGC--- AACCGTCTAA
Vavilovia formosa        GAGTTCTCTT AATGCT---- -----AGTCC TCCACGC--- AACTGTCTAA
L.clymenum               ---------- ---------- ---------- ---------- ----------
L.ochrus                 TTATATGAAA ACCAGA---- -----AAACA GGGGACC--- TTGCCCTTGA
```

	1001				
Trifolium strictum	AAAAAGCTTG	GGACTTGTCC	AGATTCTTGA	GTGTACCAAT	GGAAATCGTT
T. glanduliferum	AAAAAGCTTG	GGACTTGTCC	AGATTCTTGA	GTGTACCAAT	GGAAATCGTT
T. boissieri	AAAAAGCTTG	GGACTTGTCC	AGATTCTTGA	GTGTACCAAT	GGAAATCGTT
T. aureum	AAAAAGCTTG	GGACTTGTCC	AGATTCTTGA	GTGTACCAAT	GGAAATCGTT
T.meduseum	AAAAAGCTTG	GGACTTGTCC	AGATTCTTGA	GTATACCAAT	GGAAATCGTT
T.subterraneum	AAAAAGCTTG	GGACTTGTCC	AGATTCTTGA	GTATACCAAT	GGAAATCGTT
T.pratense	AAAAAGCTTG	GGACTTGTCC	AGATTCTTGA	GTATACCAAT	GGAAATCGTC
T.hybridum	AAAAAGCTTG	GGACTTATCC	AGATTCTTGA	GTGTACCAAT	GGAAATCGTT
T.semipilosum	AAAAAGCTTG	GGACTTGTCC	AGATTCTTGA	GTGTACCAAT	GGAAATCGTT
T.occidentale	AAAAAGCTTG	GGACTTGTCC	AGATTCTTGA	GTGTACCAAT	GGAAATCGTT
T.repens	AAAAAGCTTG	GGACTTGTCC	AGATTCTTGA	GTGTACCAAT	GGAAATCGTT
T.lupinaster	AAAAAGCTTG	GGACTTGTCC	AGATTCTTGA	GTGTACCAAT	GGAAATCGTT
Cicer oxyodon	AAAAAGCTTG	GGACTTGTCC	AGATTCTTGA	GTGTTCCAAT	GGAAATAGTT
C. chorrassanicum	AAAAAGCTTG	GGACTTGCCC	AGATTCTTGA	GTGTTCCAAT	GGAAATAGTC
C. arietinum	AAAAAGCTTG	GGACTTGTCC	AGATTCTTGA	GTGTACCAAT	GGAAATAGTT
Medicago truncatula_KF	AAAAAGCTTG	GGACTTGTCC	AGATTCTTGA	GTGTACCAAT	GGAAATCGTT
M.truncatula_AC	AAAAAGCTTG	GGACTTGTCC	AGATTCTTGA	GTGTACCAAT	GGAAATCGTT
M.sativa_K	AAAAAGCTTG	GGACTTGTCC	AGATTCTTGT	GTGTACCAAT	GGAAATCGTT
M.sativa	AAAAAGCTTG	GGACTTGTCC	AGATTCTTGT	GTGTACCAAT	GGAAATCGTT
M.papillosa	AAAAAGCTTG	GGACTTGTCC	AGATTCTTGT	GTGTACCAAT	GGAAATCGTT
M.hybrida	AAAAAGCTTG	GGACTTGTCC	AGATTCTTGT	GTGTACCAAT	GGAAATCGTT
M.falcata	AAAAAGCTTG	GGACTTGTCC	AGATTCTTGT	GTGTACCAAT	GGAAATCGTT
M.sativa_KU	AAAAAGCTTG	GGACTTGTCC	AGATTCTTGT	GTGTACCAAT	GGAAATCGTT
Melilotus albus	AAAAAGCTTG	GGACTTGTCC	AGATTCTTGA	GTGTACCAAT	GGAAATCGTT
Hedysarum formosum	AAACAGCTTC	GGACTTGGCT	GCATTCTTGG	ATGTACAATT	GGAAATAATT
H.varium	AAACAGCTTC	GGACTTGGCC	GCATTCTTGG	ATGTACAATT	GGAAATAATT
H.singarense	AAACAGCTTC	GGACTTGGCC	GCATTCTTGG	ATGTACAATT	GGAAATAATT
H.minjanense	AAACAGCTTC	GGACTTGGCC	GCATTCTTGG	ATGTACAATT	GGAAATAATT
Tavrniera glabra	AAACAGCTTC	GGACTTGGCC	GCATTCTTGG	ATGTACAATT	GGAAATAATT
T.diffusa	AAACAGCTTC	GGACTTGGCC	GCATTCTTGG	ATGTACAATT	GGAAATAATT
Greuteria membranacea	AAACAGCTTC	GGACTTGGCC	GCATTCTTGG	ATGTACAATT	GGAAATAATT
Corethrodendron scoparium	AAACAGCTTC	GGACTTGGCC	GCATTCTTGG	ATGTACAATT	GGAAATAATT
Eversmannia subspinosa	AAACAGCTTC	GGACTTGGCC	GCATTCTTGG	ATGTACAATT	GGAAATAATT
Onobrychis bungei	AAACAGCTTC	GGACTTGGCC	GCATTCTTGG	ATGTACAATT	GGAAATAATT
O.cornuta	AAACAGCTTC	GGACTTGGCC	GCATTCTTGG	ATGTACAATT	GGAAATAATT
O.michauxii	AAACAGCTTC	GGACTTGGCC	GCATTCTTGG	ATGTACAATT	GGAAATAATT
O.subacaulis	AAACAGCTTC	GGACTTGGCC	GCATTCTTGG	ATGTACAATT	GGAAATAATT
O.teheranica	AAACAGCTTC	GGACTTGGCC	GCATTCTTGG	ATGTACCATT	GGAAATAATT
Alhagi maurorum	AAACAGCTTC	GGACTTGGCC	GCATTCTTGG	ATGTACCATT	GGAAATAATT
Caragana microphylla	AAACAGCTTC	GGACTTGGCC	GCATTCTTGG	ATGTACCTTT	GGAAATAATT
C.korshinski	AAACAGCTTC	GGACTTGGCC	GCATTCTTGG	ATGTACCTTT	GGAAATAATT
C.kozlowii	AAACAGCTTC	GGACTTGGCC	GCATTCTTGG	ATGTACCATT	GGAAATAATT
C.rosea	AAACAGCTTC	GGACTTGGCC	GCATTCTTGG	ATGTACCATT	GGAAATAATT
Tibetia liangshanensis	AAACAGCTTC	GGACTTGGCC	GCATTCTTGG	ATGTACCTTT	GGAAATAATT
Gueldenstaedtia verna	----------	----------	----------	----------	----------
Halimodendron halodendron	AAACAGCTTC	GGACTTGGCC	GCATTCTTGG	ATGTACCGTC	GGAAATAATT
Smirnowia turkestana	AAACAGCTTG	GGACTTGTCC	GCATTCTTGG	ATGTACCGTT	GGAAATAATT
Eremosparton flaccidum	AAACAGCTTG	GGACTTGTCC	GCATTCTTGG	ACGTACCGTT	GGAAATAATT
Colutea persica	AAACAGCTTG	GGACTTGTCC	GCATTTTTGG	ATGTACCCTT	GGAAATAATT
C. triphylla	AAACAGCTTG	GGACTTGTCC	GCATTTTTGG	ATGTACCCTT	GGAAATAATT
Sphaerophysa salsula	AAACAACTTG	GGACTTGTCC	GCATTTTTGG	ATGTACCCTT	TGAAATAATT
Podlechiella vogelii	AAACAGCTTG	GGACTTGTCC	GCATTCTTGG	ATGTACCATT	GGAAATAATT
Carmichaelia australis	AAACAGCTTG	GGACTTGTCC	GCATTCTTGG	ATGTACCATT	AGAAATAATT
Sutherlandia frutescens	AAACAGCTTG	GGACTTGTCT	GCATTCTTGG	ATGTACCATT	GGAAATAATT
Astragalus membranaceous	AAACAGCTTG	GGACTTGTCC	GCATTCTTGG	ATGTACCATT	GGAAATAATT
A.mongholicus	AAACAGCTTG	GGACTTGTCC	GCATTCTTGG	ATGTACCATT	GGAAATAATT
A.nakaianus	AAACAGCTTG	GGACTTGTCC	GCATTTTTGG	ATGTACCCTT	GGAAATAATT
A.macropelmatus	AAACAGCTTG	GGACTTGTCC	GCATTCTTGG	ATGTACCATT	GGAAATAATT
A.iranicus	AAACAGCTTG	GGACTTATCC	GCATTCTTGG	ATGTACCATT	GGAAATAATT
A.denudatus	AAACAGCTTG	GGACTTGTCC	GCATTCTTGG	ATGTACCATT	GGAAATAATT
A.odoratus	AAACAGCTTG	GGACTTGTCC	GCATTCTTGG	ATGTACCATT	GGAAATAATT
Oxytropis szovitsii	AAACAGCTTG	GGACTTGTCC	GCATTCTTGG	ATGTACCATT	GGAAATAATT
O.iranica	AAACAGCTTG	GGACTTGTCC	GCATTCTTGG	ATGTACCATT	GGAAATAATT
O.kotschyana	AAACAGCTTG	GGACTTGTCC	GCATTCTTGG	ATGTACCATT	GGAAATAATT
Erophaca baetica	AAACAGCTTG	GGACTTGTCT	GCATTCTTGG	AAGTACCTTT	GGAAATTATT
Wisteria floribunda	AAAAAGCCTG	GGACTTGTCC	ATATTCTTGC	GTGTACCAAT	GGAAATATTT
W. sinensis	AAAAAGCCTG	GGACTTGTCC	ATATTCTTGC	GTGTACCAAT	GGAAATATTT
Glycyrrhiza lepidota	AAAAAGCTTG	GGACTTGTCC	ATATTCTTGC	GTGTACCAAT	GGAAAT---T
G.uralensis	AAAAAGCTTG	GGACTTGTCC	ATATTCTTGC	GTGTACCAAT	GGAAAT---T
G.glabra	AAAAAGCTTG	GGACTTGTCC	ATATTCTTGC	GTGTACCAAT	GGAAAT---T
Meristotropis xathioides	AAAAAGCTTG	GGACTTGTCC	ATATTCTTGC	GTGTACCAAT	GGAAAT---T
Robinia pseudoacacia	AAAAAGCTGG	AGAATTGTGT	AGGTTCTTGC	GTGTCCCAAT	TGAAATATTT
Lotus japonicus	AAAAAGCTGG	AGATTTGTCC	AAGTTATTGC	GTGTGCCAAT	TGAAATATTT
Vicia alpestris	AAAAAGCTTG	GGACTTGTCC	AGATTCATGA	GTGTACCAAT	GGAAATCGTT
V.sativa	AAAAAGCTTG	GGACTTGTCC	AGATTCTTGA	GTGTACCAAT	GGAAATCGTT
V.peregrina	AAAAAGCTTG	GGACTTGTCC	AGATTCTTGA	GTGTACCAAT	GGAAATCGTT
V.sepium	AAAAAGCTTG	GGACTTGTCC	AGATTCTTGA	GTGTACCAAT	GGAAATCGTT
V. faba	AAAAAGCTTG	GGACTTGTCC	AGATTCTTGA	GTGTACCAAT	GGAAATCGTT

V.canescens	AAAAAGCTTG	GGACTTGTCC	AGATTCTTGA	GTGTACCAAT	GGAAATCGTT
V.monantha	AAAAAGCTTG	GGACTTGTCC	AGATTCTTGA	GTGTACCAAT	GGTAATCGTT
V.tetrasperma	AAAAAGCTTG	GGACTTGTCC	AGATTCTTGA	GTGTACCAAT	GGAAATCGTT
V.narbonensis	AAAAAGCTTG	GGACTTGTCC	AGATTCTTGA	GTGTACCAAT	GGAAATCGTT
Lens culinaris	AAAAAGCTTG	GGACTTGTCT	AGATTCTTGA	GTGTACCAAT	GGAAATCGTT
L.orientalis	AAAAAGCTTG	GGACTTGTTT	AGATTCTTGA	GTGTACCCAT	GGAAATCGTT
V.ervilia	AAAAAGCTTG	GGACTTGTCC	AGATTCTTGA	GTGTACCAAT	GGAAATCGTT
Galega officinalis	AAAAAGCTTG	GGACTTGTCC	AGATTCTTGA	GTGTACCGAT	GGAAATCCTT
Lathyrus palustris_K	AAAAGGGGGC	GGAGTTGTCC	CGATTCTTGA	GTGTACCTAT	AAAAATTATT
L.palustris_H	AAAAGGGGGT	GGAGTTGTCC	CGATTCTTGA	GTGTACCCTT	AAAAATTATT
L.davidii	AAAAGGGGGA	GAAGTTGTCC	CGATTCTTGA	GTGTACCGCT	TAAAATTATT
L.japonicus	AAAAGGGGGC	GGAGTTGTCC	CGATTCTTGA	GTGTACCTCT	AAAAATTATT
L.ochroleucus	AAAAGGGGGC	GGAGTTGTCC	CGATTCTTGA	GTGTACCTCT	AAAAATTATT
L.littoralis	AAAAGGGGGC	GGAGTTGTCC	CGATTCTTGA	GTGTACCTCT	AAAAATTATT
L.venosus	AAAAGGGGGC	GGAGTTGTCC	CGATTCTTGA	GTGTACCTCT	AAAAATTATT
L.graminifolius	AAAAGGGGGC	GGAGTTGTCC	CGATTCTTGA	ATGTACCCCT	AAAAATTATT
L.pubescens	AAAAGGGTAC	GAAGTTGTCC	CGATTCTTGA	GTCTACCACT	CAAGATTGTT
L.sativus_KJ	AACATGCTTT	GGAGTTGTCC	AGATTCTTGA	ATATACCATT	GGACATTATT
L.sativus_HM	AACATGCTTT	GGAGTTGTCC	AGATTCTTGA	ATATACCATT	GGACATTATT
L.pseudocicera	AACATGGTTT	GGAGTTGTCC	AAATTCTTGA	ATATACCATT	GGAAATTAGT
L.chloranthus	AACATGGTGC	TGAATTGTCC	AGATTCTTGA	ATCTACCACT	CCAAGTCCTT
L.pratensis	AACATGGTGC	TGAATTGTCC	AGATTCTTGA	ATCTACCACT	CCAAGTCCTT
L.odoratus_HM	AACATGGTGC	TGAATTGTCC	AGATTCTTGA	ATCTACCACT	CCAAGTCCTT
L.odoratus_kJ	AACATGGTGC	TGAATTGTCC	AGATTCTTGA	ATCTACCACT	CCAAGTCCTT
L.hirsutus	AACATGGTGC	TGAATTGTCC	AGATTCTTGA	ATCTACCACT	CCAAGTACTT
L.annuus	AACATGGTGC	TGAATTGTCC	GGATTCTTGA	ATCTATCACT	CCAAGTCCTT
L.cirrhosus	AACATGGTGC	TGAATTGTCC	AGATTCTTGA	ATATACCACT	CCAAGTCCTT
L.latifolius	AACATGGTGC	TGAATTGTCC	AGATTCTTGA	ATATACCACT	CCAAGTCCTT
L.tingitanus	AAAAGGGTGT	GGAGTTGTCC	AGATTCTTGA	ATATACCACT	CGAAATTGTT
Pisum sativum_H	AAAAA-GTGT	GGAGTTGCCC	AGATTATTGA	----------	----------
Pisum sativum_K	AAAAA-GTGT	GGAGTTGCCC	AGATTATTGA	----------	----------
Pisum sativum_HG	AAAAA-GTGT	GGAGTTGCCC	AGATTATTGA	----------	----------
Pisum fulvum	AAAAA-GTGT	GGAGTTGCCC	AGATTATTGA	----------	----------
Vavilovia formosa	AAAAAAGTGT	GGAGTTGCCC	AGATTATTGA	----------	----------
L.clymenum	----------	----------	----------	----------	----------
L.ochrus	CTCCTGTTAA	GGATAGTCCT	GTATGCAACC	TTAGGCAAAA	TGGTGTGGAG

```
                        1051
Trifolium strictum         CCATATTCTT GA-------- ---------- ---------- ----------
T. glanduliferum           CCATATTCTT GA-------- ---------- ---------- ----------
T. boissieri               CCATATTCTT GA-------- ---------- ---------- ----------
T. aureum                  CCATATTCTT GA-------- ---------- ---------- ----------
T.meduseum                 CCGTATTCTT GA-------- ---------- ---------- ----------
T.subterraneum             CCGTATTCTT GA-------- ---------- ---------- ----------
T.pratense                 CCATATTCTT GA-------- ---------- ---------- ----------
T.hybridum                 CCATATTCTT GA-------- ---------- ---------- ----------
T.semipilosum              CCATATTCTT GA-------- ---------- ---------- ----------
T.occidentale              CCATATTCTT GA-------- ---------- ---------- ----------
T.repens                   CCATATTCTT GA-------- ---------- ---------- ----------
T.lupinaster               CCATATTCTT GA-------- ---------- ---------- ----------
Cicer oxyodon              CCATATTCTT GA-------- ---------- ---------- ----------
C. chorrassanicum          CCATATTCTT GA-------- ---------- ---------- ----------
C. arietinum               CCGTATTCTT GA-------- ---------- ---------- ----------
Medicago truncatula_KF     CCATATTCTT GA-------- ---------- ---------- ----------
M.truncatula_AC            CCATATTCTT GA-------- ---------- ---------- ----------
M.sativa_K                 CCATATTCTT GA-------- ---------- ---------- ----------
M.sativa                   CCATATTCTT GA-------- ---------- ---------- ----------
M.papillosa                CCATATTCTT GA-------- ---------- ---------- ----------
M.hybrida                  CCATATTCTT GA-------- ---------- ---------- ----------
M.falcata                  CCATATTCTT GA-------- ---------- ---------- ----------
M.sativa_KU                CCATATTCTT GA-------- ---------- ---------- ----------
Melilotus albus            CCATATTCTT GA-------- ---------- ---------- ----------
Hedysarum formosum         GTATAA---- ---------- ---------- ---------- ----------
H.varium                   GTTTAA---- ---------- ---------- ---------- ----------
H.singarense               GTATAA---- ---------- ---------- ---------- ----------
H.minjanense               GTATAA---- ---------- ---------- ---------- ----------
Tavrniera glabra           GTATAA---- ---------- ---------- ---------- ----------
T.diffusa                  GTATAA---- ---------- ---------- ---------- ----------
Greuteria membranacea      GTATAA---- ---------- ---------- ---------- ----------
Corethrodendron scoparium  GTATAA---- ---------- ---------- ---------- ----------
Eversmannia subspinosa     GTATAA---- ---------- ---------- ---------- ----------
Onobrychis bungei          GTATAA---- ---------- ---------- ---------- ----------
O.cornuta                  GTATAA---- ---------- ---------- ---------- ----------
O.michauxii                GTATAA---- ---------- ---------- ---------- ----------
O.subacaulis               GTCTAA---- ---------- ---------- ---------- ----------
O.teheranica               GTATAA---- ---------- ---------- ---------- ----------
Alhagi maurorum            GTATAA---- ---------- ---------- ---------- ----------
Caragana microphylla       GTATAA---- ---------- ---------- ---------- ----------
C.korshinski               GTATAA---- ---------- ---------- ---------- ----------
C.kozlowii                 GTATAA---- ---------- ---------- ---------- ----------
C.rosea                    GTATAA---- ---------- ---------- ---------- ----------
Tibetia liangshanensis     GTATAA---- ---------- ---------- ---------- ----------
Gueldenstaedtia verna      ---------- ---------- ---------- ---------- ----------
Halimodendron halodendron  GTATAA---- ---------- ---------- ---------- ----------
Smirnowia turkestana       GTATAA---- ---------- ---------- ---------- ----------
Eremosparton flaccidum     GTATAA---- ---------- ---------- ---------- ----------
Colutea persica            GTATAA---- ---------- ---------- ---------- ----------
C. triphylla               GTATAA---- ---------- ---------- ---------- ----------
Sphaerophysa salsula       GTATAA---- ---------- ---------- ---------- ----------
Podlechiella vogelii       GTATAA---- ---------- ---------- ---------- ----------
Carmichaelia australis     GTATAA---- ---------- ---------- ---------- ----------
Sutherlandia frutescens    GTATAA---- ---------- ---------- ---------- ----------
Astragalus membranaceous   GTATAA---- ---------- ---------- ---------- ----------
A.mongholicus              GTATAA---- ---------- ---------- ---------- ----------
A.nakaianus                GTATAA---- ---------- ---------- ---------- ----------
A.macropelmatus            GTATAA---- ---------- ---------- ---------- ----------
A.iranicus                 GTATAA---- ---------- ---------- ---------- ----------
A.denudatus                GTATAA---- ---------- ---------- ---------- ----------
A.odoratus                 GTATAA---- ---------- ---------- ---------- ----------
Oxytropis szovitsii        GTATAA---- ---------- ---------- ---------- ----------
O.iranica                  GTATAA---- ---------- ---------- ---------- ----------
O.kotschyana               GTATAA---- ---------- ---------- ---------- ----------
Erophaca baetica           GTATAA---- ---------- ---------- ---------- ----------
Wisteria floribunda        TGA------- ---------- ---------- ---------- ----------
W. sinensis                TGA------- ---------- ---------- ---------- ----------
Glycyrrhiza lepidota       TGA------- ---------- ---------- ---------- ----------
G.uralensis                TGA------- ---------- ---------- ---------- ----------
G.glabra                   TGA------- ---------- ---------- ---------- ----------
Meristotropis xathioides   TGA------- ---------- ---------- ---------- ----------
Robinia pseudoacacia       TGA------- ---------- ---------- ---------- ----------
Lotus japonicus            TGA------- ---------- ---------- ---------- ----------
Vicia alpestris            CCATATTCTT GA-------- ---------- ---------- ----------
V.sativa                   CCATATTCTT GA-------- ---------- ---------- ----------
V.peregrina                CCATATTCTT GA-------- ---------- ---------- ----------
V.sepium                   CCATATTCTT GA-------- ---------- ---------- ----------
V. faba                    CCATATTCTT GA-------- ---------- ---------- ----------
```

```
V.canescens               CCATATTCTT GA-------- ---------- ---------- ----------
V.monantha                CCATATTCTT GA-------- ---------- ---------- ----------
V.tetrasperma             CCATATTCTT GA-------- ---------- ---------- ----------
V.narbonensis             CCATATTCTT GA-------- ---------- ---------- ----------
Lens culinaris            CCATATTCGT GA-------- ---------- ---------- ----------
L.orientalis              CCAATTTCGT GA-------- ---------- ---------- ----------
V.ervilia                 CCGGATTCTT GA-------- ---------- ---------- ----------
Galega officinalis        CCATATTCTT GA-------- ---------- ---------- ----------
Lathyrus palustris_K      CAATTCAATT C-----CAAA GAATCAAAA- TAA------- ----------
L.palustris_H             CAATTCAATT C-----CAAA GAATCAAAA- TAA------- ----------
L.davidii                 CACTTCACTT CAATTGCAAA GAATCAAAAA TAA------- ----------
L.japonicus               CAATTCAATT C-----CAAA GAATCAAAA- TAA------- ----------
L.ochroleucus             CAATTCAATT C-----CAAA GAATCAAAA- TAA------- ----------
L.littoralis              CAATTCAATT C-----CAAA GAATCAAAA- TAA------- ----------
L.venosus                 CAATTCAATT C-----CAAA GAATCAAAA- TAA------- ----------
L.graminifolius           CAATTCAATT C-----CAAA GAATCAAAA- TAA------- ----------
L.pubescens               CAATCGAATT C-----CGAA GAATCCAAA- TAG------- ----------
L.sativus_KJ              TACAAAGAAT A-----CTAA ---------- ---------- ----------
L.sativus_HM              TACAAAGAAT A-----CTAA ---------- ---------- ----------
L.pseudocicera            TACGAAGAAT A-----CTAA ---------- ---------- ----------
L.chloranthus             GAAGAGGTTT A-----CTAC GAAGAATAA- ---------- ----------
L.pratensis               GAAGAGGTTT A-----CTAC GAAGAATAA- ---------- ----------
L.odoratus_HM             GAAGAGGTTT A-----CTAC GAAGAATAA- ---------- ----------
L.odoratus_kJ             GAAGAGGTTT A-----CTAC GAAGAATAA- ---------- ----------
L.hirsutus                GAAGAGGTTT A-----CTAC GAAGAATAA- ---------- ----------
L.annuus                  GAAGTGGTTT A-----CTAC GAAGAATAA- ---------- ----------
L.cirrhosus               GAAGAGGTTT A-----CTTC GAGGAAGAGG AAGAATAA-- ----------
L.latifolius              GAAGAGGTTT A-----CTTC GAGGAAGAGG AAGAATAA-- ----------
L.tingitanus              CAACCGAATT A-----CGAA TAA------- ---------- ----------
Pisum sativum_H           ---------- ---------- ---------- ---------- ----------
Pisum sativum_K           ---------- ---------- ---------- ---------- ----------
Pisum sativum_HG          ---------- ---------- ---------- ---------- ----------
Pisum fulvum              ---------- ---------- ---------- ---------- ----------
Vavilovia formosa         ---------- ---------- ---------- ---------- ----------
L.clymenum                ---------- ---------- ---------- ---------- ----------
L.ochrus                  TTGTCCAGAT TCTTGAGTGT ACGATTAATA ATTGTTGAAC CCGAATTACG
```

```
                        1101
Trifolium strictum         ---------- ---------- --------
T. glanduliferum           ---------- ---------- --------
T. boissieri               ---------- ---------- --------
T. aureum                  ---------- ---------- --------
T.meduseum                 ---------- ---------- --------
T.subterraneum             ---------- ---------- --------
T.pratense                 ---------- ---------- --------
T.hybridum                 ---------- ---------- --------
T.semipilosum              ---------- ---------- --------
T.occidentale              ---------- ---------- --------
T.repens                   ---------- ---------- --------
T.lupinaster               ---------- ---------- --------
Cicer oxyodon              ---------- ---------- --------
C. chorrassanicum          ---------- ---------- --------
C. arietinum               ---------- ---------- --------
Medicago truncatula_KF     ---------- ---------- --------
M.truncatula_AC            ---------- ---------- --------
M.sativa_K                 ---------- ---------- --------
M.sativa                   ---------- ---------- --------
M.papillosa                ---------- ---------- --------
M.hybrida                  ---------- ---------- --------
M.falcata                  ---------- ---------- --------
M.sativa_KU                ---------- ---------- --------
Melilotus albus            ---------- ---------- --------
Hedysarum formosum         ---------- ---------- --------
H.varium                   ---------- ---------- --------
H.singarense               ---------- ---------- --------
H.minjanense               ---------- ---------- --------
Tavrniera glabra           ---------- ---------- --------
T.diffusa                  ---------- ---------- --------
Greuteria membranacea      ---------- ---------- --------
Corethrodendron scoparium  ---------- ---------- --------
Eversmannia subspinosa     ---------- ---------- --------
Onobrychis bungei          ---------- ---------- --------
O.cornuta                  ---------- ---------- --------
O.michauxii                ---------- ---------- --------
O.subacaulis               ---------- ---------- --------
O.teheranica               ---------- ---------- --------
Alhagi maurorum            ---------- ---------- --------
Caragana microphylla       ---------- ---------- --------
C.korshinski               ---------- ---------- --------
C.kozlowii                 ---------- ---------- --------
C.rosea                    ---------- ---------- --------
Tibetia liangshanensis     ---------- ---------- --------
Gueldenstaedtia verna      ---------- ---------- --------
Halimodendron halodendron  ---------- ---------- --------
Smirnowia turkestana       ---------- ---------- --------
Eremosparton flaccidum     ---------- ---------- --------
Colutea persica            ---------- ---------- --------
C. triphylla               ---------- ---------- --------
Sphaerophysa salsula       ---------- ---------- --------
Podlechiella vogelii       ---------- ---------- --------
Carmichaelia australis     ---------- ---------- --------
Sutherlandia frutescens    ---------- ---------- --------
Astragalus membranaceous   ---------- ---------- --------
A.mongholicus              ---------- ---------- --------
A.nakaianus                ---------- ---------- --------
A.macropelmatus            ---------- ---------- --------
A.iranicus                 ---------- ---------- --------
A.denudatus                ---------- ---------- --------
A.odoratus                 ---------- ---------- --------
Oxytropis szovitsii        ---------- ---------- --------
O.iranica                  ---------- ---------- --------
O.kotschyana               ---------- ---------- --------
Erophaca baetica           ---------- ---------- --------
Wisteria floribunda        ---------- ---------- --------
W. sinensis                ---------- ---------- --------
Glycyrrhiza lepidota       ---------- ---------- --------
G.uralensis                ---------- ---------- --------
G.glabra                   ---------- ---------- --------
Meristotropis xathioides   ---------- ---------- --------
Robinia pseudoacacia       ---------- ---------- --------
Lotus japonicus            ---------- ---------- --------
Vicia alpestris            ---------- ---------- --------
V.sativa                   ---------- ---------- --------
V.peregrina                ---------- ---------- --------
V.sepium                   ---------- ---------- --------
V. faba                    ---------- ---------- --------
```

```
V.canescens                ---------- ---------- --------
V.monantha                 ---------- ---------- --------
V.tetrasperma              ---------- ---------- --------
V.narbonensis              ---------- ---------- --------
Lens culinaris             ---------- ---------- --------
L.orientalis               ---------- ---------- --------
V.ervilia                  ---------- ---------- --------
Galega officinalis         ---------- ---------- --------
Lathyrus palustris_K       ---------- ---------- --------
L.palustris_H              ---------- ---------- --------
L.davidii                  ---------- ---------- --------
L.japonicus                ---------- ---------- --------
L.ochroleucus              ---------- ---------- --------
L.littoralis               ---------- ---------- --------
L.venosus                  ---------- ---------- --------
L.graminifolius            ---------- ---------- --------
L.pubescens                ---------- ---------- --------
L.sativus_KJ               ---------- ---------- --------
L.sativus_HM               ---------- ---------- --------
L.pseudocicera             ---------- ---------- --------
L.chloranthus              ---------- ---------- --------
L.pratensis                ---------- ---------- --------
L.odoratus_HM              ---------- ---------- --------
L.odoratus_kJ              ---------- ---------- --------
L.hirsutus                 ---------- ---------- --------
L.annuus                   ---------- ---------- --------
L.cirrhosus                ---------- ---------- --------
L.latifolius               ---------- ---------- --------
L.tingitanus               ---------- ---------- --------
Pisum sativum_H            ---------- ---------- --------
Pisum sativum_K            ---------- ---------- --------
Pisum sativum_HG           ---------- ---------- --------
Pisum fulvum               ---------- ---------- --------
Vavilovia formosa          ---------- ---------- --------
L.clymenum                 ---------- ---------- --------
L.ochrus                   ACAAATCAAA AAAGCGAGAA TAGCATAG
```
