# Supplementary material for: Extensive survey of the ycf4 plastid gene throughout the IRLC legumes: Robust evidence of its locus and lineage specific accelerated rate of evolution, pseudogenization and gene loss in the tribe Fabeae
Source: PLoS One. 2020 Mar 5;15(3):e0229846. doi: 10.1371/journal.pone.0229846 (PMC7058334; doi:10.1371/journal.pone.0229846)
Supplement: S1 Table — (-) not available in GenBank. Abbreviations used in plant accession information: FMUH, Ferdowsi University of Mashhad Herbarium, Mashhad, Iran; GAZI, Gazi Universitesi Herbarium, Ankara, Turkey; IRAN, Iranian Research Institute of Plant Protection,Tehran, Iran; MO, Missouri Botanical Garden Herbarium, St Louis, USA; MSB Herbarium of Ludwig-Maximilians-Universitat, Munchen, Germany; TARI Herbarium of the Research Institute of Forests and Rangelands, Tehran, Iran; TMUH, Tarbiat Modares University Herbarium, Tehran, Iran; TUH, Tehran University Herbarium, Tehran, Iran; HWANRC Herbarium of West Azarbayjan Natural Resources Research Center, Urmia, Iran. aSequences from GenBank. bWhole plastid genome. (PDF) [file pone.0229846.s003.pdf]

S1 Table. Taxa included in the *ycf*4, *mat*K and *rpl*32 analyses.

Species	DNA source (location, voucher)	GenBank accession no. *ycf*4/*mat*K/*rpl*32
**Fabeae**		
Lathyrus alamutensis Mozaffarian et al.	Iran: Mozaffarian, Ahvazi & Charkhchian 88388 (TARI)	LC517983/LC518027/-
L. annuus L.	Iran: Hewer 3676 (TARI)	LC517984/LC518028/-
L. armenus (Boiss. &Huet) Celak.	Turkey: Aytaç 8317 (GAZI)	LC517985/LC518029/-
L. boissieri Sirj.	Iran: Runemark & Mozaffarian 29308 (TARI)	LC517986/LC518030/LC518058
L. brachypterus Celak.	Turkey: Aytaç & A. Duman 5441 (GAZI)	LC517987/LC518031/-
L. chloranthus Boiss.	Iran: Kazempour-Osaloo 2008-5 (TMUH)	LC517988/LC518032/-
L. cirrhosus Ser.	GenBank	HM029360[a]/-/-
*L. clymenum*L.	GenBank	KJ850235[b]
L. cyaneus (Steven) K.Koch.	Iran: Azerbayjan, Mozaffarian 70010 (TARI)	LC517989/LC518033/-
L. davidii Hance	GenBank	KJ806192[b]
L. digitatus (M. Bieb) Fiori	Turkey: M. Vural 4033 (GAZI)	LC517990/LC518034/-
L. graminifolius (S.Watson) T.G.White	GenBank	KJ806193[b]
L. hirsutus L.	Iran: Naqinezhad 27738 (TUH)	LC517991/AB935069[a]/-
L. japonicus Willd.	GenBank	KJ806195[b]
L. karsianus Davis	Turkey: G. Akgül 1700 (GAZI)	LC517992/LC518035/-
L. latifolius L.	GenBank	HM029359[a]/AF522085[a]/-
L. littoralis (Torr. &A.Gray) Walp.	GenBank	KJ806196[b]
L. ochroleucus Hook.	GenBank	KJ806198[b]
L. ochrus (L.) DC.	Turkey: H. Sagban 3137 (GAZI)	LC517993/AB935075[a]/LC518059
L. odoratus L.	GenBank	KJ850237[b]
L. odoratus L.	GenBank	HM029361[a]/-/-
L. palustris L.	GenBank	KJ806199[b]
L. palustris L.	GenBank	HM029362[a]/-/-
L. pratensis L.	Iran: Heidari et al. 1803 (HWANRC)	LC517994/LC518036/-
L. pseudocicera Pamp.	Turkey: N. Adiguzell 2238 (GAZI)	LC517995/LC518037/LC518060
L. pubescens Hook. &Arn.	GenBank	KJ806200[b]
L. sativus L.	GenBank	KJ806201[b]
L. sativus L.	GenBank	HM029371[b]
L. tingitanus L.	GenBank	KJ850238[b]
L. tukhtensis Czecz.	Turkey: A. Güner 5798 (GAZI)	LC517996/LC518038/LC518061
L. venosus Willd.	GenBank	KJ806202[b]
Lens culinaris Medik.	GenBank	KF186232[b]
L. orientalis (Boiss.) Schmalh.	Iran: Emadzadeh, Memariani & Zangooei 36153 (FUMH)	LC517997/-/LC518062
Pisumfulvum Sibth& Sm.	Turkey: Adiguzel & Aytaç 1896 (GAZI)	LC518008/LC518039/LC518063
P. sativum L.	GenBank	KJ806203[b]
P. sativum L.	GenBank	HM029370[b]
P. sativum L.	GenBank	HG966676[b]
Vavilovia formosa (Steven) Al.Fed.	Iran: Mozaffarian 88389 (TARI)	LC518014/LC518042/LC518066
Vicia alpestris Steven	Iran: Alizadeh et al. 8755 (HWANRC)	LC518015/LC518043/LC518067
V. canescens Labill.	Iran: Alizadeh et al. 5103 (HWANRC)	LC518016/LC518044/LC518068
V. ervilia (L.) Willd.	Iran: Emadzadeh, Memariani & Zangooei 36169 (FUMH)	LC518017/LC518045/-
V. faba L.	GenBank	KF042344[b]
V. monantha Retz.	Iran: Joharchi & Zangooei 16078 (FUMH)	LC518018/LC518046/LC518069
V. narbonensis L.	Iran: Alizadeh et al. 8760 (HWANRC)	LC518019/LC518047/LC518070

V. peregrina L.	Iran: Wendelbo & Assadi 11971 (TARI)	LC518020/LC518048/LC518071
V. sativa L.	GenBank	KJ850242[b]
V. sepium L.	GenBank	MG682352[b]
V. tetrasperma (L.) Schreb.	Iran: Pabot 7523 (TARI)	LC518021/-/LC518072
**Trifolieae**		
Medicago falcata L.	GenBank	KX831887[b]
M. hybrida (Pourr.) Trautv.	GenBank	KJ850240[b]
M. papillosa Boiss.	GenBank	KJ850241[b]
M. sativa L.	GenBank	KU321683[b]
M. sativa L.	GenBank	KU321071[b]
M. sativa L.	Iran: Kazempour-Osaloo 2017-1 (TMUH)	LC517999/-/-
M. truncatula Gaertn.	GenBank	AC093544[b]
M. truncatula Gaertn.	GenBank	KF241982[b]
Melilotus albus Medik.	GenBank	MH191352[b]
Trifolium aureum Pollich	GenBank	KC894708[b]
T. boissieri Guss.	GenBank	KJ788284[b]
T. glanduliferum Boiss.	GenBank	KJ788285[b]
T. hybridum L.	GenBank	KJ788286[b]
T. lupinaster L.	GenBank	KJ788287[b]
T. meduseum Blanche ex Boiss.	GenBank	KJ476730[b]
T. occidentale Coombe	GenBank	KJ788289[b]
T. pratense L.	GenBank	KJ788290[b]
T. repens L.	GenBank	KC894706[b]
T. semipilosum Fresen.	GenBank	KJ788291[b]
T. strictum L.	GenBank	KJ788292[b]
T. subterraneum L.	GenBank	EU849487[b]
Trigonella foenum-graecum L.	GenBank	MK460508[b]
**Galegeae**		
Galega officinalis L.	Georgia: Merello 2443 (MO)	LC517975/AB854574[a]/-
**Cicereae**		
Cicer arietinum L.	GenBank	EU835853[b]
C. chorassanicum (Bunge) Popov	Iran: Rafei & Zangooei 30566 (FUMH)	LC517967/AB198879[a]/-
C. oxyodon Boiss. & Hohen.	Iran: Kazempour-Osaloo 2009 (TMUH)	LC517968/AB198889[a]/-
**Hedysareae**		
Alhagi maurorum Medik.	Iran: Kazempour-Osaloo 2008-1 (TMUH)	LC517962/LC518022/LC518049
Corethrodendron scoparium (Fisch. & C.A.Mey.) Fisch. & Basiner	China: Xu et al. 86862 (MSB)	LC517971/AB854566[a]/LC137101[a]
Eversmannia subspinosa (DC.) B. Fedtsch.	Iran: Freitag & Mozaffarian 28397 (TARI)	LC517974/AB854573[a]/LC137102[a]
Greuteria membranacea (Coss. & Bal.) Amirahm. & Kaz. Osaloo (= *H .membranaceum* Coss. & Bal.)	Morocco: Podlech 49070 (MSB)	LC517976/AB854576[a]/LC137103[a]
Hedysarum formosum Fisch. & C.A.Mey. ex Basin.	Iran: Ghahreman & Mozaffarian 9778 (TUH)	LC517979/AB854585[a]/LC518055
H. minjanense Rech.f.	Tajikistan: Dickore 18281 (MSB)	LC517980/-/-
H. singarense Boiss. & Hausskn.	Iran: Babakhanlou 19984 (TARI)	LC517981/-/LC518056
H. varium Willd.	Turkey: Birden 1133 (GAZI)	LC517982/-/LC518057
Onobrychis aucheri subsp. teheranica (Bornm.) Rech.f.	Iran: Ahangarian & Kazempour-Osaloo 2005-1 (TMUH)	LC518001/AB854602[a]/LC137148[a]
O. bungei Boiss.	Iran: Rechinger 43484 (MSB)	LC518002/-/LC137113[a]
O. cornuta (L.) Desv.	Iran: Kazempour-Osaloo 2009-1 (TMUH)	LC518003/JQ669622[a]/LC137115[a]
O. michauxii DC.	Iran: Assadi 86612 (TARI)	LC518004/JQ669628[a]/LC137129[a]
Taverniera cuneifolia (Roth) Arn.	Iran: Basiri & Norouzi 1531 (TUH)	LC518012/LC518040/LC518064
T. diffusa (Cambess.) Thulin	Pakistan: Lamond 1405 (MSB)	LC518013/LC518041/LC518065
**Caraganeae**		
Caragana korshinskii Kom.	GenBank	KX289923[b]
C. kozlowii Kom.	GenBank	KX349219[b]
C. microphylla Lam.	GenBank	KX289922[b]

C. rosea Maxim.	GenBank	MF593790[b]
Gueldenstaedtia verna (Georgi) Boriss.	USSR: Ulanova & Bassargin 39025(MSB)	LC517977/-/-
Halimodendron halodendron (Pall.) Voss	Iran: Maassoumi et al. 86046 (TARI)	LC517978/JQ619947[a]/LC518054
Tibetia liangshanensis P.C.Li	GenBank	MF193597[b]
**Coluteae**		
Carmichaelia australis R.Br.	GenBank	MF597719[b]
Colutea persica Boiss.	Iran: Manucheri et al. 277(TARI)	LC517969/LC164595[a]/LC164615[a]
C. triphylla Bunge ex Boiss.	Iran: Assadi & Mozaffarian 33325 (TARI)	LC517970/LC085323[a]/LC164617[a]
Eremosparton flaccidum Litw.	Turkmanistan: Beljanina et al. 9353 (MSB)	LC517972/LC164597[a]/LC164619[a]
Lessertia frutescens (L.) Goldblatt & J.C.Manning	South Africa: Mummenhoff 851 (TMUH)	LC517998/LC164604[a]/LC164631[a]
Podlechiella vogelii (Webb) Maassoumi & Kaz. Osaloo	Algeria: Podlech 36700 (TARI)	LC518009/LC164600[a]/LC164627[a]
Smirnowia turkestana Bunge	Iran: Maddah & Moradi 3981 (TARI)	LC518010/LC164601[a]/LC164628[a]
Sphaerophysa salsula (Pall.) DC.	Iran: Azimi & Talaii 3687 (TMUH)	LC518011/LC164603[a]/LC164630[a]
**Wisterieae**		
Wisteria floribunda (Willd.) DC.	GenBank	KM103376[b]
W. sinensis (Sims) Sweet	GenBank	KT200359[b]
Astragalus/Oxytropis/Erophaca		
Astragalus denudatus Steven	Iran: Kazempour-Osaloo 2016-1 (TMUH)	LC517963/LC518023/LC518050
A. iranicus Bunge	Iran: Kazempour-Osaloo 2016-2 (TMUH)	LC517964/LC518024/LC518051
A. macropelmatus Bunge	Iran: Kazempour-Osaloo 2016-3 (TMUH)	LC517965/LC518025/LC518052
A. membranaceus (Fisch.) Bunge	GenBank	KX255662[b]
A. mongholicus Bunge	GenBank	KU666554[b]
A. nakaianus Y.N. Lee	GenBank	KR296789[b]
A. odoratus Lam.	Iran: Kazempour-Osaloo 2016-4 (TMUH)	LC517966/LC518026/LC518053
Erophaca baetica (L.) Boiss.	Turkey: Nydeyyer 40164 (MSB)	LC517973/JQ619937[a]/-
Oxytropis iranica Vassilcz.	Iran: Behboudi & Aellen 1948, 5598E (IRAN)	LC518005/-/-
O. kotschyana Boiss. & Hohen.	Iran: Shahi Shavvon & Kazempour-Osaloo 2014s50/2 (TMUH)	LC518006/-/-
O. szovitsii Boiss. & Buhse	Iran: Termeh et al. 33756E (IRAN)	LC518007/-/LC164624[a]
Glycyrrhiza/Meristotropis		
Glycyrrhiza glabra L.	GenBank	KF201590[b]
G. lepidota Pursh	GenBank	KY038482[b]
G. uralensis Fisch.	GenBank	KU862308[b]
Meristotropis xanthioides Vassilcz.	Iran: Ghahreman et al. 27652 (TUH)	LC518000/-/-
**Outgroups**		
Robinia pseudoacacia L.	GenBank	KJ468102[b]
Lotus japonicas (Regel) K.Larsen	GenBank	AP002983[b]

(-) not available in GenBank

Abbreviations used in plant accession information: FUMH, Ferdowsi University of Mashhad Herbarium, Mashhad, Iran; GAZI, Gazi Universitesi Herbarium, Ankara, Turkey; IRAN, Iranian Research Institute of Plant Protection,Tehran, Iran; MO, Missouri Botanical Garden Herbarium, St Louis, USA; MSB, Herbarium of Ludwig-Maximilians-Universitat, Munchen, Germany; TARI, Herbarium of the Research Institute of Forests and Rangelands, Tehran, Iran; TMUH, Tarbiat Modares University Herbarium, Tehran, Iran; TUH, Tehran University Herbarium, Tehran, Iran; HWANRC, Herbarium of West Azarbayjan Natural Resources Research Center, Urmia, Iran.

[a]Sequences from GenBank

[b]Whole plastid genome
