# Supplementary material for: Extensive survey of the ycf4 plastid gene throughout the IRLC legumes: Robust evidence of its locus and lineage specific accelerated rate of evolution, pseudogenization and gene loss in the tribe Fabeae
Source: PLoS One. 2020 Mar 5;15(3):e0229846. doi: 10.1371/journal.pone.0229846 (PMC7058334; doi:10.1371/journal.pone.0229846)
Supplement: S2 Table — * Location indicates the Start and end nucleotide positions. (PDF) [file pone.0229846.s004.pdf]

S2 Table. Location and base composition of amplification and sequencing primers used in this study.

Primer name	Primer sequence	Location*	Source
*acc*D-F	5'-AAACAGGCACAGGTCAASTAAATGG-3'	842-866	Magee et al. 2010
*Cem*A-R	5'-GACGGAGATACACGATTTAAATAACG-3'	631-656	Magee et al. 2010
*psa*I-F1	5'-CCTTCCNTTTTTGTKCCTTTAGTR-3'	59-82	Present study
*cem*A-R1	5'-TAAATGTWAAAGAGATGCACCAGG-3'	631-656	Present study

* Location indicates the Start and end nucleotide positions.
