# Supplementary material for: Extensive survey of the ycf4 plastid gene throughout the IRLC legumes: Robust evidence of its locus and lineage specific accelerated rate of evolution, pseudogenization and gene loss in the tribe Fabeae
Source: PLoS One. 2020 Mar 5;15(3):e0229846. doi: 10.1371/journal.pone.0229846 (PMC7058334; doi:10.1371/journal.pone.0229846)
Supplement: S3 Table — (PDF) [file pone.0229846.s005.pdf]

S3 Table. Parameter estimation and likelihood ratio tests for the branch-site model.

Gene	Model	Np	Ln L	Estimates of parameters	LRT *p*-Value	Positive Sites (BEB)
*ycf*4	Model A	115	-346.334043	*p*0 = 0.0<br>*p*1 = 0.0<br>*p*2a = 0.20401<br>*p*2b = 0.79599<br>ω0 = 0.0<br>ω1 = 1.00000<br>ω2a = 5.36021<br>ω2b = 5.36021	0.031	1 L 0.977*, 2 S 0.993**, 3 V 0.999**, 4 V 1.000**, 5 L 0.971*, 6 L 0.997**, 7 T 0.995**
*ycf*4	Model A Null	114	-348.633693	*p*0 = 0.0<br>*p*1 = 1.00000<br>*p*2a = 0.0<br>*p*2b = 0.0<br>ω0 = 0.0<br>ω1 = 1.00000<br>ω2a = 1.00000<br>ω2b = 1.00000		Not Allowed
*mat*K	Model A	69	-7469.753525	*p*0 = 0.69266<br>*p*1 = 0.30734<br>*p*2a = 0.0<br>*p*2b = 0.0<br>ω0 = 0.12324<br>ω1 = 1.00000<br>ω2a = 1.00000<br>ω2b = 1.00000	0.994	Not Allowed
*mat*K	Model A Null	68	-7469.753548	*p*0 = 0.69266<br>*p*1 = 0.30734<br>*p*2a = 0.0<br>*p*2b = 0.0<br>ω0 = 0.12324<br>ω1 = 1.00000<br>ω2a = 1.00000<br>ω2b = 1.00000		Not Allowed
*rpl*32	Model A	162	-1384.373553	*p*0 = 0.76461<br>*p*1 = 0.23539<br>*p*2a = 0.0<br>*p*2b = 0.0<br>ω0 = 0.11327<br>ω1 = 1.00000<br>ω2a = 1.00000<br>ω2b = 1.00000	1.00	Not Allowed
*rpl*32	Model A Null	161	-1384.373553	*p*0 = 0.76461<br>*p*1 = 0.23539<br>*p*2a = 0.0<br>*p*2b = 0.0<br>ω0 = 0.11327<br>ω1 = 1.00000<br>ω2a = 1.00000<br>ω2b = 1.00000		Not Allowed
